# Assembly of the catalytic module and the rotor of human ATP synthase

**DOI:** 10.1038/s44318-026-00842-9

**Published:** 2026-06-26

**Authors:** Jiuya He, Joe Carroll, Shujing Ding, Jiao Li, Ian M Fearnley, John E Walker

**Affiliations:** 1 Medical Research Council Mitochondrial Biology Unit, University of Cambridge, Cambridge Biomedical Campus, Cambridge, UK; 2 School of Medicine, Nanjing University of Chinese Medicine, Nanjing, China; 3 Present Address: State Key Laboratory of Green Pesticide, Centre for R&D of Fine Chemicals of Guizhou University, Guiyang, China; 4 Present Address: Division of Life Sciences and Medicine, University of Science and Technology of China, Hefei, China

**Keywords:** Metabolism, Organelles, Structural Biology

## Abstract

Human ATP synthase is a molecular rotary machine bound in inner mitochondrial membranes, built from twenty-eight subunits of seventeen kinds, two encoded in mitochondrial DNA, the remainder in nuclear genes. The machine consists of a rotor and an interacting stator. Turning of the rotor driven by a transmembrane proton motive force effects a cycle of structural changes in the catalytic part of the stator, producing three ATP molecules per rotation. Here, to establish how the stator and rotor are assembled, we deleted subunits and known assembly factors from human cells, purified and accumulated assembly intermediate complexes, and characterized them by gel analysis and mass spectrometry, allowing us to propose pathways of assembly of the rotor and the catalytic F_1_-module of the stator. These observations provide opportunities for further development by structural analysis of the accumulated intermediates. The compositions of the various assembly intermediates support the view that ATP synthase arose via independent evolution of its three constituent structural components, the catalytic F_1_-module, the peripheral stalk module, and the membrane-associated F_o_-module.

## Synopsis

Human ATP synthase is a molecular rotary machine built from twenty-eight subunits of seventeen kinds, two encoded in mitochondrial DNA, fifteen in the nucleus. Deletion of subunits and assembly factors and product analysis shows that assembly proceeds via constituent structural modules.

- The catalytic F_1_-module can be assembled independently of other subunits of the enzyme.
- The F_1_-module is built by introduction of the three α-subunits and the three β-subunits in alternation around either a pre-assembled central stalk or a preassembled rotor complex.
- The assembly factor assisted entry of α- and β-subunits proceeds via different mechanisms.
- The assembly process avoids making intermediate complexes that could either hydrolyze ATP or uncouple mitochondria by dissipating the proton motive force.
- The modular assembly process supports the modular evolution of ATP synthases.

## Abstract (web summary)

Deletion of subunits and assembly factors and product analysis shows that assembly of mitochondrial ATP synthase proceeds via constituent structural modules.

## Graphical abstract

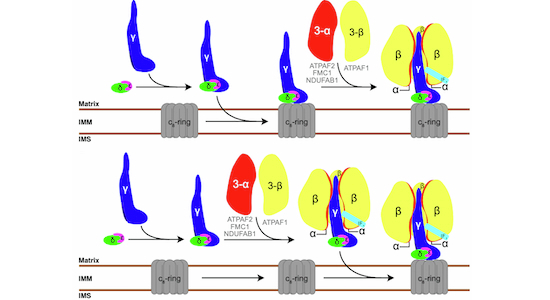

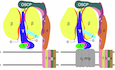

## Introduction

**Figure 1:**
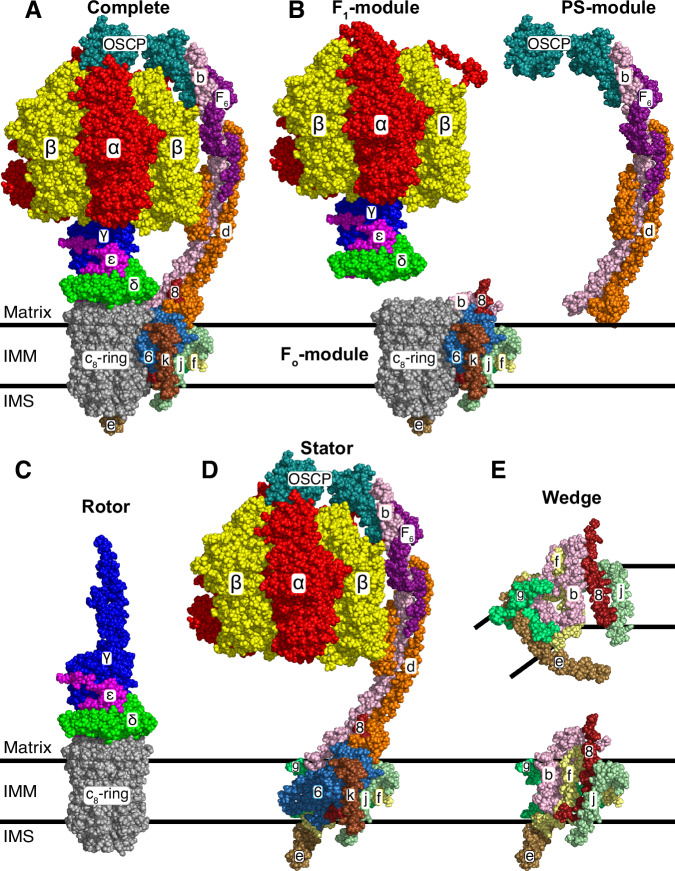
Structure of the bovine ATP synthase and its constituent modules, domains, and functional units. The intact monomeric bovine enzyme (PDB 6ZQM) and the human enzyme are identical in subunit composition and very similar in structure (Spikes et al, 2020; Lai et al, 2023). (**A**) Intact bovine complex (PDB 6ZQM) bound in the IMM with minor regions in the inter-membrane space (IMS). IF_1_ present in PDB 6ZQM has been removed. (**B**) The three constituent modules. The catalytic F_1_-module is built from three α-subunits and three β-subunits plus single copies of subunits γ, δ, and ε, which form the central stalk (CS) domain. The membrane F_o_-module consists of the c_8_-ring associated with ATP6, plus subunits ATP8, e, f, g, k, and j, and the two transmembrane α-helices of subunit b. The peripheral stalk (PS) module is made of single copies of subunits OSCP, b (membrane extrinsic portion), d, and F_6_. (**C**) The rotor made of the CS bound to the c_8_-ring. (**D**) The stator, made of the α_3_β_3_-domain, the PS, and subunits of F_o_. (**E**) Two views of the wedge consisting of two transmembrane α-helices of subunit b plus subunits e, f, and g, and encapsulated specific phospholipids with subunits j and ATP8 bound to its external surface. The upper representation is rotated by 90° around the y-axis relative to the lower image and indicates the bend in the IMM at the cristae tips. Source data are available online for this figure.

Human mitochondrial ATP synthase (h-ATP synthase) sustains life by making about 50 kg of ATP daily per person (Walker, 1998, 2017). It is a molecular machine where ATP synthesis from ADP and inorganic phosphate is driven by a rotor powered by a proton motive force (pmf) across the inner mitochondrial membrane (IMM). The pmf is generated by the outward proton pumping of respiratory enzymes (Mitchell, 1961), with energy derived from the oxidation of sugars and fats. Each ATP synthase complex is an assembly of 28 subunits of 17 kinds (Fig. 1A). Two membrane subunits, ATP6 and ATP8, encoded in mitochondrial DNA, are synthesized inside mitochondria (Fearnley and Walker, 1986), whereas the 15 nuclear encoded subunits are imported into the organelle. The main question we are investigating is: how is the ATP synthase built from these 17 components, emanating from two distinct cellular sources, three of them assembled in multiple copies? Related subsidiary questions are: how does this assembly process avoid building incomplete and uncoupled intermediate ATP synthase precursors that could wastefully either hydrolyze ATP or dissipate the proton-motive force, and does the way that the enzyme is assembled reflect how it might have evolved?

The structure of the human enzyme (Lai et al, 2023), which is closely related to that of the extensively studied bovine ortholog (Spikes et al, 2020), is central to understanding these questions. The monomeric complex (Fig. 1A) consists of three modules (Fig. 1B, see Table 1 for descriptions of the “modules” and “domains” and their roles). They are known historically as the membrane extrinsic F_1_-catalytic module (Fig. 1B, upper left) extending into the mitochondrial matrix (Penefsky et al, 1960; Pullman et al, 1960), the membrane intrinsic F_o_ module (Fig. 1B, bottom), to which F_1_ is bound (Kagawa and Racker, 1966a, 1966b), and the peripheral stalk (PS) module (Fig. 1B, right) (Collinson et al, 1994; Dickson et al, 2006), linking the external surface of F_1_ to F_o_ via two transmembrane α-helices in the N-terminal region of the b-subunit (Spikes et al, 2020). The F_1_-module comprises a sphere of three non-catalytic α-subunits and three catalytic β-subunits arranged alternately around an asymmetrical central stalk (CS) domain that consists of an elongated γ-subunit with single δ- and ε-subunits attached to its foot (Gibbons et al, 2000). In the intact enzyme, the CS-domain is associated via this foot to the c_8_-ring component of the F_o_-module (Fig. 1A), and, together, the CS and the c_8_-ring constitute the enzyme’s rotor (Fig. 1C) (Watt et al, 2010) (Fig. 1A,C). The enzyme’s stator (Fig. 1D) consists of the catalytic α_3_β_3_-domain, plus the PS- and F_o_-modules minus the c_8_-ring. The PS-module is attached to the top of the α_3_β_3_-domain via the OSCP (oligomycin sensitivity conferral protein), which interacts with the C-terminal region of the b-subunit (Rees et al, 2009). A long α-helix in subunit b extends downwards in association with the d and F_6_ subunits and enters the IMM, where its two N-terminal transmembrane α-helices, and associated subunits e, f, and g, form a wedge-shaped domain (the “wedge”) (Fig. 1E) that encapsulates specific phospholipids (Spikes et al, 2020). The crucial stator component, ATP6, is bound to the wedge and provides two proton half channels involved in the generation of rotation. Subunit ATP8 augments the wedge, subunit j stabilizes the association of ATP6 with the wedge, and subunit k appears to support the interaction of ATP6 with the c-ring, and together with subunit g, it may be involved in linking adjacent dimer complexes together in the formation of the dimer rows along the tips of the cristae in the IMM (Dudkina et al, 2006; Strauss et al, 2008; Dudkina et al, 2010; Davies et al, 2011; He et al, 2018; Pinke et al, 2020; Spikes et al, 2021). During ATP synthesis, the central rotor turns against the stator in a counter-clockwise direction (as viewed from the matrix) such that its asymmetric CS-domain elicits a cycle of structural changes in the three catalytic sites in the β-subunits of the stator, resulting in the binding of three molecules each of ADP and phosphate and the synthesis of three ATP molecules per rotation (Abrahams et al, 1994; Bowler et al, 2007).

**Table 1:** Subunits of the h-ATP synthase: their spatial organization into modules and domains, and their functions.

Subunit	Gene	Module^a^	Domain	Functional Unit (role)
α	ATP5F1A	F_1_	α_3_β_3_	Stator (non-catalytic nucleotide sites)
β	ATP5F1B	F_1_	α_3_β_3_	Stator (catalytic nucleotide sites)
γ	ATP5F1C	F_1_	CS	Rotor
δ	ATP5F1D	F_1_	CS	Rotor
ε	ATP5F1E	F_1_	CS	Rotor
c	ATP5MC1-3	F_o_	c_8_-ring	Rotor (H^+^-carrier)
ATP6^b^	MT-ATP6	F_o_	Membrane	Stator (H^+^-half channels)
ATP8^b^	MT-ATP8	F_o_	Membrane	Stator
e	ATP5ME	F_o_	Wedge	Stator (dimer angle)
f	ATP5MF	F_o_	Wedge	Stator (dimer angle)
g	ATP5MG	F_o_	Wedge	Stator (row dimerization, dimer angle)
j^b^	ATP5MJ	F_o_	Membrane	Stator
k^b^	ATP5MK	F_o_	Membrane	Stator (row dimerization)
OSCP	ATP5PO	PS	PS	Stator
b	ATP5PB	PS	PS, wedge	Stator (dimer angle)
d	ATP5PD	PS	PS	Stator
F_6_	ATP5PF	PS	PS	Stator

^a^F_1_, catalytic module made of the α_3_β_3_ “head” domain penetrated by the central stalk domain (CS); PS, peripheral stalk module tethering the α_3_β_3_ catalytic part of the stator to the membrane domain; F_o_, module composed of domains that are parts of the rotor (c_8_-ring), of two proton translocation half-channels (ATP6), and of the wedge that, in the dimer, provides the interface between monomers, and fixes the rotational axes of the monomers at a specific angle, promoting curvature of the IMM. Membrane subunits g and k probably provide side-to-side linkages between dimers in the dimer chains at the tips of the cristae.

^b^Other common names for ATP6, ATP8, j and k are subunit a, A6L, 6.8PL and DAPIT, respectively.

**Figure EV1:**
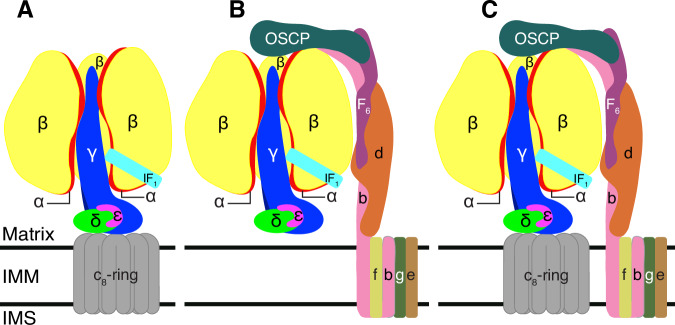
Key sub-complexes of human ATP synthase known to be assembled in the absence of specific subunits. The upper region of the CS has been exposed by the removal of an α-subunit (red). IF_1_ is bound to all three complexes to prevent ATP hydrolysis. (**A**) Complex F_1_-c_8_ assembled in the absence of any one of the PS components OSCP, subunit b, or subunit F_6_ (He et al, 2017a, 2020). (**B**) Complex F_1_-PS plus e, f, and g, assembled in the absence of subunit c (He et al, 2017b). (**C**) Complex (referred to as “the key intermediate”) assembled in the absence of mitochondrially encoded subunits ATP6 and ATP8 (He et al, 2017b), and arrived at by the addition of either the PS plus associated membrane subunits e, f, and g to intermediate (**A**), or the c_8_-ring to complex (**B**).

In previous studies of how this complex machine is put together, we showed that, first, formation of the c_8_-ring is assisted by two assembly factor (AF) proteins, by TMEM70, in confirmation of a previous study (Kovalčíková et al, 2019), and by TMEM242 (Carroll et al, 2021), and, second, that in the absence of any one of the three PS components, OSCP, b and F_6_, human cells assemble an F_1_-c_8_ complex (Fig. EV1A) (He et al, 2017a, 2020). Also, we have shown that deletion of the fourth PS constituent, subunit d, led to the accumulation of the larger vestigial complex, F_1_-c_8_-OSCP-F_6_-b-e-f-g (He et al, 2020), whereas in a similar independent experiment, the simpler F_1_-c_8_ complex had been observed (Fujikawa et al, 2015). Third, in the absence of subunit c, we found that an F_1_-PS complex plus subunits e, f, and g had been built (Fig. EV1B) (He et al, 2017b). These observations imply, but do not prove, that the F_1_-module and the c_8_-ring components of the rotor are assembled independently. We confirmed that the initial stages of the assembly of the PS are independent of F_1_ and the c_8_-ring (Fujikawa et al, 2015; He et al, 2020), and demonstrated that the PS can associate with either the F_1_-domain or F_1_-c_8_ via two alternative pathways (He et al, 2020). Crucially, we established that in ρ^0^ cells (devoid of mitochondrial DNA, and therefore lacking subunits ATP6 and ATP8), a complex named the ‘key intermediate’ in view of its importance, is assembled (Fig. EV1C) (He et al, 2017b). This almost complete enzyme complex, built entirely of nuclear encoded proteins, provides a template to accept mitochondrially encoded ATP6 into the protein void between the c_8_-ring and the wedge with the assistance of mitochondrially encoded ATP8 and nuclear encoded subunit j (He et al, 2020). This insertion generates the contact between the membrane parts of the rotor and stator, and provides the two requisite proton half-channels, thus completing the active enzyme.

Here, we have established more central features of how the h-ATP synthase is built from its 27 constituent proteins, by demonstrating independent assembly of the F_1_-module and the CS, and by uncovering how the rotor itself is assembled from the CS and the c_8_-ring. These findings support the notion that the ATP synthase evolved in a modular fashion (Walker and Cozens, 1986). Our experimental approach is the disruption of genes for subunits of the h-ATP synthase (singly or together) and for AFs, purification of accumulated protein assembly intermediate complexes via immunocapture or incorporated affinity tags, and characterization of the complexes by gel electrophoresis and quantitative mass spectrometry (MS).

## Results

### Independent assembly of the h-F_1_-module

**Figure EV2:**
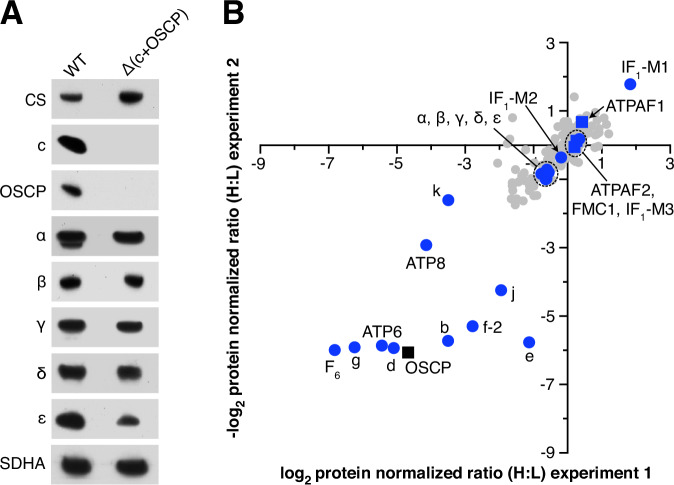
Impact of deleting the OSCP and the c-subunit of h-ATP synthase together in HEK293 cells. (**A**) Western blots of DDM extracts of mitoplasts from HEK293-WT and -Δ(c+OSCP) cells, with antibodies against the subunits indicated on the left. Citrate synthase (CS) and the SDHA subunit of complex II are loading controls. (**B**) Protein abundances in immunopurified ATP synthase from HEK293-Δ(c+OSCP) cells relative to HEK293-WT cells determined by SILAC-MS. The experiment was performed twice (biological replicates) with reciprocal SILAC labeling. The points on the scatter plots represent the log base 2 values of protein ratios from the two complementary MS analyses, normalized by centering on the median protein ratio. IF_1_-M1, -M2, and -M3 are specific mature forms of IF_1_ (He et al, 2017b); f-2 is an isoform of subunit f (UniProt P56134). Protein ratios were derived from a minimum of two peptide ratios, except ATP6 and ATPAF2 in experiment 1 where the value is derived from a single peptide ratio. No ratios were obtained for subunit c. Blue circles, ATP synthase subunits and forms of IF_1_; blue squares, assembly factors ATPAF1, ATPAF2, and FMC1; black square, deleted subunit; gray circles, all other identified proteins.

**Figure 2:**
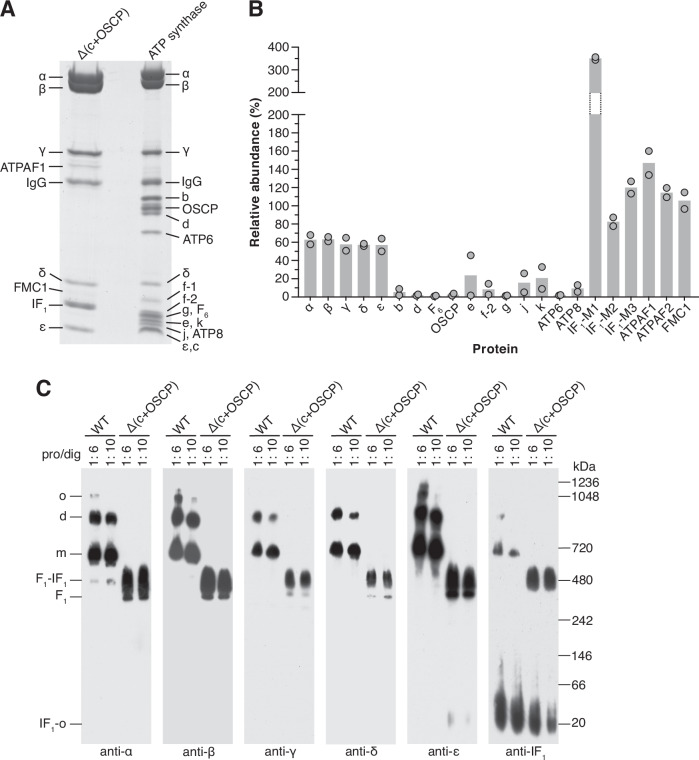
Assembly of the F_1_-module of h-ATP synthase in the absence of subunits c and OSCP. (**A**) SDS-PAGE analysis of F_1_-module immunopurified from n-dodecyl-β-D-maltoside (DDM) extracts of mitoplasts from HEK293-Δ(c+OSCP) cells and of h-ATP synthase. F_1_ was extracted and purified in the presence of 1 mM MgCl_2_ and 0.2 mM ATP. Proteins were detected with Coomassie blue. The positions of proteins are indicated (Appendix Table S1). IgG immunoglobulin light chain. (**B**) Abundances determined by SILAC-MS of subunits in immunopurified F_1_ relative to HEK293-WT cells. The experiment was performed twice (biological replicates) with reciprocal SILAC labeling. Histograms represent the median normalized relative abundances (% subunit abundance, *n* = 2) and show the two experimental values (gray circles) of the subunits of h-ATP synthase and AFs for the F_1_-domain. IF_1_-M1, -M2, and -M3 are three mature forms of IF_1_ (He et al, 2017b); f-2 is an isoform of subunit f (UniProt P56134). Protein ratios were derived from a minimum of two peptide ratios except for ATP6 and ATPAF2 which came from single values in one of the two experiments. (**C**) BN-PAGE analysis of the same amounts of protein at various protein:digitonin ratios. ATP synthase and vestigial complexes were detected by western blotting with antibodies against individual α, β, γ, δ, ε subunits, and IF_1_. o, d, and m, oligomeric, dimeric, and monomeric ATP synthase, respectively. The positions of the F_1_-IF_1_ complex, F_1_, and oligomeric IF_1_ (IF_1_-o) (Boreikaite et al, 2019) are shown on the left, and molecular weight markers on the right. Source data are available online for this figure.

The three genes for the c-subunit and the gene for the OSCP were disrupted together by CRISPR-Cas9 gene editing in Flp-In^TM^T-REx^TM^ HEK293 cells (referred to as HEK293-WT cells), and their absence from the resulting HEK293-Δ(c+OSCP) cells was verified (Fig. EV2; Appendix Figs. S1 and S2). With an ATP synthase immunopurification kit to capture the complexes, it was found that the F_1_-module plus IF_1_ had been assembled independently of all other subunits (Fig. 2A–C; Appendix Table S1) at *ca*. 50% of the level of the intact enzyme in WT cells. Also, AF proteins for F_1_, namely ATPAF1, ATPAF2, and FMC1 (Wang et al, 2001; Li et al, 2017) were detected at low levels (Fig. 2A) and were enriched relative to the captured F_1_ module components (Fig. 2B), suggesting that the extract might contain partial assemblies of the F_1_ catalytic module with bound AFs.

### The CS and the rotor complex assemble in the absence of α- and β-subunits

**Figure 3:**
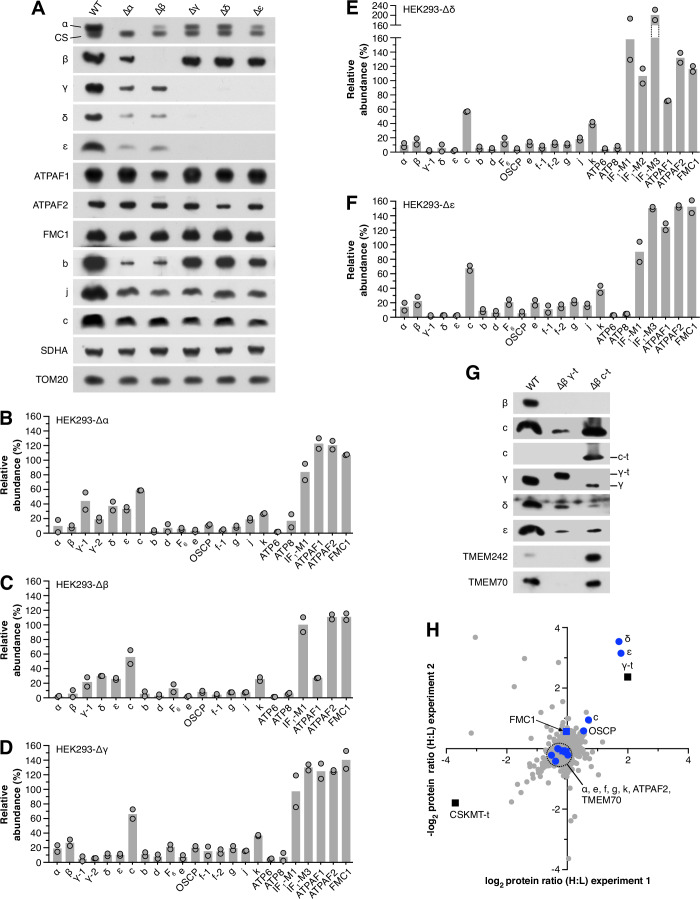
Impact of individual deletion of the α-, β-, γ-, δ-, and ε-subunits of the F_1_-module of human ATP synthase and formation of the rotor complex. (**A**) SDS-PAGE fractionation of DDM extracts of mitoplasts from HEK293-WT, -∆α, -∆β, -∆γ, -∆δ, and -∆ε clonal cells. Proteins were transferred to membranes and probed with antibodies against proteins indicated on the left. Citrate synthase (CS), TOM20, and SDHA are loading controls. In the -Δγ lane, subunits δ- and ε- were detected weakly, possibly because the δε-heterodimer is an unstable intermediate. (**B**–**F**) Protein abundances in mitoplasts from HEK293-Δα, -Δβ, -Δγ, -Δδ, and -Δε cells, respectively, determined by SILAC-MS, relative to HEK293-WT cells. Analyses were performed twice (biological replicates) with reciprocal SILAC labeling. The histograms represent the normalized abundances (% subunit abundance, *n* = 2) and show the two experimental values (gray circles) of the subunits of h-ATP synthase, of IF_1_, and of ATPAF1, ATPAF2, and FMC1. For the definition of IF_1_-M1, IF_1_-M3, f-1, and f-2, see legend to Fig. 2. γ-1 and γ-2 are isoforms of the γ-subunit (UniProt P36542). (**G**) SDS-PAGE analysis of the tagged γ-subunit (γ-t) or the tagged c-subunit (c-t) and associated proteins purified from DDM extracts of mitochondria from HEK293-∆β cells, where either γ-t (Δβ γ-t) or c-t (Δβ c-t) had been expressed. WT, DDM extract of mitochondria from HEK293-WT cells. Proteins were detected by western blotting with the antibodies to proteins indicated on the left. The band above the δ-subunit arises from a non-specific interaction with the antibody. (**H**) Proteins associated with γ-t in HEK293-∆β cells, determined by SILAC-MS. The points on the scatter plot represent the log base 2 values of the protein ratios from two complementary MS analyses (biological replicates), normalized by centering on the median protein ratio. Upper right quadrant, proteins associated with γ-t; intersection of the axes, non-specifically bound proteins common to both experiments (γ-t and CSKMT-t); bottom left quadrant, CSKMT-t. Tagged proteins are shown as black squares, ATP synthase subunits and assembly factors as blue circles and blue squares respectively, and all other identified proteins as gray circles. Protein ratios were derived from a minimum of two peptide ratios, except for ε-, e-, g-, and k-subunits in experiment 2, where the values were derived from a single peptide ratio. One contaminant data point has been excluded from the upper left quadrant (S100A7, ratio -4.31:3.75). Source data are available online for this figure.

**Figure EV3:**
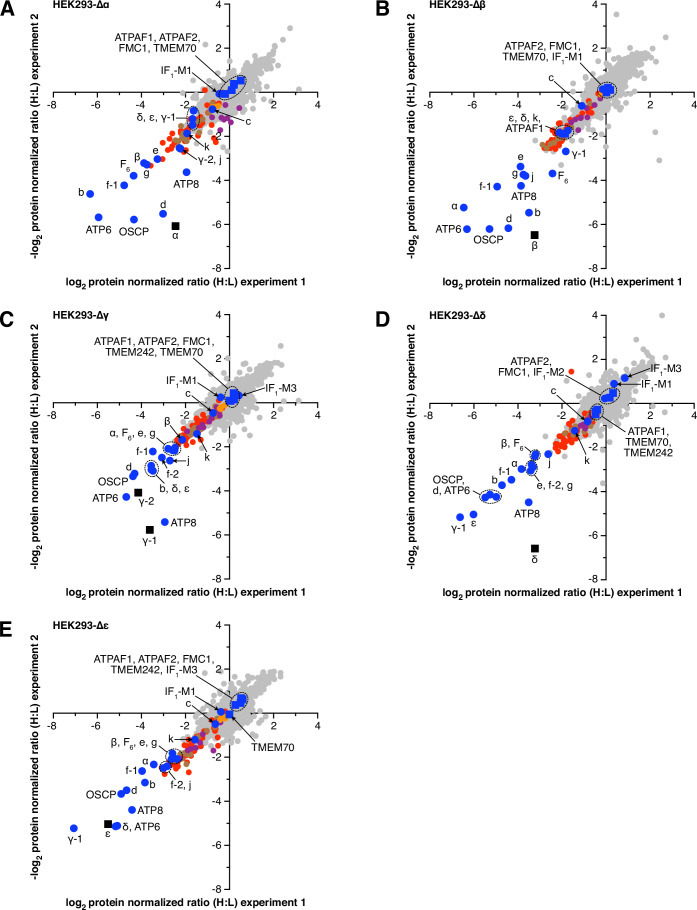
Quantitative impact of individual deletion of subunits of the F_1_-module of h-ATP synthase in HEK293 cells. Shown are protein abundances in mitoplasts from cells lacking each of the five subunits determined by SILAC-MS analysis relative to HEK293-WT cells. (**A**–**E**) HEK293-Δα, -Δβ, -Δγ, -Δδ, and -Δε cells, respectively. Black square, the deleted subunit; blue circles, subunits of ATP synthase; blue squares, assembly factors for ATP synthase; red circles, complex I subunits; orange circles, complex II subunits; purple circles, complex III subunits; brown circles, complex IV subunits; gray circles, all other identified proteins. The experiments were performed twice (biological replicates) with reciprocal SILAC labeling. The points on the scatter plot represent the log base 2 values of normalized protein ratios from the two complementary MS analyses. For the definition of IF_1_-M1, IF_1_-M3, f-1, and f-2, see legend to Fig. EV2. γ-1 and γ-2 are isoforms of the γ-subunit (UniProt P36542). Ratios for two non-interacting proteins PLXNA3 (4.37, 0.411) and ANP32B (5.01, −0.701) fall outside of the axes in (**A**, **C**), respectively.

To determine whether the α- and β-subunits are required for the assembly of the CS, their genes, plus those for the CS subunits, were disrupted individually in HEK293-WT cells (Appendix Figs. S3 and S4), producing HEK293-Δα, -Δβ, -Δγ, -Δδ, and -Δε cells, respectively. The absence of each deleted subunit was confirmed, and shown to diminish other ATP synthase components, but not the F_1_ AFs, ATPAF1, ATPF2, and FMC1, except for ATPAF1 in HEK293-Δβ cells (Fig. 3A). In the absence of either the α- or β-subunit, all four subunits of the rotor were made, at 22-60% abundance relative to WT cells (Fig. 3B,C). The levels of most other subunits of the enzyme were reduced to an average of 2–13% of WT levels, except for subunit k, which was reduced to 26% of the WT level (Figs. 3 and EV3). These observations indicate (but do not prove) that a CS-domain, and possibly an intact rotor (Fig. 1C), can be assembled in the absence of either the α- or the β-subunit. However, in the absence of any one of the three components of the CS, subunits γ, δ, and ε, the abundances of the two other components were also severely impacted, indicating a lack of either the CS or the rotor complex. In contrast, the level of the c-subunit was sustained in all cell lines. The residual levels of δ- and ε-subunits in ∆γ cells were 10% (Fig. 3D), twofold to fourfold higher than the levels of either in the absence of the other (Fig. 3E,F), suggesting a potential mutual protective effect.

**Figure EV4:**
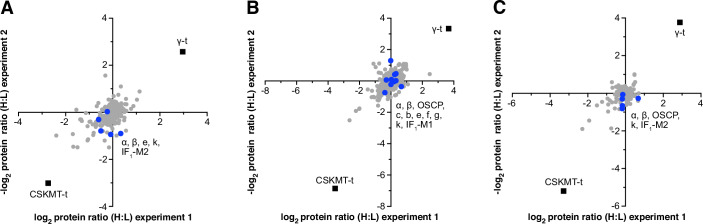
Failure of the γ-subunit of h-ATP synthase to interact with the α- or β-subunits in the absence of either the δ- or the ε-subunit. Relative abundances determined by quantitative MS of proteins associated with γ-t in SILAC-labeled cells. (**A**) HEK293-Δε cells and in (**B**) and (**C**), HEK293-Δδ cells. The mitochondrial protein CSKMT was expressed as the tagged reciprocal control protein. In (**C**), but not in (**B**), the buffer for solubilization and affinity purification of the proteins contained 1 mM MgCl_2_ and 0.2 mM ADP, and the procedures performed at 20 °C rather than 4 °C (as in **B**). The points on the scatter plot represent the log base 2 values of the protein ratios from two complementary MS analyses (biological replicates), normalized by centering on the median protein ratio. Any proteins specifically associated with γ-t would be in the upper right quadrant with γ-t. Non-specifically associated proteins common to both experiments (γ-t and CSKMT-t) are found near the intersection of the axes. CSKMT-t is in the bottom left quadrant. Black squares, tagged proteins; blue circles, ATP synthase subunits; gray circles, all other identified proteins. IF_1_-M2 is a specific mature form of IF_1_ (He et al, 2017b).

To investigate whether a rotor complex could be formed in the absence of the β-subunit, first it was confirmed that subunits γ and c, each with tandem C-terminal Strep II and FLAG tags (denoted as γ-t and c-t, respectively), were incorporated into the ATP synthase in HEK293-WT cells (Appendix Fig. S5 and Table S2) (Carroll et al, 2021). Then, γ-t and c-t were expressed in HEK293-Δβ cells, and sub-complexes containing them were purified. Both tagged subunits were associated with the other components of the rotor (Fig. 3G), in the case of γ-t confirmed by stable isotope labeling with amino acids in cell culture combined with mass spectrometric analysis (SILAC-MS) (Fig. 3H). Here, the relative enrichment of subunit c, although significant, was lower than that of the γ-, δ-, and ε-subunits, consistent with the presence of a CS complex and a complete CS-c_8_ rotor. The material purified in association with c-t appears to be a mixture of the rotor complex, the free c_8_-ring, and possibly smaller oligomeric forms of subunit c in association with AFs for the c_8_-ring, TMEM70, and TMEM242 (Kovalčíková et al, 2019; Carroll et al, 2021). None of the known AFs for the F_1_-module of ATP synthase was associated with either γ-t or c-t (Appendix Fig. S6). Also, in the absence of either the δ- or the ε-subunit, γ-t was not associated with either the α-subunit or the β-subunit (Fig. EV4), and so the formation of an F_1_-module appears to require a complete CS complex.

### The δε-complex

**Figure 4:**
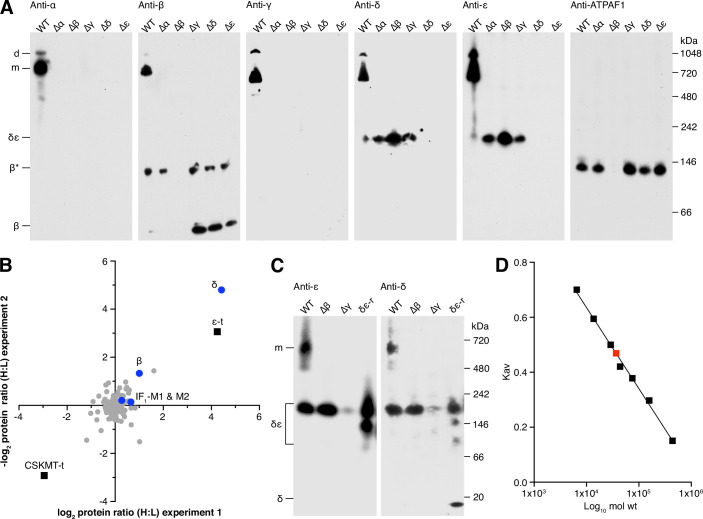
Assembly complexes formed following deletion of individual subunits of the F_1_-module of h-ATP synthase. (**A**) CN-PAGE analysis of digitonin extracts of mitoplasts (detergent:protein, 10:1, by wt) from HEK293-WT, -Δα, -Δβ, -Δγ, -Δδ, and -Δε cells. Proteins and complexes were detected with antibodies against the α-, β-, γ-, δ-, and ε-subunits of h-ATP synthase, and ATPAF1; d and m, dimeric and monomeric h-ATP synthase, respectively; δε, δε-complex; β*, complex of β-subunits and ATPAF1; β, β-subunit. The positions of molecular weight protein markers are shown on the right. (**B**) Specific binding of the δ-subunit to the tagged ε-subunit, ε-t. Shown are relative protein abundances determined by SILAC-MS of HEK293-Δγ cells where ε-t was expressed. A tagged version of the mitochondrial protein citrate synthase-lysine-methyltransferase (CSKMT-t) was expressed in the same cells as the reciprocal control. The points on the scatter plot represent the log base 2 values of the protein ratios from two complementary MS analyses (biological replicates), normalized by centering on the median protein ratio. Upper right quadrant, proteins associated with ε-t; intersection of the axes, non-specifically bound proteins common to both experiments (ε-t and CSKMT-t); bottom left quadrant, CSKMT-t. Tagged proteins are shown as black squares, ATP synthase subunits as blue circles, and all other identified proteins as gray circles. For a definition of IF_1_-M1 and -M2, see legend to Fig. 2. (**C**) CN-PAGE analysis of purified recombinant δε-complex (δε-r) (Appendix Fig. S8) compared with δε-complexes in digitonin extracts of mitoplasts from HEK293-WT, -Δβ, and -Δγ cells, detected by western blotting with antibodies against the ε- and δ-subunits (left and right panels respectively); m, monomeric ATP synthase. The positions of protein molecular weight standards are shown on the right. (**D**) Molecular weight estimation of recombinant δε complex by size-exclusion chromatography on a Superdex 200 Increase 10/300 GL column. Purified recombinant δε-r is the red square with a calculated mass of 36.8 kDa. Protein standards are shown as black squares. Source data are available online for this figure.

Detergent extracts of mitoplasts from HEK293-Δα, -Δβ, and -Δγ cells, and also from HEK293-WT cells, contained a novel complex with an apparent molecular weight of about 200 kDa (Fig. 4A), made only of the δ- and ε-subunits (molecular weights 15.0 and 5.6 kDa, respectively), but the complex was not formed in HEK293-Δδ or -Δε cells. In further investigation of the 200 kDa complex, first, it was confirmed that δ- and ε-subunits with C-terminal tandem Strep II and FLAG tags (δ-t and ε-t) were incorporated into the ATP synthase complex (Appendix Fig. S5). When δ-t was expressed in HEK293-Δ(c+OSCP) cells, two complexes were affinity-purified, the F_1_-module and the δε complex (Appendix Fig. S7 and Appendix Tables S3 and S4). When ε-t was expressed in HEK293-Δγ cells, the affinity-purified complexes contained only the δ- and ε-subunits (Fig. 4B). Therefore, the novel high molecular weight band represents an oligomer of the δε-complex. Thus, the independent assembly of the monomeric δε-heterodimer shows that its formation precedes the incorporation of the γ-subunit into the CS.

A recombinant form of the δε-complex (Appendix Fig. S8 and Appendix Table S4) behaved in a similar way to the natural oligomer, migrating on a native gel as two oligomers, one to the same position as the natural oligomer (Fig. 4C). However, in the absence of detergents it behaved as a (δε)_2_-dimer (Fig. 4D).

### Incorporation of the α- and β-subunits

When the γ-subunit was deleted in HEK293 cells, there was no evidence for the accumulation of an α_3_β_3_-subcomplex (Fig. 4A). Also, no complex containing only the α-, β-, and γ-subunits was identified when γ-t was expressed and purified in the absence of the δ or ε subunits (Fig. EV4). Therefore, the incorporation of the α- and β-subunits, and the formation of the F_1_-domain, appear to require the prior formation of the CS.

**Figure 5:**
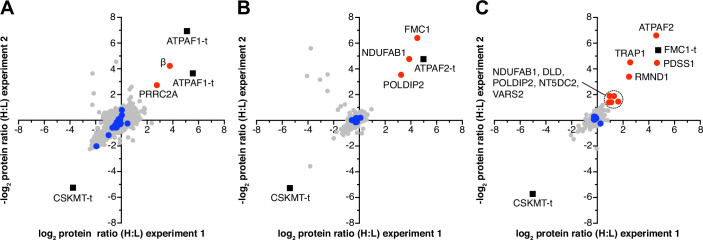
Proteins associated with assembly factors for the F_1_-module of h-ATP synthase. ATPAF1, ATPAF2, and FMC1, and a control protein CSKMT, with C-terminal tandem FLAG-Strep II tags, were overexpressed separately in HEK293-WT cells grown in SILAC media. Shown are relative abundances of tagged proteins and binding partners determined by SILAC-MS. The points on the scatter plot represent the log base 2 values of normalized protein ratios from the two complementary MS analyses (biological replicates). (**A**) ATPAF1-t and CSKMT-t. Extracts of cells expressing ATPAF1-t and CSKMT-t were purified separately by affinity chromatography, then combined and fractionated by SDS-PAGE. The two values for ATPAF1 correspond to the full-length form (isoform 1; Q5TC12-1) and a truncated form arising from translational initiation from an internal methionine codon (isoform 2; Q5TC12-2). (**B**) ATPAF2-t and CSKMT-t. Equal amounts of heavy or light labeled expression cells, and oppositely labeled cells expressing CSKMT-t, were combined, and proteins extracted from mitoplasts with digitonin (detergent:protein, 12:1, g:g). Tagged proteins and associated partners were purified by affinity chromatography on Strep-Tactin agarose and fractionated by SDS-PAGE. The experiment was performed twice with reciprocal SILAC labeling. (**C**) FMC1-t and CSKMT-t. Samples were processed as in (**B**). Each data point corresponds to the relative abundance ratio of an identified protein from the two complementary MS analyses. Tagged assembly factor(s), or control CSKMT, are shown as black squares. Non-associated ATP synthase subunits and assembly factors are blue circles, with proteins significantly associated with a tagged ATP synthase assembly factor(s) shown as red circles. All other proteins are represented by gray circles. Source data are available online for this figure.

Three AFs, ATPAF1, ATPAF2, and FMC1, are known to be required for the assembly of the h-F_1_-domain, (Wang et al, 2001; Li et al, 2017), but where they intervene in the assembly process has not been established. Here, a β:ATPAF1 complex was observed in HEK293-Δα, -Δγ, -Δδ, and -Δε cells, and also in HEK293-WT cells, but not (as expected) in HEK293-Δβ cells (Fig. 4A). It is notable that, in the absence of the β-subunit, the relative abundance of ATPAF1 was significantly lower than in the other cells (Fig. 3C). Further evidence for the association of the β-subunit with ATPAF1 was provided by introducing an expression plasmid encoding ATPAF1 with C-terminal tandem Strep II and FLAG tags (ATPAF1-t) into HEK293-WT cells. Again, the β-subunit and ATPAF1 were associated (Fig. 5A). In similar experiments with tagged ATPAF2 (ATPAF2-t) and FMC1 (FMC1-t) (Fig. 5B,C), ATPAF2-t was associated with FMC1 (and vice versa). In addition, NDUFAB1 (the mitochondrial acyl carrier protein and a subunit of complex I) (Runswick et al, 1991), POLDIP2 (polymerase δ-interacting protein 2), and other proteins were associated with both AFs. POLDIP2 and NT5DC2 (5’-nucleotidase domain-containing protein 2) have been observed previously in association with FMC1 (Floyd et al, 2016), and POLDIP2 has been proposed to interact with mitochondrial CLPX, an ATP-dependent ClpX-like chaperone component of the ClpXP protein degradation complex involved in mitochondrial proteostasis (Strack et al, 2020). The nature and functional significance of POLDIP2, and other subsidiary interactions with FMC1 (Fig. 5C), remain to be investigated.

However, for the current work, the important significance of these experiments is that they provided no evidence of any other binary complex between an F_1_-subunit and either ATPAF2 or FMC1. Therefore, ATPAF1 appears to facilitate the assembly of the β-subunit into the α_3_β_3_-domain part of the F_1_-domain, but the introduction of α-subunits, which are closely related to β-subunits (Appendix Fig. S9), occurs by a different and as yet incompletely understood mechanism, possibly involving AFs ATPAF2, FMC1, and NDUFAB1 (see below), but not involving the formation of a binary α:AF complex.

### Roles of assembly factors ATPAF1, ATPAF2, and FMC1

**Figure 6:**
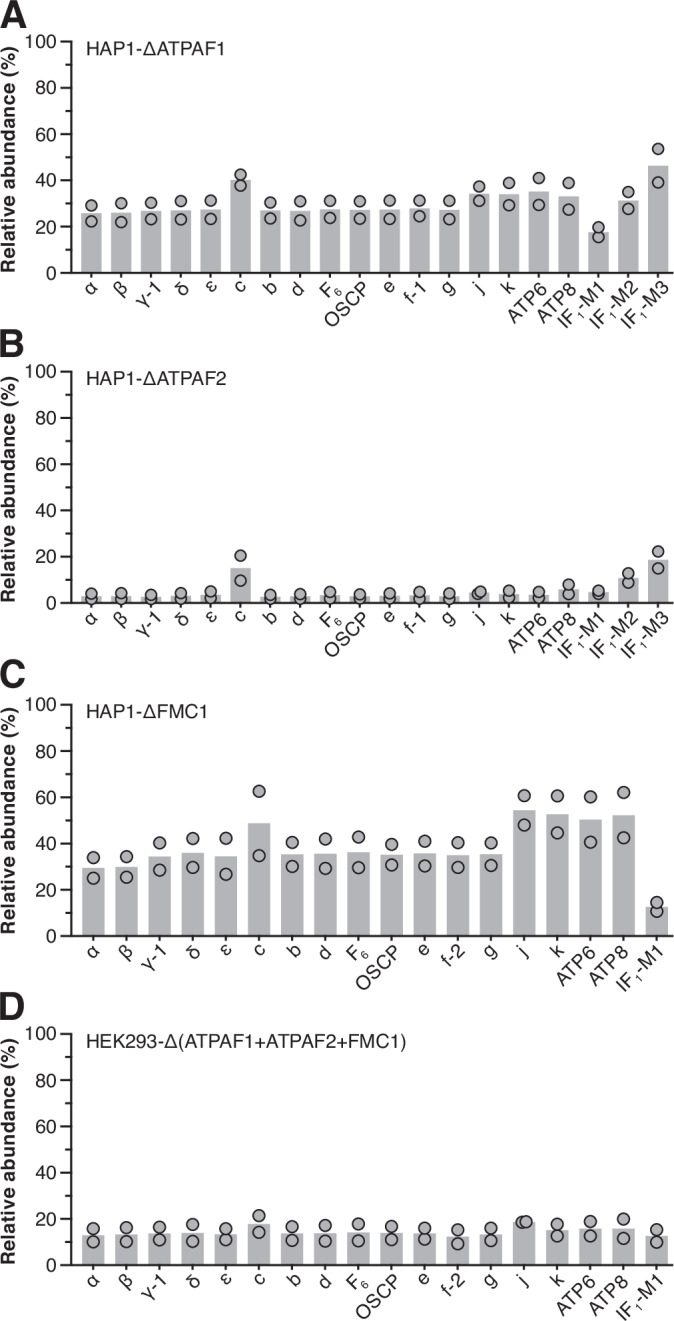
Assembly of human ATP synthase in the absence of assembly factors for the F_1_-module. Protein abundances of subunits of ATP synthase immunopurified from mitoplasts were determined by SILAC-MS relative to WT cells. The experiment was performed twice (biological replicates) with reciprocal SILAC labeling. Shown are average % subunit abundances (*n* = 2) and the two experimental values of the subunits (gray circles) in the following cell lines. (**A**) HAP1-ΔATPAF1. (**B**) HAP1-ΔATPAF2. (**C**) HAP1-ΔFMC1. (**D**) HEK293-Δ(ATPAF1 + ATPAF2 + FMC1). Ratios were determined from a minimum of two peptide values, except for the c-subunit in (**B**) where a single value was obtained in one of the two analyses. For definition of IF_1_-M1, IF_1_-M3, γ-1, γ-2, f-1, and f-2, see legends to Figs. 2 and 3. Source data are available online for this figure.

**Figure EV5:**
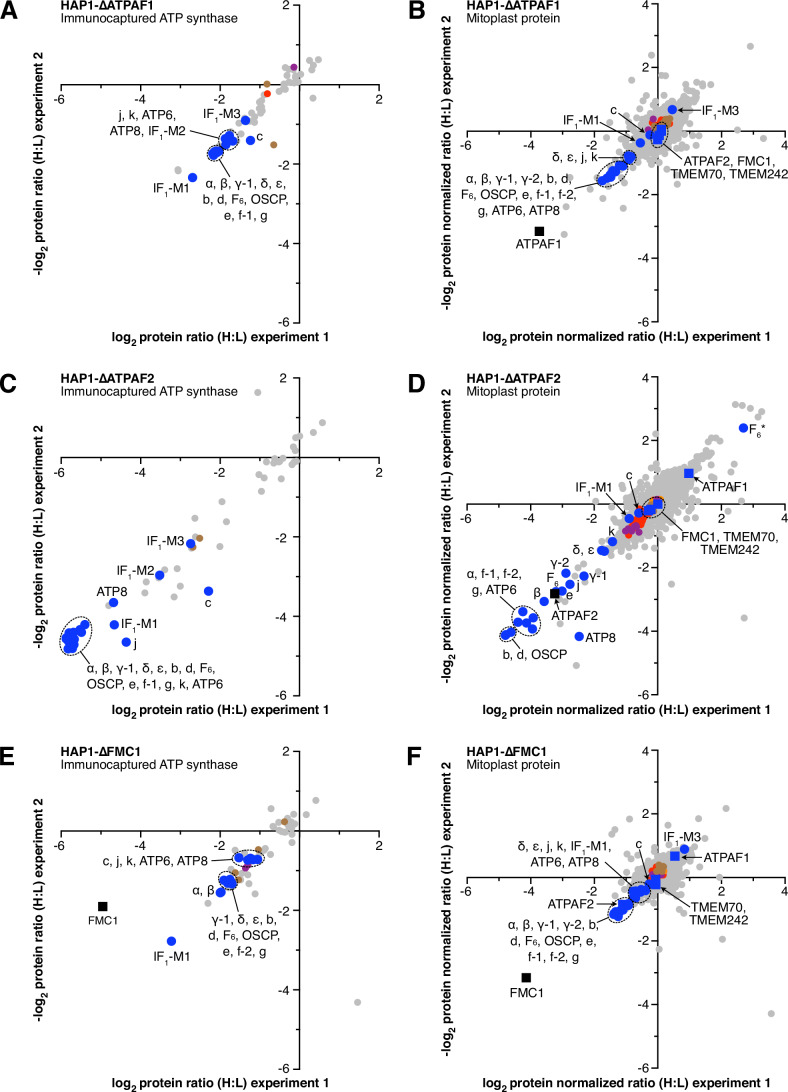
Impact of individual deletion of assembly factors for the F_1_-module of h-ATP synthase on the abundances of subunits of the enzyme and of the assembly factors. (**A**, **C**, **E**) Protein abundances in samples of immunocaptured ATP synthase or (**B**, **D**, **F**) in mitoplasts from HAP1 cells lacking each of the F_1_-domain assembly factors, determined by SILAC-MS analysis relative to HAP1-WT cells. (**A**, **B**) HAP1-ΔATPAF1 cells. (**C**, **D**) HAP1-ΔATPAF2 cells. (**E**, **F**) HAP1-ΔFMC1 cells. Black square, deleted protein; blue circles, subunits of ATP synthase; blue squares, assembly factors for ATP synthase; red circles, complex I subunits; orange circles, complex II subunits; purple circles, complex III subunits; brown circles, complex IV subunits; gray circles, all other identified proteins. The experiments were performed twice (biological replicates) with reciprocal SILAC labeling. The points on the scatter plot represent the log base 2 values of raw (**A**, **C**, **E**) or normalized (**B**, **D**, **F**) protein ratios from the two complementary MS analyses. For definition of IF_1_-M1, IF_1_-M3, γ-1, γ-2, f-1, and f-2, see legend to Figs. EV2 and EV3. (**D**) The ratios for F_6_ and F_6_* correspond to the mature and precursor forms of the subunit, which migrated to different positions in the SDS-PAGE gel, and were manually calculated using the peptide ratio output from the MaxQuant program.

**Figure EV6:**
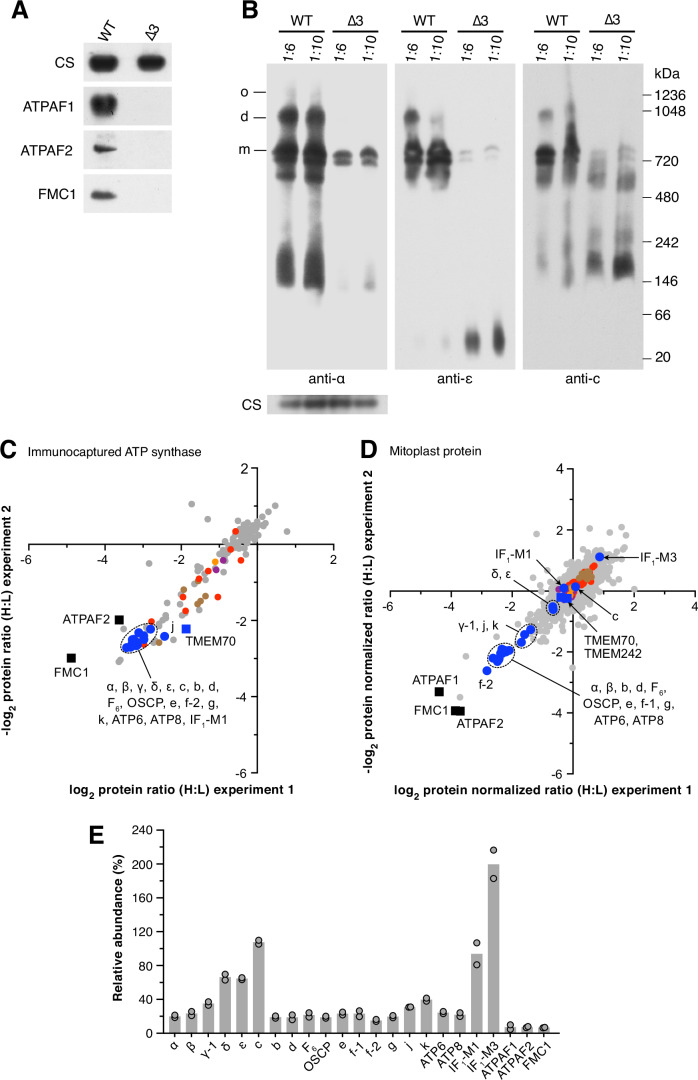
Characterization of HEK293-Δ(ATPAF1 + ATPAF2 + FMC1) cells and the impact on assembly of h-ATP synthase. (**A**) Western blots of SDS-PAGE analyses of DDM extracts of HEK293-WT and -Δ(ATPAF1 + ATPAF2 + FMC1) cells (denoted as Δ3). The specificities of the antibodies are indicated on the left. (**B**) Western blots of BN-PAGE analyses of digitonin extracts (6 g/g or 10 g/g protein) of HEK293-WT and -Δ(ATPAF1 + ATPAF2 + ATPAF3) cells. The specificity of the antibody is given beneath each panel. CS, citrate synthase, provided a loading control; o, d, and m, denote oligomeric, dimeric, and monomeric forms of ATP synthase, respectively. (**C**, **D**) Protein abundances in immunopurified ATP synthase and mitoplasts, respectively, from HEK293-Δ(ATPAF1 + ATPAF2 + FMC1) cells relative to HEK293-WT cells determined by SILAC and MS analysis. The experiment was performed twice with reciprocal SILAC labeling. The points on the scatter plots represent the log base 2 values of protein ratios from the two complementary MS analyses (biological replicates), with normalized values shown in (**D**). Black squares, the deleted assembly factors; blue circles, subunits of ATP synthase; blue squares, assembly factors for ATP synthase; red circles, complex I subunits; orange circles, complex II subunits; purple circles, complex III subunits; brown circles, complex IV subunits; gray circles, all other identified proteins. (**E**) Normalized average subunit abundances (%, *n* = 2) in mitoplasts of the two experimental values (gray circles) for ATP synthase subunits, the inhibitor protein IF_1_, and assembly factors ATPAF1, ATPAF2, and FMC1. For definition of IF_1_-M1, IF_1_-M3, γ-1, f-1 and f-2, see legend to Figs. EV2 and EV3.

The roles of the three AFs in facilitating the construction of ATP synthase were investigated by deletion of each of them individually from HAP1 cells leading to HAP1-ΔATPAF1, -ΔATPAF2, and -ΔFMC1 cells (Figs. 6A–C and EV5; Appendix Figs. S10 and S11). Then, all three AFs were removed together from HEK293-WT cells leading to HEK293-Δ(ATPAF1 + ATPAF2 + FMC1) cells (Figs. 6D and EV6; Appendix Figs. S12 and S13).

**Figure EV7:**
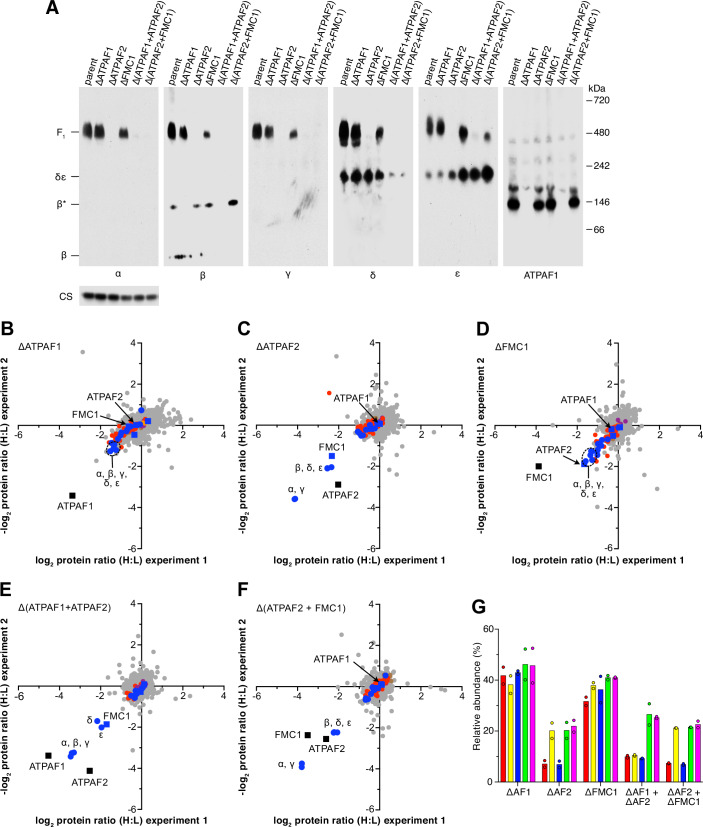
Effect of the deletion of the assembly factors for the F_1_-module of h-ATP synthase from HEK293-Δ(c + OSCP) cells. (**A**) The assembly factors were deleted singly from HEK293-Δ(c+OSCP) cells (or “parent”), denoted above as ΔATPAF1, ΔATPAF2, and ΔFMC1. The pairwise combinations of ATPAF1 and ATPAF2 and ATPAF2 and FMC1 in HEK293-Δ(c+OSCP) cells are denoted as Δ(ATPAF1 + ΔATPAF2) and Δ(ATPAF2 + ΔFMC1), respectively. Shown are western blots of CN-PAGE fractionated extracts of mitoplasts from HEK293-Δ(c+OSCP) and derivative cells prepared with digitonin (10:1, g:g of protein), probed with antibodies with the specificities denoted beneath each panel. On the left, F_1_, F_1_-domain, δε, the δε-complex, β*, complex of the β-subunit with ATPAF1, and β, the free β-subunit. The positions of protein molecular weight markers are shown on the right. Citrate synthase, CS, detected by western blotting of samples fractionated by SDS-PAGE, was employed as a loading control. (**B**–**F**) Protein abundances were measured in mitoplasts from HEK293-Δ(c+OSCP) cells lacking the assembly factors, relative to HEK293-Δ(c+OSCP) cells, by SILAC-MS analysis. The experiments were performed twice (biological replicates) with reciprocal SILAC labeling. The scatter plot shows the relative protein abundances, represented as the log base 2 values of normalized protein ratios from the two complementary MS analyses. (**B**) HEK293-Δ(c+OSCP + ATPAF1), (**C**) HEK293-Δ(c+OSCP + ATPAF2), (**D**) HEK293-Δ(c+OSCP + FMC1), (**E**) HEK293-Δ(c+OSCP + ATPAF1 + ATPAF2), (**F**) HEK293-Δ(c+OSCP + ATPAF2 + FMC1) cells, respectively. Black square, the deleted protein; blue circles, subunits of ATP synthase; blue squares, assembly factors for ATP synthase; red circles, complex I subunits; orange circles, complex II subunits; purple circles, complex III subunits; brown circles, complex IV subunits; gray circles, all other identified proteins. (**G**) Relative abundances (average % subunit abundance, *n* = 2) of the two experimental values (colored circles) of the subunits of the F_1_-domain in HEK293-Δ(c+OSCP) cells, where the assembly factors were removed singly and in combination: Red bar, α-subunit; yellow bar, β-subunit; blue bar, γ-subunit; green bar, δ-subunit; magenta bar, ε-subunit.

The deletion of ATPAF2 had the greatest impact on the assembly of ATP synthase, greatly diminishing the levels of all of the subunits of the enzyme, and reducing the abundance of the enzyme to 4% relative to WT cells (Fig. 6B). Deletion of either ATPAF1 or FMC1 had a milder effect, diminishing the relative abundances of the subunits to 30–40% (Fig. 6A,C). Notably, some intact ATP synthase (14% relative to WT) was still assembled in the absence of all three assembly factors in HEK293-Δ(ATPAF1 + ATPAF2 + FMC1) cells (Figs. 6D and EV6). In addition, each of the three AFs was deleted singly from HEK293-Δ(c+OSCP) cells that assemble the F_1_-module (Fig. 2), and in the pairwise combinations ATPAF1 with ATPAF2, and ATPAF2 with FMC1 (Appendix Figs. S14–17). It was found again that deletion of ATPAF2 had the greatest impact on the abundances of the five subunits of the F_1_-domain and on the assembly of the F_1_ domain (Fig. EV7). Also, in most instances, the removal of ATPAF2 led to a decrease of FMC1 (and vice versa), but had no impact on ATPAF1 (Figs. EV5 and EV7), suggesting that the formation of the ATPAF2:FMC1 complex protects its components.

ATPAF2 was reintroduced into both HEK293-Δ(c+OSCP + ATPAF1 + ATPAF2) cells (Appendix Fig. S18A), and HEK293-Δ(c+OSCP + ATPAF2 + FMC1) cells (Appendix Fig. S18C), leading an increase in the levels of α-, β-, γ-, and ε-subunits of the F_1_-domain in both cell lines, and an increase in the level of FMC1 in HEK293-Δ(c+OSCP + ATPAF1 + ATPAF2) cells. Recovery of subunit levels was observed even without the induction of expression of ATPAF2, probably because of “leaky” low-level expression of ATPAF2, but at levels sufficient to promote significant assembly. Similarly, reintroduction of ATPAF1 into HEK293-Δ(c+OSCP + ATPAF1 + ATPAF2) cells led to an increase in the level of the β-subunit only (Appendix Fig. S18B). In contrast, the reintroduction of FMC1 into HEK293-Δ(c+OSCP + ATPAF2 + FMC1) cells had no significant effect on the levels of subunits of the F_1_-domain (Appendix Fig. S18D), and the influence of the co-expression of ATPAF2 and FMC1 in HEK293-Δ(c+OSCP + ATPAF2 + FMC1) (Appendix Figs. S18E and S19) was similar to that of reintroducing ATPAF2 alone. These latter experiments support the conclusion that ATPAF2 is the dominant assembly factor for the F_1_-module, aided by FMC1, and that ATPAF1 modulates F_1_ assembly via a stable interaction with the β-subunit.

## Discussion

Here, first, we demonstrated that human cells can assemble subunits α, β, γ, δ, and ε of h-ATP synthase into an independent catalytic F_1_-module. Since the ATPase inhibitor protein IF_1_ is associated with this F_1_-module, it is competent in the hydrolysis of ATP, as the formation of the F_1_-IF_1_ inhibited complex requires the hydrolysis of two ATP molecules (Bason et al, 2014; Kobayashi et al, 2021).

Second, we showed that the CS and the c_8_-ring can assemble independently, and then, in a subsequent step, associate to form the CS-c_8_ rotor. Previously, we confirmed that TMEM70 participates in the assembly of the c_8_-ring (Kovalčíková et al, 2019; Carroll et al, 2021), and established that TMEM242 is also required (Carroll et al, 2021). Here, we have demonstrated that the CS is built by the association of the δ- and ε-subunits into a δε-complex, and the subsequent incorporation of the γ-subunit. A δε-subcomplex participates in the assembly of ATP synthase from *Arabidopsis thaliana* (Röhricht et al, 2021), and 270 kDa and 250 kDa complexes containing δ- and ε-subunits have been observed in mitochondrial extracts from HeLa cells (Fujikawa et al, 2015). We found that the native and recombinant δε-complexes migrated with an anomalously high molecular mass in CN-PAGE analyses, but in the absence of detergents, their behavior resembled a δε-dimer of dimers. Whether the self-association of the δε-subcomplex is a requirement for its incorporation into the CS remains unclear. In previous studies with either a recombinant or native δε-complex present in a 1:1 molar ratio, the molecular mass was larger than the calculated value, but below that of the (δε)_2_-dimer (Penin et al, 1990; Orriss et al, 1996).

**Figure 7:**
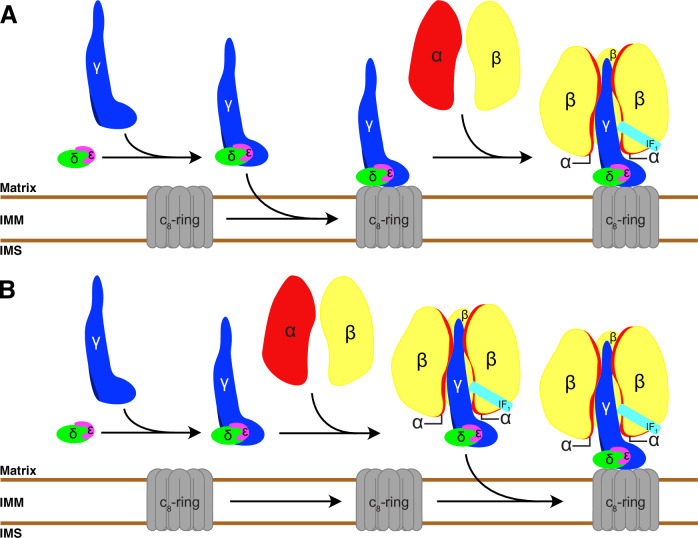
Co-existing alternative pathways for assembly of the rotor and incorporation of the central stalk into human ATP synthase. (**A**) A central stalk subcomplex is assembled from a preformed δε-subcomplex and a γ-subunit. Then the rotor is formed by association of the CS with a preformed c_8_-ring, assembled with the assistance of TMEM70 (Kovalčíková et al, 2019; Carroll et al, 2021) and TMEM242 (Carroll et al, 2021). Then the F_1_-module is built by the addition of the three α-subunits with the assistance of ATPAF2, FMC1, and NDUFAB1, and three β-subunits with the assistance of ATPAF1. (**B**) As in (**A**), a CS domain is assembled from a preformed δε-subcomplex and a γ-subunit. Then the three α- and β-subunits are introduced alternately around the preformed CS, forming the F_1_-domain, which then attaches to the c_8_-ring. (**A**, **B**) IF_1_ prevents the hydrolysis of ATP in intermediates with complete F_1_-modules. An α-subunit has been omitted from the α_3_β_3_ domains to expose the CS domain. Source data are available online for this figure.

At least three possible models can be envisaged for the formation of the F_1_-module. The first involves the building of an α_3_β_3_-domain around the coiled-coil region of the γ-subunit, and then completion of the CS by addition of the δε-complex. However, no evidence was found for the formation of an α_3_β_3_γ-complex. The second model is the prior formation of an α_3_β_3_-domain, and then entry of the upper part of the α-helical coiled-coil of the CS into the unoccupied central region of the α_3_β_3_-domain. However, no evidence was found for the formation of a separate α_3_β_3_-domain. The third model involves assembling the α_3_β_3_-domain around the coiled-coil region of the γ-subunit pre-incorporated into either a CS or a complete rotor. Current evidence strongly favors the two alternative routes of this third pathway (Fig. 7A,B), but whether either is preferred under specific circumstances is not known.

**Figure EV8:**
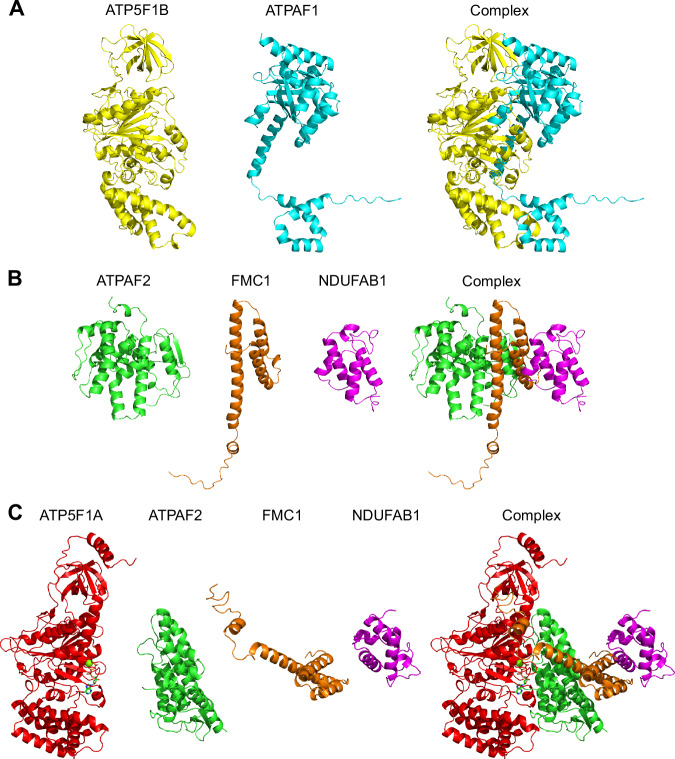
Structures of assembly complexes for the F_1_ module of h-ATP synthase. Model 0 structures predicted with AlphaFold 3 (Abramson et al, 2024) from the mature protein sequences are shown. ATP5F1B (β-subunit) (P06576; residues 47–529), ATPAF1 (Q5TC12, residues 52–328), ATPAF2 (Q8N5M1, residues 43–289), FMC1 (Q96HJ9; residues 2–113), NDUFAB1 (O14561; residues 69–156) lacking a 4’-phosphopantetheine-acyl chain modification on Ser-112 (Ser-44 of the mature protein) and ATP5F1A (α-subunit) (P25705; residues 44–553). (**A**) ATP5F1B and ATPAF1 proteins and complex (ipTM 0.78, pTM 0.73, ranking score 0.81). (**B**) ATPAF2, FMC1, and NDUFAB1 proteins and complex (ipTM 0.89, pTM 0.86, ranking score 0.92). (**C**) ATP5F1A, ATPAF2, FMC1, and NDUFAB1 proteins and complex (ipTM 0.87, pTM 0.88, ranking score 0.89). The Mg^2+^ ion and ATP were also added, and incorporated into the ATP5F1A model.

**Figure EV9:**
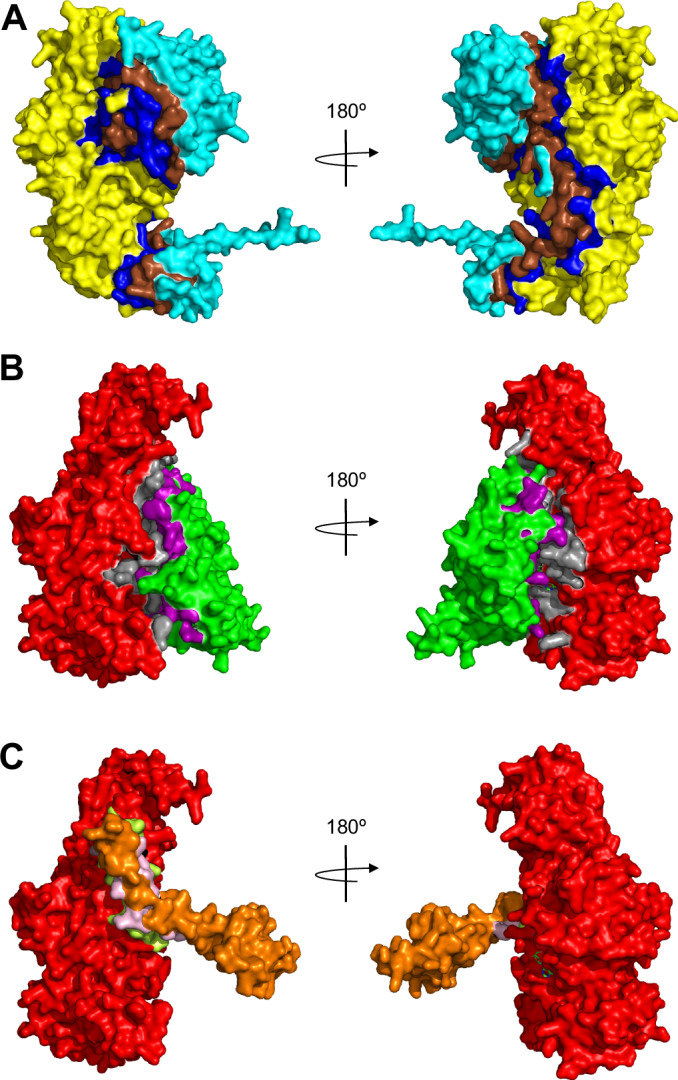
Interactions of the α- and β-subunits with assembly factors. The structures were predicted with AlphaFold 3, and modeled in surface representation with PyMol with the assistance of the script InterfaceResidues for depicting interface residues. (**A**) β-ATPAF1 complex. The body of the β-subunit is yellow, with residues in the interface with ATPAF1 in blue. The body of ATPAF1 is cyan with interface residues in brown. (**B**) Interaction of the α-subunit with FMC1 in the α-ATPAF2-FMC1-NDUFAB1 complex. The body of the α-subunit is red with residues in the interface with ATPAF1 in gray. The body of ATPAF2 is green with the interacting residues in purple. (**C**) Interaction of the α-subunit with FMC1 in the α-ATPAF2-FMC1-NDUFAB1 complex. The body of the α-subunit is red with residues in the interface with FMC1 in lime. The body of FMC1 is orange, and interacting residues are pink.

How the construction of the α_3_β_3_-domain around a preformed CS is achieved in human cells, and the precise molecular roles of ATPAF1, ATPAF2, and FMC1 in this process, remain to be specified, but they appear to play similar roles to their yeast orthologs (Lefebvre-Legendre et al, 2001; Ackerman, 2002; Li et al, 2017). As in *S. cerevisiae*, human ATPAF1 forms a stable binary complex with a β-subunit (Figs. 4A and 5A). However, despite their structural relatedness, the modes of incorporation of the α- and β-subunits are not equivalent, as a comparable complex is not formed between an AF and the α-subunit. In a model of the interaction between a β-subunit and ATPAF1 (Fig. EV8A; Appendix Fig. S20), the AF is bound extensively to all three domains of the β-subunit (Fig. EV9A; Appendix Table S5). However, the β-subunit is not folded fully, and so the final structure may be attained during its incorporation into the F_1_-domain (Appendix Fig. S21). The position of ATPAF1 in this complex approximates to that of the α-helical region of γ-subunit in the intact F_1_-domain, with the linker region between the N- and C- terminal domains of ATPAF1 interacting with the nucleotide binding site. Thus, the γ-subunit may displace ATPAF1 during the assembly process, as proposed before in *S. cerevisiae* (Ludlam et al, 2009).

ATPAF2 is the dominant AF in the incorporation of the α- and β-subunits into the F_1_-module (Figs. 6, EV5, and EV7). This dominance is supported by the observation that ATPAF2 knockout in mice is embryonic lethal, whereas ATPAF1 knockout mice are viable (Zhou et al, 2021). ATPAF2 and FMC1 have been reported to interact previously (Li et al, 2017). FMC1 is one of twelve members of the LYRM protein family, and their function requires them to interact with NDUFAB1 (Angerer, 2015; Brown et al, 2017; Van Vranken et al, 2018; Majmudar et al, 2019). Both FMC1 and ATPAF2 are amongst numerous binding partners of NDUFAB1 (Majmudar et al, 2019; Dibley et al, 2020), inferring that a ternary FMC1:ATPAF2:NDUFAB1 complex may form. Here, it is shown that ATPAF2 forms a ternary complex with FMC1 and NDUFAB1 (Figs. 5B,C and EV8B) and also a plausible quaternary complex with an α-subunit (Fig. EV8C). Thus, the ternary complex could participate in the introduction of α-subunits into the F_1_-domain. However, as the region of its interaction with the α-subunit is approximately where the α-subunit interacts with the β-subunit in the intact enzyme, it differs from the region of interaction of ATPAF1 with the β-subunit. Both ATPAF2 and FMC1 are predicted to interact with the α-subunit, but the majority of the interaction is with ATPAF2 (Fig. EV9B,C; Appendix Table S5). The introduction and folding of α-subunits also require the insertion of ATP or ADP into the nucleotide-binding site of each of them. Once inserted, these nucleotides remain bound permanently and do not exchange during catalysis (Abrahams et al, 1994).

A number of general comments can be made about the assembly of h-ATP synthase and the roles of AFs. First, the AFs are required for building oligomeric rings; TMEM70 and TMEM242 for the construction of the c_8_-ring and ATPAF1, ATPAF2, and FMC1 for fashioning the α_3_β_3_-domain. In contrast, other subunits assemble appropriately into the intermediate complexes, sculpting the structurally diverse features of the enzyme without participation of AFs. Second, the assembly process avoids building incomplete and uncoupled intermediate ATP synthase precursors that could wastefully either hydrolyze ATP or dissipate the proton-motive force. Thus, all of the characterized intermediates with an intact F_1_-module capable of ATP hydrolysis but lacking proton half-channels, and therefore incapable of ATP synthesis, are prevented from hydrolyzing ATP by the intervention of IF_1_, the unidirectional protein inhibitor of ATPase hydrolysis (Pullman and Monroy, 1963; Bason et al, 2014; Kobayashi et al, 2023; Carroll et al, 2024). Therefore, IF_1_ can be considered to be another assembly factor for ATP synthase. An equally important feature is that the proton half channels are created late in the assembly process by inserting ATP6 together with ATP8, when all other functional components of the rotor and stator are in place (He et al, 2018). The addition of subunit j completes the insertion process by apparently locking ATP6 in place (He et al, 2018). The assembly pathway of the ATP synthase in *S. cerevisiae*, where the c-subunit (Atp9) in addition to Atp6 and Atp8 is encoded in mitochondrial DNA (Tzagoloff and Myers, 1986), achieves the same end of creating the proton half-channels late in the assembly process in a related but non-identical way (Rak et al, 2011; Song et al, 2018). In the human enzyme, the final step in assembly is the addition of the k-subunit involved in linking dimeric complexes, possibly with subunit g, into the rows of dimeric ATP synthases observed on the tips of the cristae (He et al, 2018; Pinke et al, 2020; Spikes et al, 2021).

A number of issues in the assembly of h-ATP synthase remain to be resolved, including the following. First, the structures of the intermediate complexes described here and previously (Fujikawa et al, 2015; He et al, 2017a, 2017b, 2018, 2020), including the AF-complexes predicted with AlphaFold3 (Fig. EV8), and their exact role in assembling the α_3_β_3_-domain, require further experimental validation, for example by structural analysis by cryogenic electron microscopy (cryo-EM) (Nguyen et al, 2026). The capabilities of Alphafold3 (Abramson et al, 2024), employed here to examine interactions between α- and β-subunits and AFs (Fig. EV8), are significantly superior to earlier versions, insofar as they allow unknown structures of protein complexes, including bound small molecules, to be modeled with reasonable confidence. However, some limitations remain. For example, spurious structures can arise from intrinsically disordered regions such as the C-terminal 30 residues of FMC1 (Appendix Fig. S22). Also, the predictions are static and give no information about the dynamics of the proteins and the complexes that they form, whereas different conformational states can be trapped and their structures determined by cryo-EM. A second residual issue concerning the assembly of the h-ATP synthase is that where alternate pathways to intermediates exist, as proposed in Fig. 7, it is not known which one predominates, and whether the preferred pathway(s) is (are) influenced by physiological factors. A third remaining question about the assembly of the h-ATP synthase is how and when in the assembly process are the permanently bound specific phospholipids (Spikes et al, 2020) introduced into the wedge? A fourth question is where in the mitochondrion does assembly and insertion of ATP6 and ATP8 occur, at the cristae tips or elsewhere? A related issue is when, in the assembly process, does dimerization take place? The recent determination of high-resolution in organello structures of membrane-bound protein complexes by cryo-EM (Coupland et al, 2024; Zheng et al, 2024) and other advances in situ using cryo-electron tomography and subtomogram averaging (Dietrich et al, 2024) may provide the means to elucidate some of these questions.

**Figure EV10:**
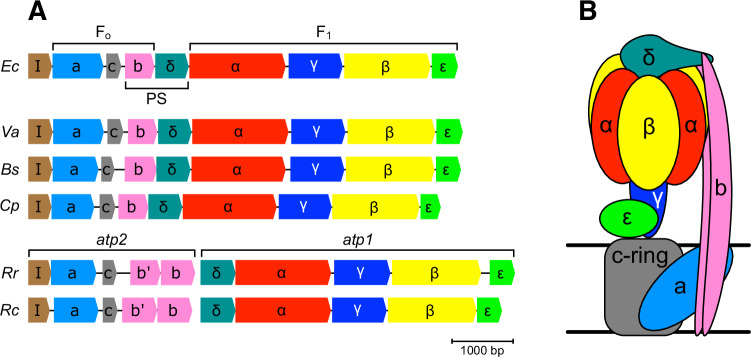
Organization of genes for subunits of various bacterial ATP synthases. Their subunit compositions are simpler than those of mitochondrial enzymes, but essential core features of the rotor and stator are retained. (**A**) Examples of *atp* operons from two Gram-negative (*Ec* and *Va*) (Walker et al, 1984; Krumholz et al, 1989), two Gram-positive (*Bs* and *Cp*) (Santana et al, 1994; Das and Ljungdahl, 2003) and two Gram-negative purple non-sulfur (*Rr* and *Rc*) (Falk et al, 1985; Falk and Walker, 1988; Borghese et al, 1998a, 1998b) bacteria. *Ec*, *Escherichia coli*. *Va*, *Vibrio alginolyticus*. *Bs*, *Bacillus subtilis*. *Cp*, *Clostridium pasteurianum*. *Rr*, *Rhodospirillum rubrum*. *Rc*, *Rhodobacter capsulatus*. The *Ec* operon is an example of a single operon archetypal arrangement. The genes are clustered into those encoding subunits of the F_o_, peripheral stalk (PS), and F_1_-modules. The promoter proximal genes (*uncI* or *atpI*) encode an integral membrane protein assembly factor for the c-ring module (Suzuki et al, 2007). The sizes of c-rings vary between species. In purple non-sulfur bacteria, the genes are organized into two separate operons, *atp1* encoding the F_1_-domain, and *atp2* encoding the F_o_-domain, with the PS genes straddling both operons. (**B**) Organization of the subunits of eubacterial ATP synthases. The δ- and ε-subunits are the equivalent subunits of human OSCP and δ, respectively (Walker et al, 1982). The bacterial PS consists of either two identical b-subunits or single copies of related b’- and b-subunits. Each has a single membrane spanning α-helix at its N-terminus.

A final point is that the assembly of the h-ATP synthase via the formation of the intermediate F_1_-module, the c_8_**-**ring and the rotor, and the PS-module adds further support to the proposal that the ATP synthase arose by the independent evolution of its F_1_ and F_o_ modules (Walker and Cozens, 1986). When the proposal was made, the PS-module had not yet been discovered, but following the discovery (Walker, 1998; Walker and Dickson, 2006), it was apparent that the gene orders were consistent with an independent evolution of all three modules (Fig. EV10). Also, the assembly pathways of F-ATP synthases in other species proceed in a modular fashion (Rak et al, 2011; Deckers-Hebestreit, 2013; Song et al, 2018), providing additional support to this facet of the evolution of this extraordinarily complex enzyme.

## Methods

**Table:** Reagents and tools table

Reagent/resource	Reference or source	Identifier or catalog number
**Experimental models**		
Flp-In^TM^ T-Rex^TM^ HEK293 cells	Thermo Fisher Scientific	Cat#R78007
HAP1-ΔATPAF1	Horizon Discovery	Cat#HZGHC004752c011
HAP1-ΔATPAF2	Horizon Discovery	Cat#HZGHC004753c011
HAP1-ΔFMC1	Horizon Discovery	Cat#HZGHC007013c009
*Escherichia coli* C41 (DE3)	Miroux and Walker, 1996	
**Recombinant DNA**		
pcDNA^TM^5/FRT/TO	Thermo Fisher Scientific	Cat#V652020
α-subunit human cDNA	Source Bioscience	IMAGE:3867375
β-subunit human cDNA	Source Bioscience	IMAGE:100009239
γ-subunit human cDNA	Source Bioscience	IMAGE:3445817
δ-subunit human cDNA	Source Bioscience	IMAGE:4798666
ε-subunit human cDNA	Source Bioscience	IMAGE:2988582
ATPAF1 human cDNA	Source Bioscience	IMAGE:4281102
ATPAF2 human cDNA	Source Bioscience	IMAGE:3836710
FMC1 human cDNA	Source Bioscience	IMAGE:4276817
pRK793 (His-TEV protease)	Addgene	Cat# 8827
δε-expression plasmid	This study	See Methods section
**Antibodies**		
Anti-ATP synthase α	Proteintech	Cat#14676-1-AP
Anti-ATP synthase β	Santa Cruz	Cat#sc-33618
Anti-ATP synthase γ	Proteintech	Cat#60284-1-Ig
Anti-ATP synthase δ	Abclonal	Cat#A9929
Anti-ATP synthase ε	Santa Cruz	Cat#sc-393695
Anti-ATP synthase b	Santa Cruz Abcam	Cat#sc-514419 Cat#ab217062
Anti-ATP synthase OSCP	Proteintech	Cat#66696-1-Ig
Anti-ATP synthase j	Merck Sigma	Cat#HPA058978
Anti-ATP synthase c	Abcam	Cat#ab180149 and ab181243
Anti-ATP synthase ATP8	Proteintech	Cat#26723-1-AP
Anti-ATP synthase IF_1_	In-house	Rabbit polyclonal against recombinant bovine protein
Anti-ATPAF1	Proteintech Santa Cruz	Cat#18016-1-AP Cat#sc-398684
Anti-ATPAF2	Merck Sigma	Cat#HPA023329
Anti-FMC1	Merck Sigma	Cat#HPA045663 and HPA050553
Anti-TMEM70	Santa Cruz	Cat#sc-393619
Anti-TMEM242	In-house	Rabbit polyclonal against recombinant human protein
Anti-citrate synthase	Proteintech	Cat#16131-1-AP
Anti-SDHA	Proteintech	Cat#14865-1-AP
Anti-TOM20	Santa Cruz	Cat#sc-11415
Anti-Strep-tag II	Abcam	Cat#Ab184224
Anti-rabbit HRP-conjugate	Thermo Fisher Scientific	Cat#31460
Anti-mouse HRP-conjugate	Thermo Fisher Scientific	Cat#A16066
**Oligonucleotides and other sequence-based reagents**		
gRNA primers	This study	Appendix Table S6
PCR primers	This study	Appendix Tables S7 and S8
**Chemicals, enzymes, and other reagents**		
Digitonin	Calbiochem	Cat#300410
n-dodecyl-β-D-maltoside	Glycon Biochemicals	Cat#D97002-C
NativePAGE™ 3-12% Bis-Tris gel	Invitrogen	Cat#BN1003BOX
Novex™ 10-20% Tris-Glycine gel	Invitrogen	Cat#XP10205BOX
Bolt™ Bis-Tris Plus 12% gel	Invitrogen	Cat#NW00127BOX
Immobilon-P	Millipore	Cat#IPVH00010
ECL Prime	Amersham	Cat#RPN2232
InstantBlue Coomassie stain	Abcam	Cat#ab119211
ATP Synthase Immunocapture Kit	Abcam	Cat#ab109715
Strep-Tactin agarose	IBA-Lifesciences	Cat#6-6350-010
D-desthiobiotin	Sigma-Aldrich	Cat#D1411
Trypsin	Roche	Cat#11418475001
Chymotrypsin	Roche	Cat#11418467001
DMEM	Gibco	Cat#31966-021
IMDM	Gibco	Cat#12440-053
DMEM for SILAC	Thermo Fisher Scientific	Cat#88364
IMDM for SILAC	Thermo Fisher Scientific	Cat#88367
L-Lysine	Sigma-Aldrich	Cat#L8662
L-Arginine-^13^C_6_,^15^N_4_ HCl	Sigma-Aldrich	Cat#608033
L-Arginine	Sigma-Aldrich	Cat#A8094
L-Lysine-^13^C_6_,^15^N_2_ HCl	Sigma-Aldrich	Cat#608041
L-Proline	Sigma-Aldrich	Cat#P5607
DTT	Melford	Cat#MB1015
TCEP	Sigma-Aldrich	Cat#646547
Iodoacetamide	Sigma-Aldrich	Cat#I1149
Zeocin	Thermo Fisher Scientific	Cat#R25001
Blasticidin	Thermo Fisher Scientific	Cat#A1113903
Hygromycin B	Thermo Fisher Scientific	Cat#10687010
Sodium pyruvate	Gibco	Cat#11360-039
Glutamax	Gibco	Cat#35050-038
Glucose	Gibco	Cat#A24940-01
Oligomycin	Sigma-Aldrich	Cat#75351
FCCP	Sigma-Aldrich	Cat#C2920
Rotenone	Sigma-Aldrich	Cat#R8875
Antimycin A	Sigma-Aldrich	Cat#A8674
Sulforhodamine B	Sigma-Aldrich	Cat#S1402
BCA protein assay kit	Pierce	Cat#23227
DC protein assay kit	BioRad	Cat#5000112
isopropyl β-D-1-thiogalactoside	Melford	Cat#MB1008
Myoglobin	Sigma-Aldrich	Cat#M1882
Trypsinogen	Sigma-Aldrich	Cat#T1143
Gel filtration calibrant kit(s)	Cytiva	Cat#28403841 and #28403842
**Software**		
MaxQuant version 2.0.1.0, with Andromeda	Cox and Mann, 2008, 2011	https://maxquant.org
Perseus	Tyanova et al, 2016	https://maxquant.org
PeakView 2.1	Sciex Analyst 1.4	https://sciex.com
PyMol 3.1.6.1	Schrödinger, LLC	https://pymol.org
InterfaceResidues.py		https://pymolwiki.org
AlphaFold_coloring.py		https://github.com/ailienamaggiolo/alphafold_coloring
Prism 10	Graphpad Software, LLC	https://graphpad.com
AlphaFold 3	Abramson et al, 2024	https://alphafoldserver.com
flDPnn	Hu et al, 2021	https://biomine2.cs.vcu.edu/servers/flDPnn/
CHOPCHOP	Labun et al, 2019	https://chopchop.cbu.uib.no
CRISPOR	Concordet and Haeussler, 2018	http://crispor.gi.ucsc.edu
Exon-Intron graphic maker		http://wormweb.org/exonintron
**Other**		

### General methods

Cell protein concentrations were determined by either the bicinchoninic acid assay (Thermo Fisher Scientific) or the detergent compatible (DC) protein assay (BioRad). Mitoplasts were prepared from cells with digitonin (Klement et al, 1995; Rhein et al, 2014). Extracts with n-dodecyl β-D maltoside (DDM; 1%, w/v) were fractionated by polyacrylamide gel electrophoresis in the presence of sodium dodecyl sulfate (SDS-PAGE), and proteins of interest were detected by western blotting. The oligomeric states of ATP synthase and vestigial complexes and of mitochondrial respiratory complexes in digitonin extracts of mitoplasts were examined by blue-native- (BN) or clear-native- (CN)-PAGE, respectively (Schägger and von Jagow, 1991; Wittig et al, 2007; He et al, 2020). Fractionated proteins in gels were transferred by electrophoresis to polyvinylidene fluoride membranes. Membranes were either probed with subunit-specific antibodies (see Reagents and Tools Table), or stained with Coomassie R250 dye. Excised stained protein bands were analyzed by N-terminal amino acid sequencing (Alta Bioscience Ltd). For the affinity purification of ATP synthase with an immunocapture resin (Abcam), and of Strep II tagged proteins with Strep-Tactin Sepharose (IBA Lifescience), see (He et al, 2012; Rhein et al, 2014; He et al, 2017b; Carroll et al, 2021). Except for cells where ATPAF1-t was expressed, equal protein quantities of SILAC-labeled samples were mixed, affinity-purified, reduced, and alkylated in gel sample buffer (Rhein et al, 2013), fractionated by SDS-PAGE, and proteins detected with Instant Blue Coomassie stain (Abcam). Proteins in excised stained gel sections were digested with trypsin or chymotrypsin (Wilm et al, 1996). Alternatively, affinity-purified protein samples were precipitated with ethanol at −20 °C for ≥18 h with 20 vol. of ethanol, centrifuged, and the pellet was digested in 50 mM ammonium bicarbonate for 18 h, with either trypsin at 37 °C or chymotrypsin at 30 °C, without reduction and alkylation. To avoid excess ATPAF1-t from binding to any free β-subunit in control cell extracts, cells expressing ATPF1-t were fractionated separately from controls expressing CSKMT-t. Then the samples were combined and analyzed as above. Proteins in stained gel bands were identified by tandem MS of multiply charged peptide ions in an Orbitrap Q-Exactive instrument, and comparison with a UniProt human protein sequence database with MASCOT (Perkins et al, 1999).

### Cell culture

HAP1 cells were cultured as described before (He et al, 2017b). Flp-In^TM^ T-Rex^TM^ HEK293 cells (Thermo Fisher Scientific; referred to as HEK293-WT cells) and derivative clonal cells were cultured in Dulbecco’s Modified Eagle Medium (DMEM) containing zeocin (100 μg/mL) and blasticidin (10 μg/mL), at 37 °C with an atmosphere of 5% CO_2_. For stable expression of proteins, HEK293-WT cells and derivatives were grown in DMEM, containing tetracycline-free fetal bovine serum (10%, v/v), hygromycin (100 μg/mL), and blasticidin (10 μg/mL). SILAC (Ong et al, 2002) of HAP1 and HEK293-WT cells, and of clonal cells containing disrupted genes, was carried out as described (Rhein et al, 2014; He et al, 2017b), with HEK293 cell media supplemented with 20 mM HEPES, pH 7.4. Cell stocks tested negative for mycoplasma.

### Disruption of genes

For the structures of *ATP5MC1*, *ATP5MC2*, *ATP5MC3*, *ATP5PO*, *ATP5F1A*, *ATP5F1B*, *ATP5F1C*, *ATP5F1D*, *ATPAF1*, *ATPAF2*, and *FMC1*, see Appendix Figs. S1, S3, and S12. For gRNAs employed in CRISPR disruptions (Ran et al, 2013) in HEK293-WT cells, see Appendix Table S6. Except for *ATP5F1D* where a single gRNA was employed, pairs of gRNAs were used in all HEK293-derived clones. HAP1-ΔATPAF1, -ΔATPAF2, and -ΔFMC1 cells, where each gene had been disrupted with a single gRNA (*ATPAF1*, AGTTTGATTTGATCTGGAAC; *ATPAF2*, CTGTCGGCGGGGCGTAAGCC; *FMC1*, TGAAGGCTTTCCGTGCACAT) were purchased from Horizon Discovery.

#### HEK293-Δα, -Δα, -Δβ, -Δγ, -Δδ, and -Δε cells

Cell lines were derived from HEK293-WT cells by individual disruption of *ATP5F1A*, *ATP5F1B*, *ATP5F1C*, *ATP5F1D* and *ATP5F1E* with gRNAs listed in Appendix Table S6.

In HEK293-Δα cells, each gRNA introduced a separate deletion of 2 nucleotides into exon I of *ATP5F1A*. The resulting frameshift changed 31 of 32 amino acids between residues 7 and 38, and then the sequence terminated before the cleavage site of the mitochondrial import sequence (Appendix Fig. S4A). In HEK293-Δβ cells, the pair of gRNAs deleted 172 nucleotides, removing most of exon I of *ATP5F1B*, including the initiation codon, with the expectation that no protein product would be produced (Appendix Fig. S4B). In HEK293-Δγ cells, the pair of gRNAs introduced a deletion of 19 nucleotides in exon I of *ATP5F1C*, including the first base of the initiator codon, preventing the production of the subunit (Appendix Fig. S4C). In HEK293-Δδ cells, the single gRNA introduced a deletion of 2 nucleotides in exon II, of *ATP5F1D*, causing a frameshift in codons 89–144, and producing a termination codon at position 145 (Appendix Fig. S4D). In HEK293-Δε cells, the pair of gRNAs introduced a deletion into *ATP5F1E* consisting of part of exon I (74 nucleotides), including the initiation codon, and part of intron I (144 nucleotides), thus preventing expression of the protein (Appendix Fig. S4E).

#### HEK293-Δc cells

They were derived with three pairs of gRNAs (Appendix Table S6). Deletions of 140, 97, and 55 bases were introduced, respectively, into exon IV of *ATP5MC1*, *ATP5MC2*, and *ATP5MC3* (Appendix Fig. S2). The deletion in *ATP5MC1* altered 29 of 30 amino acids across the precursor cleavage site of the c-subunit which then terminated. In *ATP5MC2* the edited protein sequence started four amino acids before the junction with the mature protein, and terminated nine amino acids later. The editing of *ATP5MC3* changed six amino acids so that the protein ended three residues before the import sequence cleavage site.

#### HEK293-Δ(c + OSCP) cells

A pair of gRNAs introduced a 197 bp deletion in *ATP5PO* in HEK293-Δc cells, including the entire exon I together with the initiation codon, and regions of flanking introns (Appendix Fig. S2D).

#### HAP1-ΔATPAF1 cells

A single gRNA targeting exon VI deleted two nucleotides, producing a frameshift and an early termination codon. Conceivably, a 206 amino acid product could be synthesized, identical to the sequence of ATPAF1 for 187 amino acids, followed by 19 altered residues before terminating 122 amino acids shorter than the WT protein (Appendix Fig. S10).

#### HAP1-ΔATPAF2 cells

The single gRNA generated a deletion of seventeen nucleotides near the 3’-end of exon I. The mutant cells encode a 52-amino-acid product, with 37 N-terminal residues identical to the WT sequence, followed by 15 unrelated residues (Appendix Fig. S10).

#### HAP1-ΔFMC1 cells

A single gRNA targeted to exon I introduced a deletion of two nucleotides and produced a nonsense mutation. The disrupted gene encodes a 48-amino-acid polypeptide with its C-terminus extended by four unrelated residues (Appendix Fig. S10).

#### HEK293-Δ(c + OSCP + ATPAF1) cells

A pair of gRNAs introduced a deletion of 363 nucleotides into *ATPAF1* in HEK293-Δ(c+OSCP) cells (Appendix Table S6) removing a fragment corresponding to almost the entire exon I, including the initiation codon (Appendix Fig. S14).

#### HEK293-Δ(c + OSCP + ATPAF2) cells

*ATPAF2* was disrupted in HEK293-Δ(c+OSCP) cells with a pair of gRNAs (Appendix Table S6) producing a deletion of 87 nucleotides in exon I, including the initiation codon (Appendix Fig. S14).

#### HEK293-Δ(c + OSCP + ΔFMC1) cells

*FMC1* was disrupted in HEK293-Δ(c+OSCP) cells with a pair of gRNAs (Appendix Table S6) producing a deletion of 126 nucleotides in exon I, including the initiation codon (Appendix Fig. S14).

#### HEK293-Δ(c + OSCP + ATPAF1 + ATPAF2) cells

*ATPAF1* and *ATPAF2* were each disrupted in HEK293-Δ(c+OSCP) cells with a pair of gRNAs (Appendix Table S6) producing deletions of 363 and 87 nucleotides, respectively, both in exon I and including the initiation codon (Appendix Fig. S15).

#### HEK293-Δ(c + OSCP + ATPAF2 + FMC1) cells

*ATPAF2* and *FMC1* were each disrupted with pairs of gRNAs (Appendix Table S6), producing deletions of 87 and 125 nucleotides, respectively, both in exon I and both including the initiation codon (Appendix Fig. S16).

#### HEK293-Δ(ATPAF1 + ATPAF2 + FMC1) cells

A pair of gRNAs introduced deletions of 362, 87, and 125 nucleotide codons into *ATPAF1*, *ATPAF2*, and *FMC1*, respectively, including the translation initiation in HEK293-WT cells (Appendix Table S6). The 362 nucleotides deleted from *ATPAF1* were replaced with a random 364 nucleotides containing an ATG codon upstream of the WT ATG, with the potential to produce a 43 amino acid sequence unrelated to the WT protein (Appendix Fig. S13).

### Expression and purification of proteins

Plasmids encoding ATP5F1C, ATP5F1D, ATP5F1E, ATPAF1, ATPAF2, and FMC1 were purchased from Source Bioscience. Each coding sequence was amplified by PCR from the corresponding plasmid with the oligonucleotide primers summarized in Appendix Table S8. The products were digested with *Hin*d III and *Xho* I and cloned individually in-frame between the *Hin*d III and *Xho* I sites of the expression plasmid pcDNA5^TM^-FRT/TO (ThermoFisher) with tandem C-terminal FLAG and Strep II tags. The resultant expression plasmids encode ATP5F1C-t, ATP5F1D-t, ATP5F1E-t, ATPAF1-t, ATPAF2-t, and FMC1-t, where “t” denotes tandem FLAG and Strep II tags at their C-termini. The PCR fragment encoding FMC1-t plus a 5’-T2A self-cleaving peptide sequence (Liu et al, 2017) with an N-terminal GSG linker (GSGEGRGSLLTCGDVEENPGP) was ligated (Gibson et al, 2009) with a second PCR fragment encoding ATPAF2 minus the termination codon, and cloned into plasmid pcDNA5^TM^/FLP/TO. The resulting expression plasmid named pcDNA5^TM^/FLP/TO-ATPAF2-T2A-FMC1-t (Appendix Fig. S19) was transfected into HEK293-Δ(c+OSCP + ATPAF2 + FMC1) cells. The translation products were predominantly ATPAF2-GSGEGRGSLLTCGDVEENPG (ATPAF2-T2A) and FMC1-t with an additional N-terminal proline residue (named P-FMC1-t). The expression plasmids were introduced into HEK293-WT cells, or derivatives thereof, by lipofectamine transfection (ThermoFisher) producing a stable expression cell line for each protein. The expression of proteins was induced for 3 days with doxycycline (20 ng/ml), or as otherwise specified in figure legends. Cells were disrupted by Dounce homogenization or with digitonin (detergent:protein, 1:10, g:g), and mitochondria or mitoplasts were recovered by centrifugation (10,000×*g*, 5–10 min). Mitochondria or mitoplasts were extracted with either digitonin (detergent:protein, 10:1, g:g) or DDM (detergent:protein, 2:1, g:g). These extracts were applied to a Strep-Tactin agarose gravity column (IBA-Lifesciences), and bound proteins were released with D-desthiobiotin.

#### Bacterial expression of δ- and ε-subunits

An expression plasmid pRun (Fiermonte et al, 2002) encoding the mature δ-subunit with an N-terminal His tag and TEV cleavage site, followed by an intervening sequence containing a Shine-Dalgarno motif and then the coding sequence of the ε-subunit, was transformed into *E. coli* C41 (DE3) (Miroux and Walker, 1996). Cells were grown in 2xTY medium at 37 °C until the OD_600_ reached *ca*. 0.6 absorbance units. Expression of the recombinant proteins was induced with isopropyl β-D-1-thiogalactoside (final concentration 600 μM), and cell growth continued for a further 17 h at 25 °C. Cells were centrifuged (2700×*g*, 15 min, 4 °C). The pellet was resuspended in buffer containing 20 mM Tris-HCl, pH 7.4, 0.1 M NaCl, and 10% (v/v) glycerol, re-centrifuged (2535×*g*, 15 min, 4 °C), and the pellet was kept at −20 °C. Frozen cells were resuspended in buffer containing 20 mM Tris-HCl, pH 7.4, 0.1 M NaCl, and cOmplete^TM^ EDTA-free protease inhibitor cocktail (Sigma-Aldrich), and disrupted at 4 °C with a Q700 sonicator (Qsonica, Connecticut, USA) with a probe 0.75 inches in diameter. Debris was removed by centrifugation, and the supernatant was filtered in a 0.22-μm Millex-GP syringe. Imidazole (5 M, pH 8.0) was added to a final concentration of 20 mM and the sample applied at room temperature to a HisTrap HP column (5 ml, GE Healthcare) equilibrated in 20 mM Tris-HCl, pH 7.4, 0.1 M NaCl, 20 mM imidazole. Bound proteins were eluted with a gradient of imidazole from 20 to 300 mM. A portion (10 ml) of the eluate containing both the tagged δ- and the ε-subunits was dialyzed into 20 mM Tris, pH 8.0, 10 mM NaCl, and 1 mM DTT, at 4 °C. The sample was passed through a 1 ml HiTrap SP HP cation exchange column equilibrated in the same buffer, at room temperature. The unbound fraction was dialyzed into 20 mM MES, pH 6.5, containing 1 mM DTT, at 4 °C, and applied to a 1 ml HiTrap SP HP column equilibrated in the same buffer at room temperature. Bound proteins were eluted with a NaCl gradient (2 steps; 60 ml for 0 to 0.3 M and 20 ml for 0.3 to 1 M). Three peaks were observed, each containing both the tagged δ-subunit and the ε-subunit. Each peak was dialyzed into 20 mM Tris-HCl, pH 7.4, 0.1 M NaCl, 1 mM DTT, at 4 °C. His-tagged TEV protease (*ca*. 0.1 mg/ml) was added to the dialyzing samples, which were placed into fresh dialysis buffer, at 30 °C, for 3 h. Imidazole was added to the peak 1 sample to a final concentration of 10 mM, then passed through a 1 ml HisTrap HP column, equilibrated in 20 mM Tris-HCl, pH 7.4, 0.1 M NaCl, 1 mM DTT, 10 mM imidazole, at room temperature. The unbound fraction, containing the δ- and ε-subunits, was dialyzed into 10 mM Tris-HCl, pH 7.4, 25 mM NaCl, 1 mM DTT, at 4 °C. The sample was characterized by native-PAGE, intact molecular mass measurement by electrospray MS in a Q-Trap 4000 (ABSciex) instrument operated in MS mode and calibrated with a mixture of myoglobin and trypsinogen, and gel filtration chromatography on a Superdex 200 Increase 10/300 GL column (GE Healthcare) with gel filtration calibrant proteins (GE Healthcare).

### Protein quantitation

Relative quantitation of proteins in SILAC samples (Ong et al, 2002) was performed by MS analysis of tryptic and chymotryptic digests of proteins (He et al, 2020; Carroll et al, 2021). Peptide mass data were analyzed with MaxQuant version 2.0.1.0, and the integrated Andromeda search engine (Cox and Mann, 2008; Cox et al, 2011), employing a UniProt human protein database (January 2021 or January 2022) modified to include the three mature forms of ATP5IF1, IF_1_-M1, -M2 and -M3, and the mature ATP synthase c-subunit (He et al, 2020; Carroll et al, 2021). Where required, protein ratios for UniProt entry Q96HJ9 (FMC1) were calculated manually from the MaxQuant evidence file, using only peptide ratios for isoform-1 and identified in the expected gel slice(s), and excluding peptides specific for isoform-2, which also occur in an unrelated entry Q9Y383 (LUC7L2). All manually calculated ratios represent the median of the assigned specific peptide ratios, where MaxQuant ISO-MSMS peptide values were used only if fewer than three peptide ratios were obtained by MULTI-MSMS. Significance B, calculated with a Benjamini-Hochberg correction (*P* = 0.05), was used as the marker of statistical significance. For the processing and analysis of the data, see (He et al, 2020).

### Structure predictions

Protein structures were predicted with AlphaFold 3 (Abramson et al, 2024). In structures involving ATPAF1 and ATPAF2, residues 1–51 and 1–42, respectively, were removed, as they represent the mitochondrial import sequences and their processing, as demonstrated here by N-terminal sequence analysis of purified ATPAF1-t and ATPAF2-t. Likewise, peptide data from FMC1 by numerous MS analyses showed that the N-terminal methionine had been removed and that Ala2 is acetylated. Images of structures were created with PyMol version 3.1.6.1 and associated Python scripts.

## Supplementary information

Appendix

Peer Review File

Source data Fig. 1

Source data Fig. 2

Source data Fig. 3

Source data Fig. 4

Source data Fig. 5

Source data Fig. 6

Source data Fig. 7

Expanded View Figures

## Data Availability

All data are available within the paper and Appendices, except for MS data. Processed MS datasets relating to the main figures are available as source data, with the raw MS data, MaxQuant evidence files, and additional processed datasets deposited to the ProteomeXchange Consortium via the PRIDE (Perez-Riverol et al, 2025) partner repository (https://www.ebi.ac.uk/pride/) with the dataset identifiers PXD077889, PXD077924, PXD077960, PXD079310, and PDX079319. The source data of this paper are collected in the following database record: biostudies:S-SCDT-10_1038-S44318-026-00842-9.
