# Supplementary material for: Assembly of the catalytic module and the rotor of human ATP synthase
Source: EMBO J. 2026 Jun 26;45(15):5482–513. doi: 10.1038/s44318-026-00842-9 (PMC13434801; doi:10.1038/s44318-026-00842-9)
Supplement: Supplementary file 1 [file 44318_2026_842_MOESM10_ESM.pdf]

Expanded View Figures

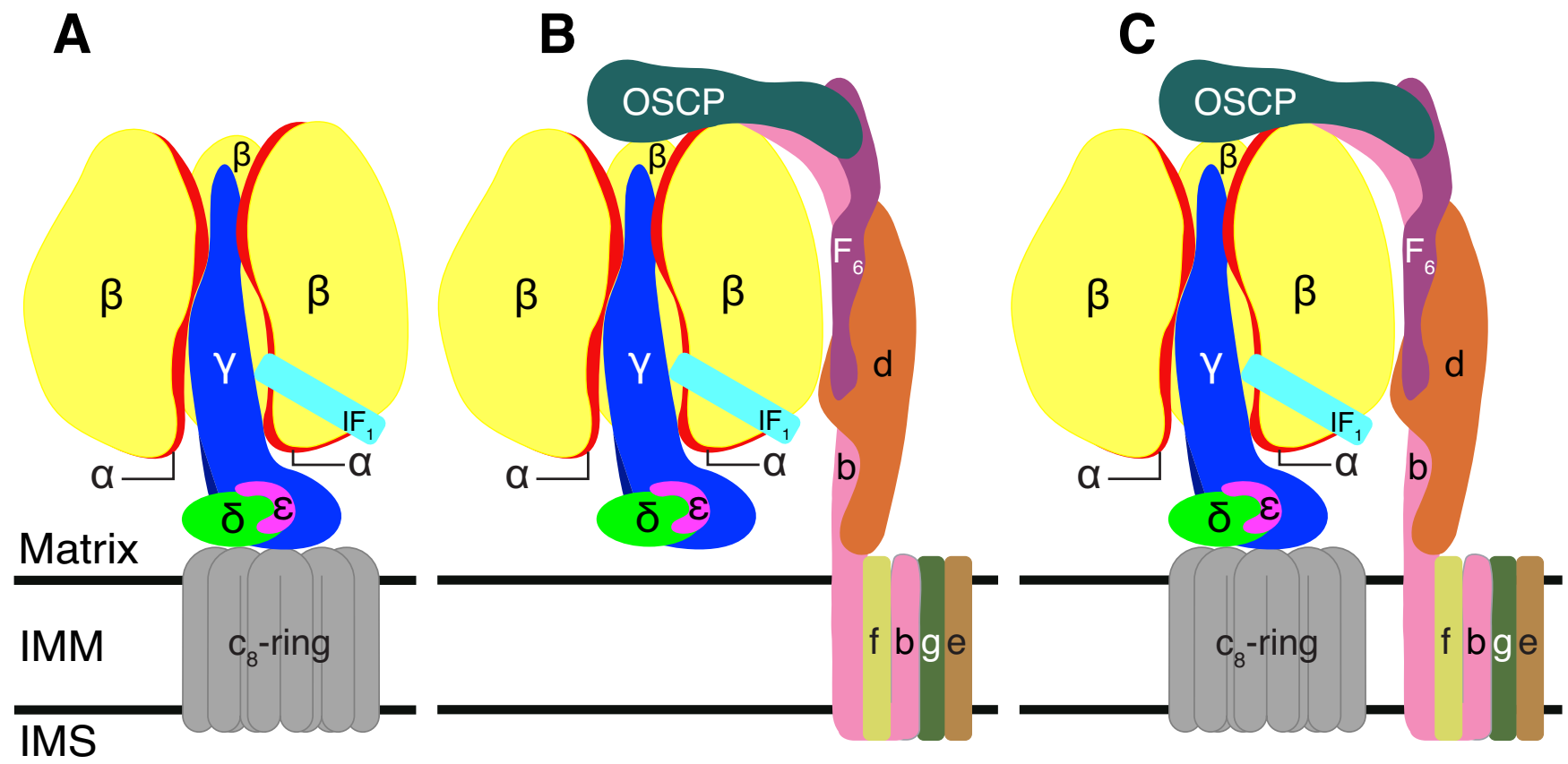

Figure EV1. Key sub-complexes of human ATP synthase known to be assembled in the absence of specific subunits.

The upper region of the CS has been exposed by the removal of an α-subunit (red). IF_1 is bound to all three complexes to prevent ATP hydrolysis. (**A**) Complex F_1-c_8 assembled in the absence of any one of the PS components OSCP, subunit b, or subunit F_6 (He et al, 2017a, 2020). (**B**) Complex F_1-PS plus e, f, and g, assembled in the absence of subunit c (He et al, 2017b). (**C**) Complex (referred to as "the key intermediate") assembled in the absence of mitochondrially encoded subunits ATP6 and ATP8 (He et al, 2017b), and arrived at by the addition of either the PS plus associated membrane subunits e, f, and g to intermediate (**A**), or the c_8-ring to complex (**B**).

© The Author(s)

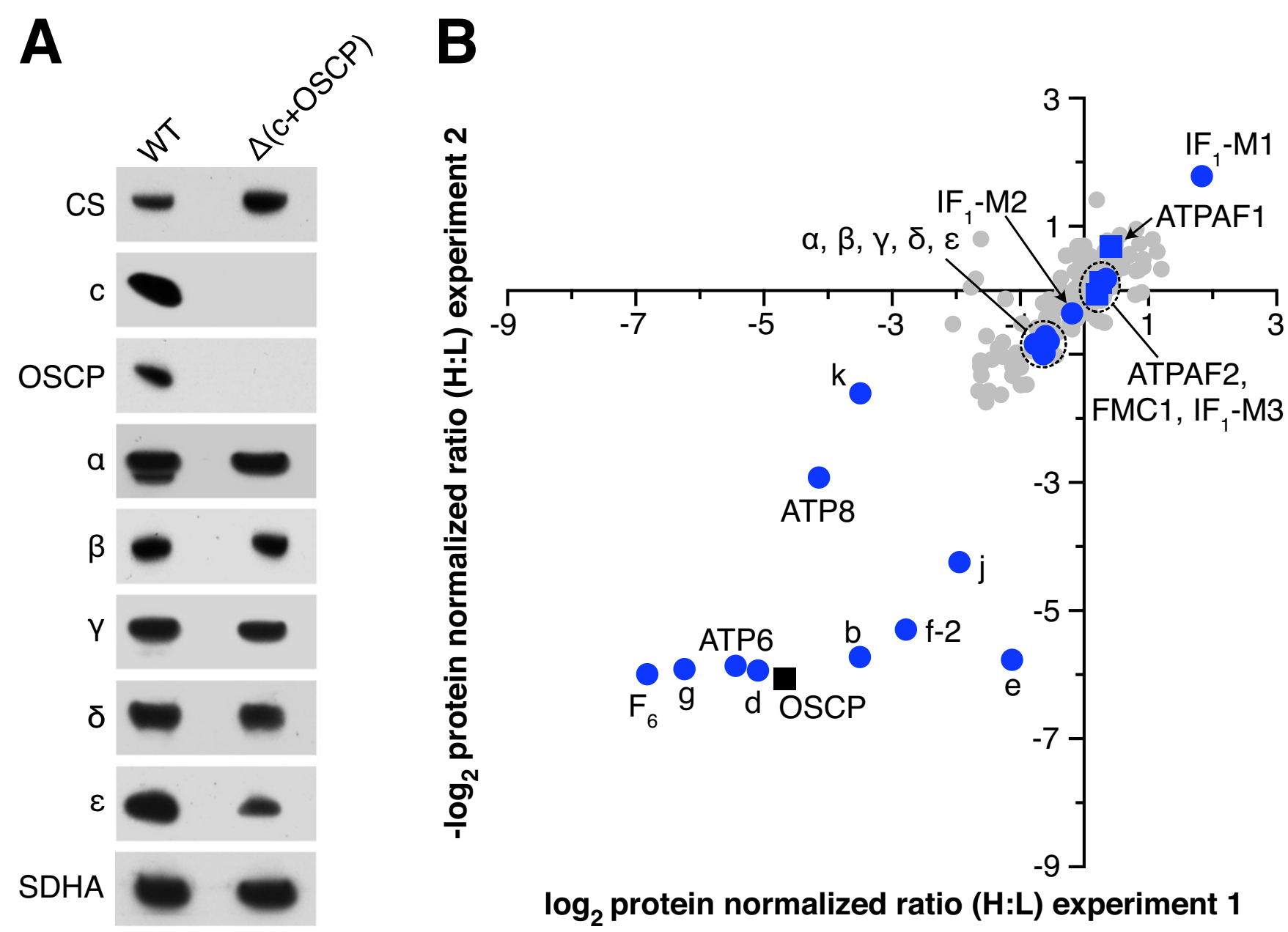

Figure EV2. Impact of deleting the OSCP and the c-subunit of h-ATP synthase together in HEK293 cells.

(**A**) Western blots of DDM extracts of mitoplasts from HEK293-WT and -Δ(c+OSCP) cells, with antibodies against the subunits indicated on the left. Citrate synthase (CS) and the SDHA subunit of complex II are loading controls. (**B**) Protein abundances in immunopurified ATP synthase from HEK293-Δ(c+OSCP) cells relative to HEK293-WT cells determined by SILAC-MS. The experiment was performed twice (biological replicates) with reciprocal SILAC labeling. The points on the scatter plots represent the log base 2 values of protein ratios from the two complementary MS analyses, normalized by centering on the median protein ratio. IF_1-M1, -M2, and -M3 are specific mature forms of IF_1 (He et al, 2017b); f-2 is an isoform of subunit f (UniProt P56134). Protein ratios were derived from a minimum of two peptide ratios, except ATP6 and ATPAF2 in experiment 1 where the value is derived from a single peptide ratio. No ratios were obtained for subunit c. Blue circles, ATP synthase subunits and forms of IF_1; blue squares, assembly factors ATPAF1, ATPAF2, and FMC1; black square, deleted subunit; gray circles, all other identified proteins.

 © The Author(s)

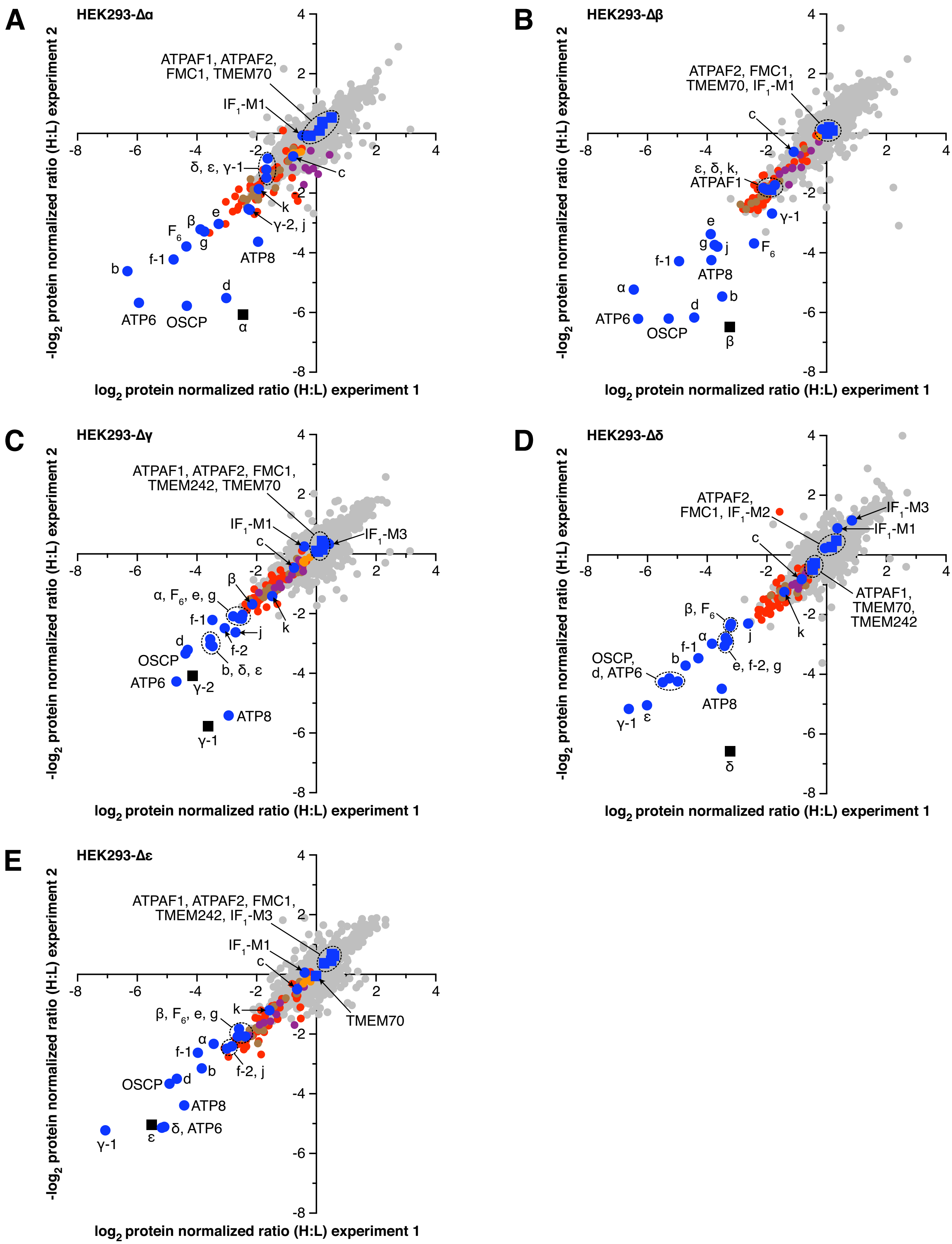

A
HEK293-Δα
ATPAF1, ATPAF2, FMC1, TMEM70
IF1-M1
δ, ε, γ-1
c
k
γ-2, j
e
β
F6
g
f-1
b
ATP8
d
ATP6
OSCP
α
B
HEK293-Δβ
ATPAF2, FMC1, TMEM70, IF1-M1
c
ε, δ, k, ATPAF1
γ-1
e
g
j
F6
f-1
ATP8
α
b
d
ATP6
OSCP
β
C
HEK293-Δγ
ATPAF1, ATPAF2, FMC1, TMEM242, TMEM70
IF1-M1
c
IF1-M3
β
α, F6, e, g
k
f-1
j
f-2
d
b, δ, ε
OSCP
ATP6
γ-2
ATP8
γ-1
D
HEK293-Δδ
ATPAF2, FMC1, IF1-M2
IF1-M3
IF1-M1
c
ATPAF1, TMEM70, TMEM242
β, F6
k
α
j
f-1
b
e, f-2, g
OSCP, d, ATP6
ATP8
γ-1
ε
δ
E
HEK293-Δε
ATPAF1, ATPAF2, FMC1, TMEM242, IF1-M3
IF1-M1
c
TMEM70
k
β, F6, e, g
α
f-1
f-2, j
b
OSCP
d
ATP8
δ, ATP6
ε
γ-1
-log2 protein normalized ratio (H:L) experiment 2
log2 protein normalized ratio (H:L) experiment 1

Figure EV3. Quantitative impact of individual deletion of subunits of the F_1-module of h-ATP synthase in HEK293 cells.

Shown are protein abundances in mitoplasts from cells lacking each of the five subunits determined by SILAC-MS analysis relative to HEK293-WT cells. (**A–E**) HEK293-Δα, -Δβ, -Δγ, -Δδ, and -Δε cells, respectively. Black square, the deleted subunit; blue circles, subunits of ATP synthase; blue squares, assembly factors for ATP synthase; red circles, complex I subunits; orange circles, complex II subunits; purple circles, complex III subunits; brown circles, complex IV subunits; gray circles, all other identified proteins. The experiments were performed twice (biological replicates) with reciprocal SILAC labeling. The points on the scatter plot represent the log base 2 values of normalized protein ratios from the two complementary MS analyses. For the definition of IF_1-M1, IF_1-M3, f-1, and f-2, see legend to Fig. EV2. γ-1 and γ-2 are isoforms of the γ-subunit (UniProt P36542). Ratios for two non-interacting proteins PLXNA3 (4.37, 0.411) and ANP32B (5.01, −0.701) fall outside of the axes in (**A**, **C**), respectively.

 © The Author(s)

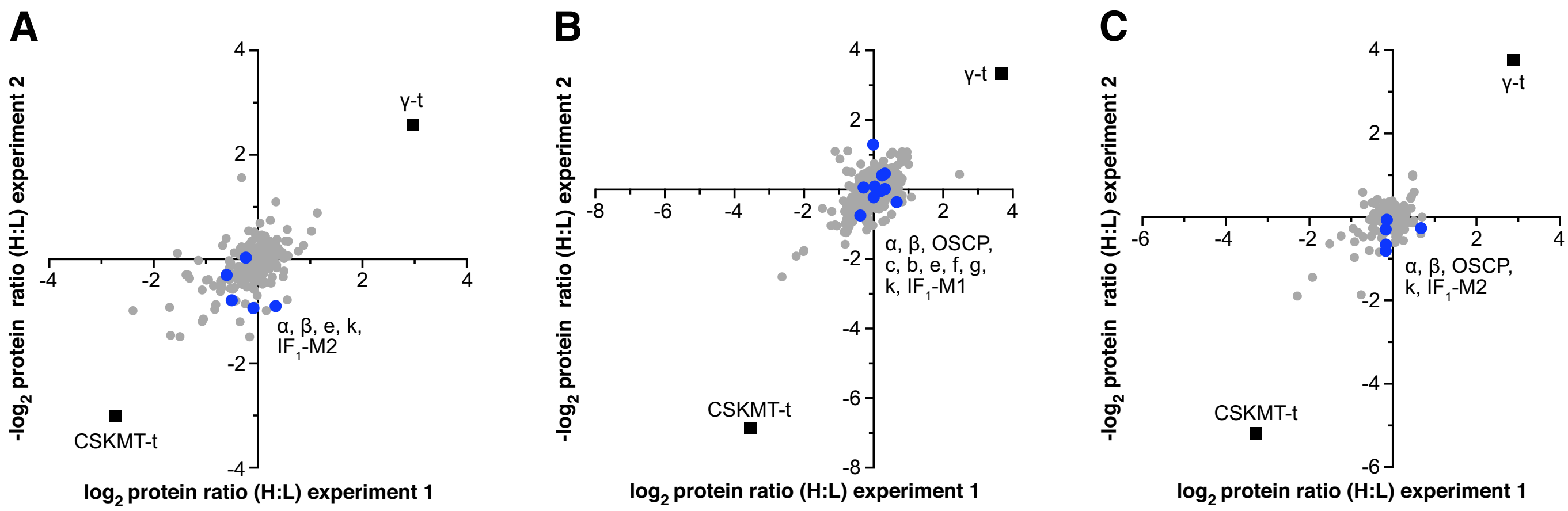

Figure EV4. Failure of the γ-subunit of h-ATP synthase to interact with the α- or β-subunits in the absence of either the δ- or the ε-subunit.

Relative abundances determined by quantitative MS of proteins associated with γ-t in SILAC-labeled cells. (**A**) HEK293-Δε cells and in (**B**) and (**C**), HEK293-Δδ cells. The mitochondrial protein CSKMT was expressed as the tagged reciprocal control protein. In (**C**), but not in (**B**), the buffer for solubilization and affinity purification of the proteins contained 1 mM $MgCl_2$ and 0.2 mM ADP, and the procedures performed at 20 °C rather than 4 °C (as in **B**). The points on the scatter plot represent the log base 2 values of the protein ratios from two complementary MS analyses (biological replicates), normalized by centering on the median protein ratio. Any proteins specifically associated with γ-t would be in the upper right quadrant with γ-t. Non-specifically associated proteins common to both experiments (γ-t and CSKMT-t) are found near the intersection of the axes. CSKMT-t is in the bottom left quadrant. Black squares, tagged proteins; blue circles, ATP synthase subunits; gray circles, all other identified proteins. IF_1-M2 is a specific mature form of IF_1 (He et al, 2017b).

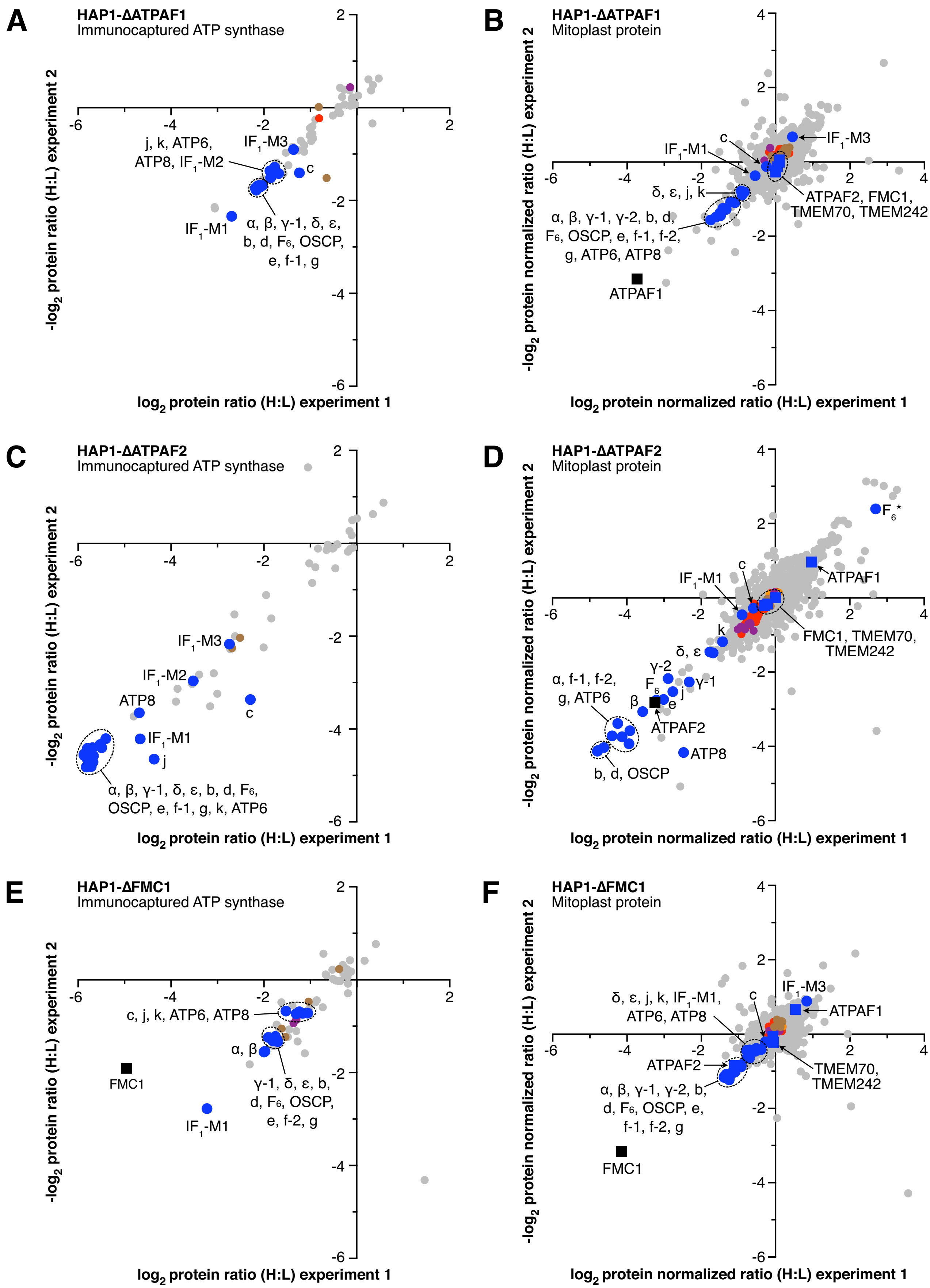
A
HAP1-ΔATPAF1
Immunocaptured ATP synthase
j, k, ATP6, ATP8, IF1-M2
IF1-M3
c
IF1-M1
α, β, γ-1, δ, ε, b, d, F6, OSCP, e, f-1, g
-log2 protein ratio (H:L) experiment 2
log2 protein ratio (H:L) experiment 1
B
HAP1-ΔATPAF1
Mitoplast protein
c
IF1-M1
IF1-M3
δ, ε, j, k
ATPAF2, FMC1, TMEM70, TMEM242
α, β, γ-1, γ-2, b, d, F6, OSCP, e, f-1, f-2, g, ATP6, ATP8
ATPAF1
-log2 protein normalized ratio (H:L) experiment 2
log2 protein normalized ratio (H:L) experiment 1
C
HAP1-ΔATPAF2
Immunocaptured ATP synthase
IF1-M3
IF1-M2
ATP8
c
IF1-M1
j
α, β, γ-1, δ, ε, b, d, F6, OSCP, e, f-1, g, k, ATP6
-log2 protein ratio (H:L) experiment 2
log2 protein ratio (H:L) experiment 1
D
HAP1-ΔATPAF2
Mitoplast protein
F6*
ATPAF1
c
IF1-M1
k
δ, ε
FMC1, TMEM70, TMEM242
γ-2
F6
γ-1
j
e
β
α, f-1, f-2, g, ATP6
ATPAF2
ATP8
b, d, OSCP
-log2 protein normalized ratio (H:L) experiment 2
log2 protein normalized ratio (H:L) experiment 1
E
HAP1-ΔFMC1
Immunocaptured ATP synthase
c, j, k, ATP6, ATP8
α, β
FMC1
γ-1, δ, ε, b, d, F6, OSCP, e, f-2, g
IF1-M1
-log2 protein ratio (H:L) experiment 2
log2 protein ratio (H:L) experiment 1
F
HAP1-ΔFMC1
Mitoplast protein
δ, ε, j, k, IF1-M1, ATP6, ATP8
c
IF1-M3
ATPAF1
ATPAF2
TMEM70, TMEM242
α, β, γ-1, γ-2, b, d, F6, OSCP, e, f-1, f-2, g
FMC1
-log2 protein normalized ratio (H:L) experiment 2
log2 protein normalized ratio (H:L) experiment 1

 © The Author(s)

Figure EV5. Impact of individual deletion of assembly factors for the F_1-module of h-ATP synthase on the abundances of subunits of the enzyme and of the assembly factors.

(**A**, **C**, **E**) Protein abundances in samples of immunocaptured ATP synthase or (**B**, **D**, **F**) in mitoplasts from HAP1 cells lacking each of the F_1-domain assembly factors, determined by SILAC-MS analysis relative to HAP1-WT cells. (**A**, **B**) HAP1-ΔATPAF1 cells. (**C**, **D**) HAP1-ΔATPAF2 cells. (**E**, **F**) HAP1-ΔFMC1 cells. Black square, deleted protein; blue circles, subunits of ATP synthase; blue squares, assembly factors for ATP synthase; red circles, complex I subunits; orange circles, complex II subunits; purple circles, complex III subunits; brown circles, complex IV subunits; gray circles, all other identified proteins. The experiments were performed twice (biological replicates) with reciprocal SILAC labeling. The points on the scatter plot represent the log base 2 values of raw (**A**, **C**, **E**) or normalized (**B**, **D**, **F**) protein ratios from the two complementary MS analyses. For definition of IF_1-M1, IF_1-M3, γ-1, γ-2, f-1, and f-2, see legend to Figs. EV2 and EV3. (**D**) The ratios for F_6 and F_6* correspond to the mature and precursor forms of the subunit, which migrated to different positions in the SDS-PAGE gel, and were manually calculated using the peptide ratio output from the MaxQuant program.

© The Author(s)

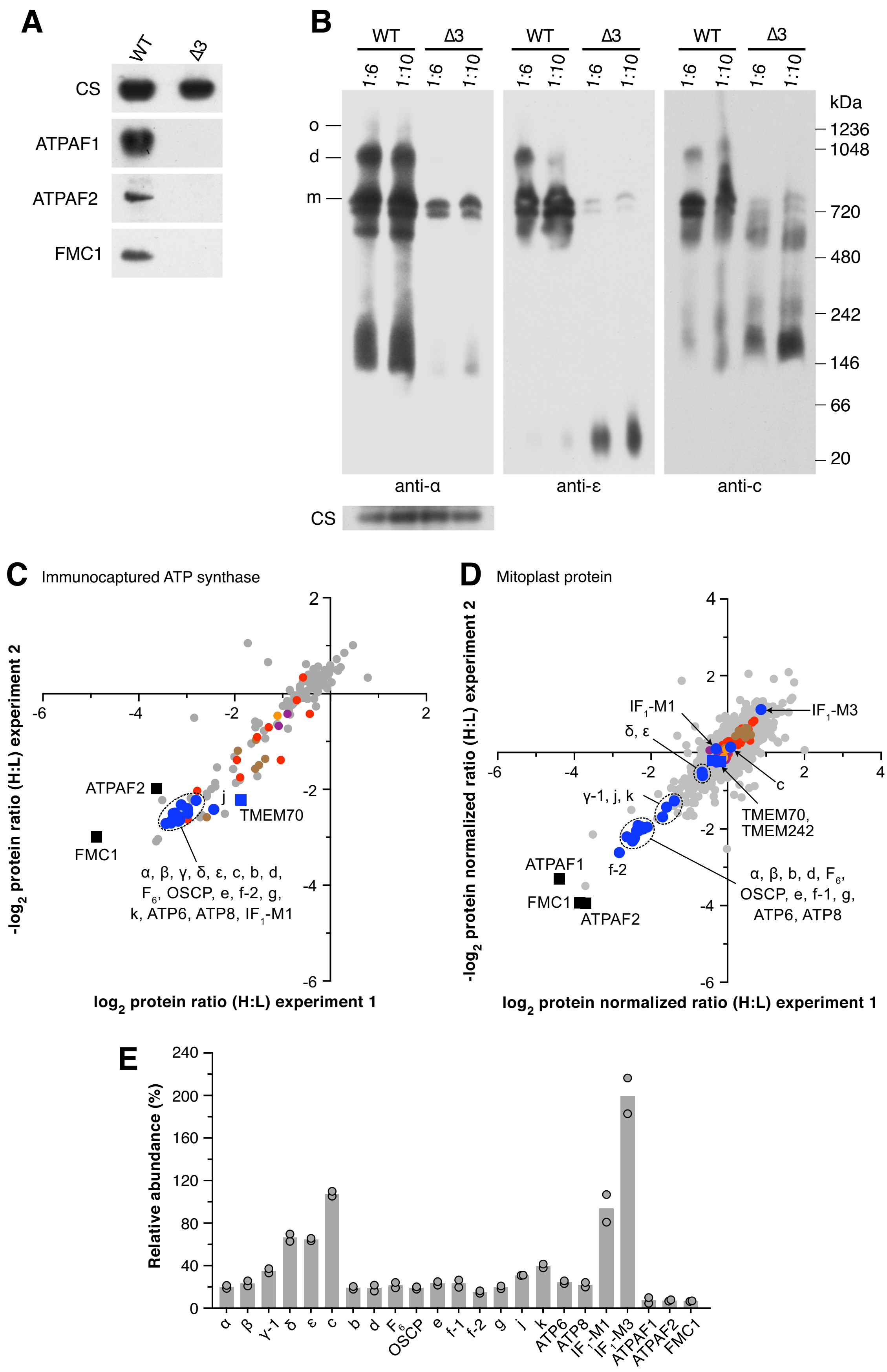
A
WT
Δ3
CS
ATPAF1
ATPAF2
FMC1
B
WT
Δ3
1:6
1:10
o
d
m
kDa
1236
1048
720
480
242
146
66
20
anti-α
anti-ε
anti-c
CS
C
Immunocaptured ATP synthase
ATPAF2
FMC1
j
TMEM70
α, β, γ, δ, ε, c, b, d, F6, OSCP, e, f-2, g, k, ATP6, ATP8, IF1-M1
-log2 protein ratio (H:L) experiment 2
log2 protein ratio (H:L) experiment 1
D
Mitoplast protein
IF1-M1
IF1-M3
δ, ε
c
γ-1, j, k
TMEM70, TMEM242
f-2
ATPAF1
FMC1
ATPAF2
α, β, b, d, F6, OSCP, e, f-1, g, ATP6, ATP8
-log2 protein normalized ratio (H:L) experiment 2
log2 protein normalized ratio (H:L) experiment 1
E
Relative abundance (%)
α
β
γ-1
δ
ε
c
b
d
F6
OSCP
e
f-1
f-2
g
j
k
ATP6
ATP8
IF1-M1
IF1-M3
ATPAF1
ATPAF2
FMC1

 © The Author(s)

Figure EV6. Characterization of HEK293-Δ(ATPAF1 + ATPAF2 + FMC1) cells and the impact on assembly of h-ATP synthase.

(**A**) Western blots of SDS-PAGE analyses of DDM extracts of HEK293-WT and -Δ(ATPAF1 + ATPAF2 + FMC1) cells (denoted as Δ3). The specificities of the antibodies are indicated on the left. (**B**) Western blots of BN-PAGE analyses of digitonin extracts (6 g/g or 10 g/g protein) of HEK293-WT and -Δ(ATPAF1 + ATPAF2 + ATPAF3) cells. The specificity of the antibody is given beneath each panel. CS, citrate synthase, provided a loading control; o, d, and m, denote oligomeric, dimeric, and monomeric forms of ATP synthase, respectively. (**C**, **D**) Protein abundances in immunopurified ATP synthase and mitoplasts, respectively, from HEK293-Δ(ATPAF1 + ATPAF2 + FMC1) cells relative to HEK293-WT cells determined by SILAC and MS analysis. The experiment was performed twice with reciprocal SILAC labeling. The points on the scatter plots represent the log base 2 values of protein ratios from the two complementary MS analyses (biological replicates), with normalized values shown in (**D**). Black squares, the deleted assembly factors; blue circles, subunits of ATP synthase; blue squares, assembly factors for ATP synthase; red circles, complex I subunits; orange circles, complex II subunits; purple circles, complex III subunits; brown circles, complex IV subunits; gray circles, all other identified proteins. (**E**) Normalized average subunit abundances (%, $n = 2$) in mitoplasts of the two experimental values (gray circles) for ATP synthase subunits, the inhibitor protein IF_1, and assembly factors ATPAF1, ATPAF2, and FMC1. For definition of IF_1-M1, IF_1-M3, γ-1, f-1 and f-2, see legend to Figs. EV2 and EV3.

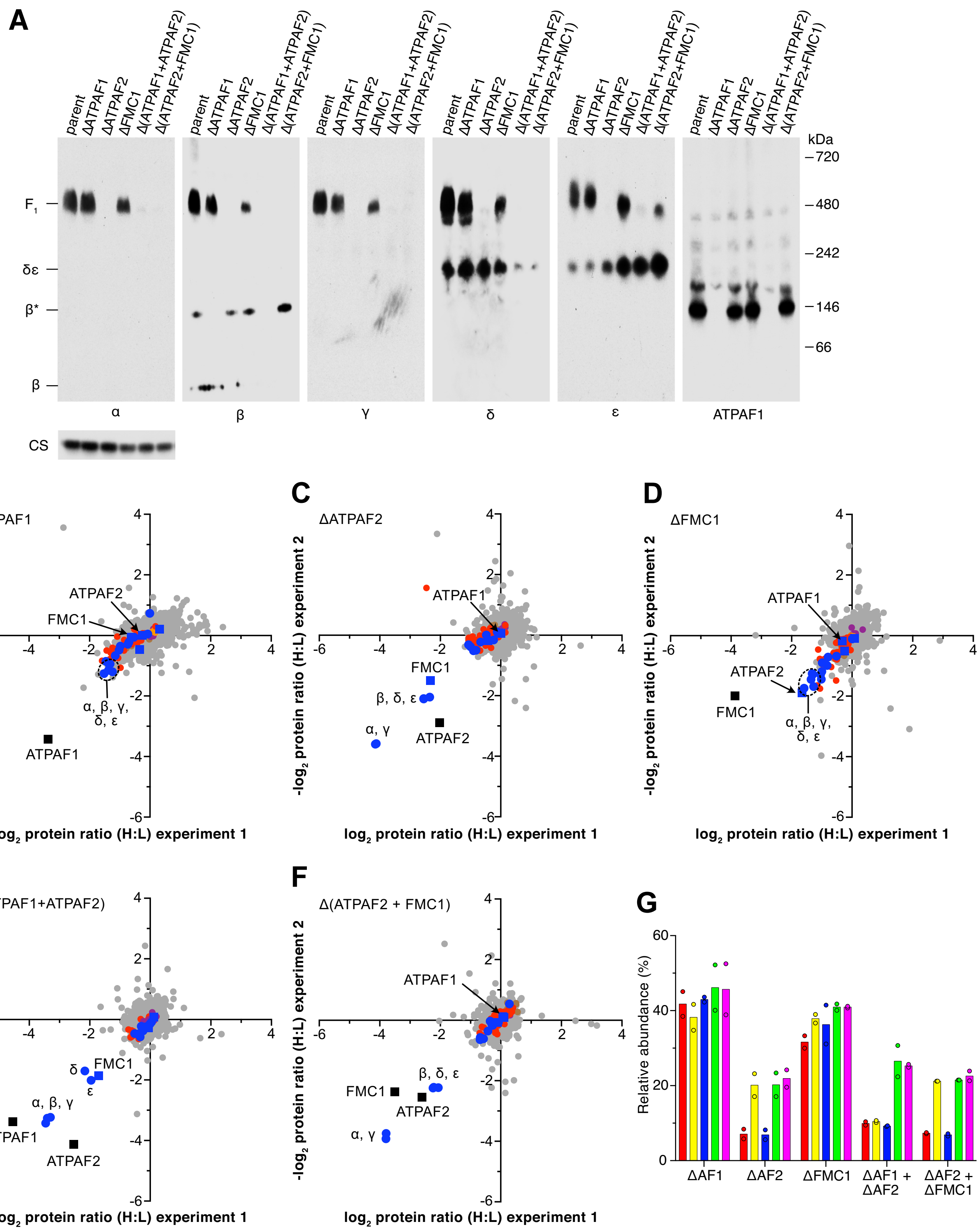
A
parent
ΔATPAF1
ΔATPAF2
ΔFMC1
Δ(ATPAF1+ATPAF2)
Δ(ATPAF2+FMC1)
kDa
720
480
242
146
66
F1
δε
β*
β
α
β
γ
δ
ε
ATPAF1
CS
B
ΔATPAF1
ATPAF2
FMC1
α, β, γ, δ, ε
ATPAF1
C
ΔATPAF2
ATPAF1
FMC1
β, δ, ε
α, γ
ATPAF2
D
ΔFMC1
ATPAF1
ATPAF2
FMC1
α, β, γ, δ, ε
E
Δ(ATPAF1+ATPAF2)
δ
FMC1
ε
α, β, γ
ATPAF1
ATPAF2
F
Δ(ATPAF2 + FMC1)
ATPAF1
β, δ, ε
FMC1
ATPAF2
α, γ
-log2 protein ratio (H:L) experiment 2
log2 protein ratio (H:L) experiment 1
G
Relative abundance (%)
ΔAF1
ΔAF2
ΔFMC1
ΔAF1 + ΔAF2
ΔAF2 + ΔFMC1

© The Author(s)

Figure EV7. Effect of the deletion of the assembly factors for the F_1-module of h-ATP synthase from HEK293-Δ(c + OSCP) cells.

(**A**) The assembly factors were deleted singly from HEK293-Δ(c+OSCP) cells (or "parent"), denoted above as ΔATPAF1, ΔATPAF2, and ΔFMC1. The pairwise combinations of ATPAF1 and ATPAF2 and ATPAF2 and FMC1 in HEK293-Δ(c+OSCP) cells are denoted as Δ(ATPAF1 + ΔATPAF2) and Δ(ATPAF2 + ΔFMC1), respectively. Shown are western blots of CN-PAGE fractionated extracts of mitoplasts from HEK293-Δ(c+OSCP) and derivative cells prepared with digitonin (10:1, g:g of protein), probed with antibodies with the specificities denoted beneath each panel. On the left, F_1, F_1-domain, δε, the δε-complex, β*, complex of the β-subunit with ATPAF1, and β, the free β-subunit. The positions of protein molecular weight markers are shown on the right. Citrate synthase, CS, detected by western blotting of samples fractionated by SDS-PAGE, was employed as a loading control. (**B–F**) Protein abundances were measured in mitoplasts from HEK293-Δ(c+OSCP) cells lacking the assembly factors, relative to HEK293-Δ(c+OSCP) cells, by SILAC-MS analysis. The experiments were performed twice (biological replicates) with reciprocal SILAC labeling. The scatter plot shows the relative protein abundances, represented as the log base 2 values of normalized protein ratios from the two complementary MS analyses. (**B**) HEK293-Δ(c+OSCP + ATPAF1), (**C**) HEK293-Δ(c+OSCP + ATPAF2), (**D**) HEK293-Δ(c+OSCP + FMC1), (**E**) HEK293-Δ(c+OSCP + ATPAF1 + ATPAF2), (**F**) HEK293-Δ(c+OSCP + ATPAF2 + FMC1) cells, respectively. Black square, the deleted protein; blue circles, subunits of ATP synthase; blue squares, assembly factors for ATP synthase; red circles, complex I subunits; orange circles, complex II subunits; purple circles, complex III subunits; brown circles, complex IV subunits; gray circles, all other identified proteins. (**G**) Relative abundances (average % subunit abundance, $n = 2$) of the two experimental values (colored circles) of the subunits of the F_1-domain in HEK293-Δ(c+OSCP) cells, where the assembly factors were removed singly and in combination: Red bar, α-subunit; yellow bar, β-subunit; blue bar, γ-subunit; green bar, δ-subunit; magenta bar, ε-subunit.

© The Author(s)

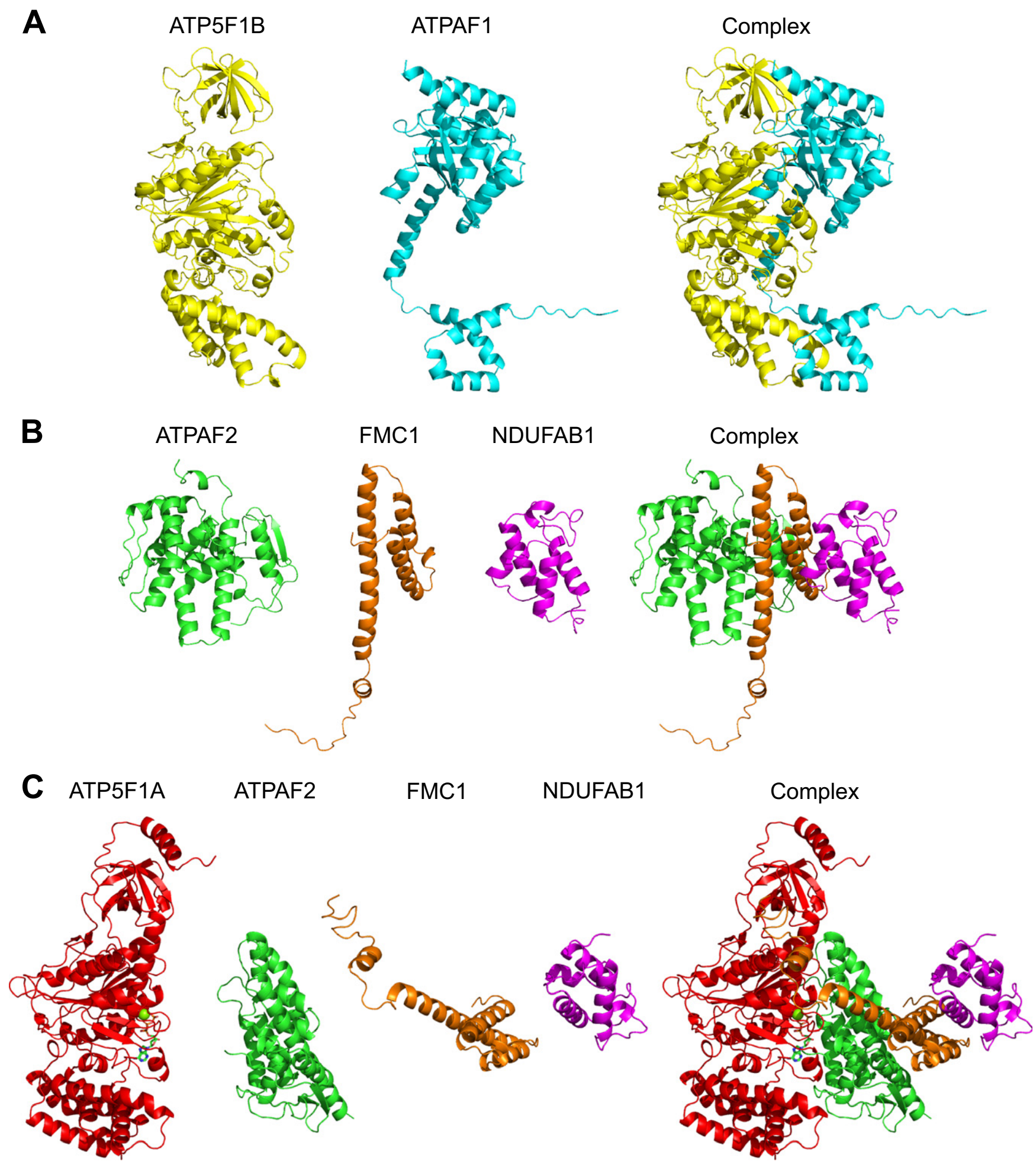

Figure EV8. Structures of assembly complexes for the F_1 module of h-ATP synthase.

Model 0 structures predicted with AlphaFold 3 (Abramson et al, 2024) from the mature protein sequences are shown. ATP5F1B (β-subunit) (P06576; residues 47–529), ATPAF1 (Q5TC12, residues 52–328), ATPAF2 (Q8N5M1, residues 43–289), FMC1 (Q96HJ9; residues 2–113), NDUFAB1 (O14561; residues 69–156) lacking a 4′-phosphopantetheine-acyl chain modification on Ser-112 (Ser-44 of the mature protein) and ATP5F1A (α-subunit) (P25705; residues 44–553). (**A**) ATP5F1B and ATPAF1 proteins and complex (ipTM 0.78, pTM 0.73, ranking score 0.81). (**B**) ATPAF2, FMC1, and NDUFAB1 proteins and complex (ipTM 0.89, pTM 0.86, ranking score 0.92). (**C**) ATP5F1A, ATPAF2, FMC1, and NDUFAB1 proteins and complex (ipTM 0.87, pTM 0.88, ranking score 0.89). The Mg^{2+} ion and ATP were also added, and incorporated into the ATP5F1A model.

 © The Author(s)

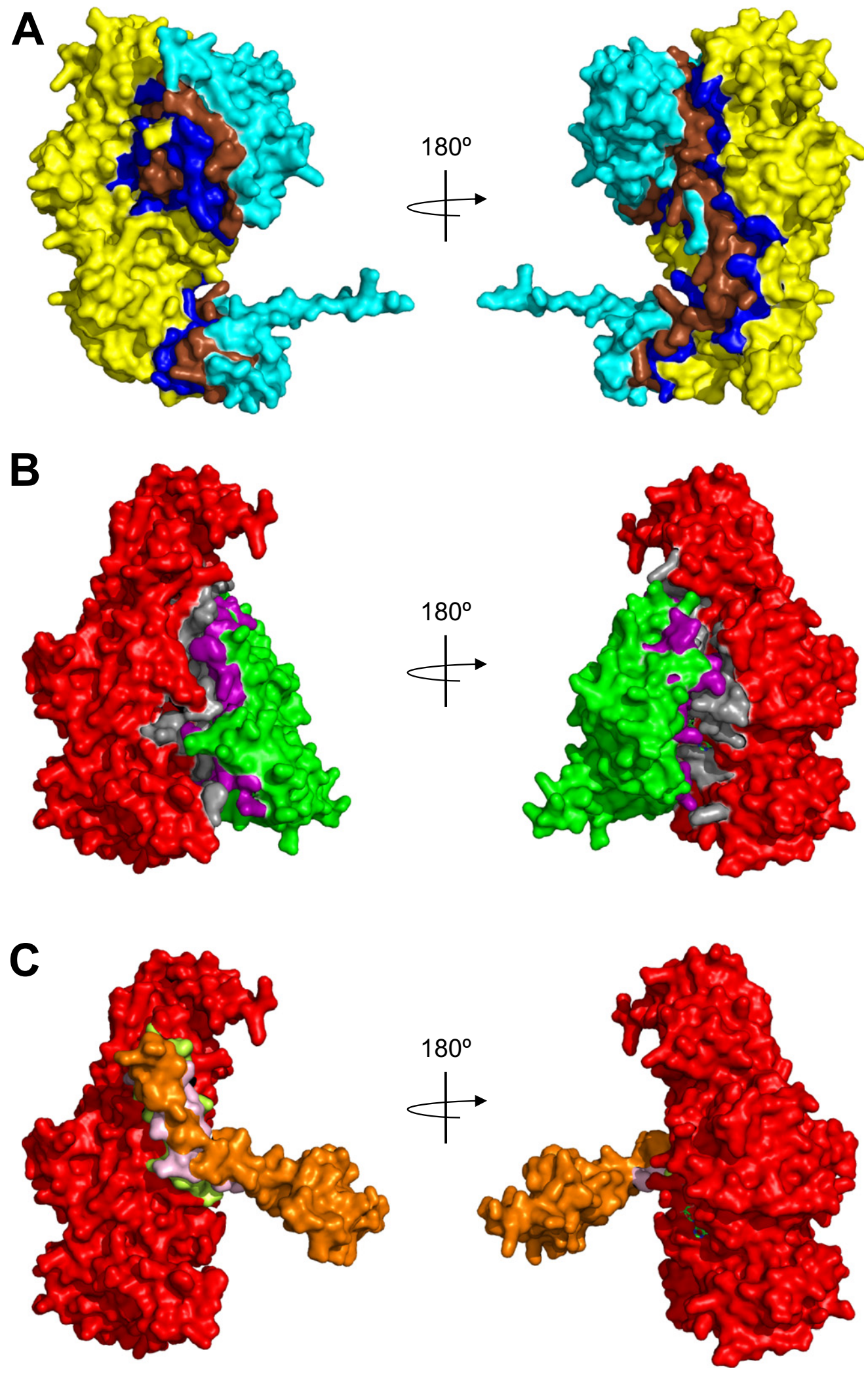

Figure EV9. Interactions of the α- and β-subunits with assembly factors.

The structures were predicted with AlphaFold 3, and modeled in surface representation with PyMol with the assistance of the script InterfaceResidues for depicting interface residues. (**A**) β-ATPAF1 complex. The body of the β-subunit is yellow, with residues in the interface with ATPAF1 in blue. The body of ATPAF1 is cyan with interface residues in brown. (**B**) Interaction of the α-subunit with FMC1 in the α-ATPAF2-FMC1-NDUFAB1 complex. The body of the α-subunit is red with residues in the interface with ATPAF1 in gray. The body of ATPAF2 is green with the interacting residues in purple. (**C**) Interaction of the α-subunit with FMC1 in the α-ATPAF2-FMC1-NDUFAB1 complex. The body of the α-subunit is red with residues in the interface with FMC1 in lime. The body of FMC1 is orange, and interacting residues are pink.

© The Author(s)

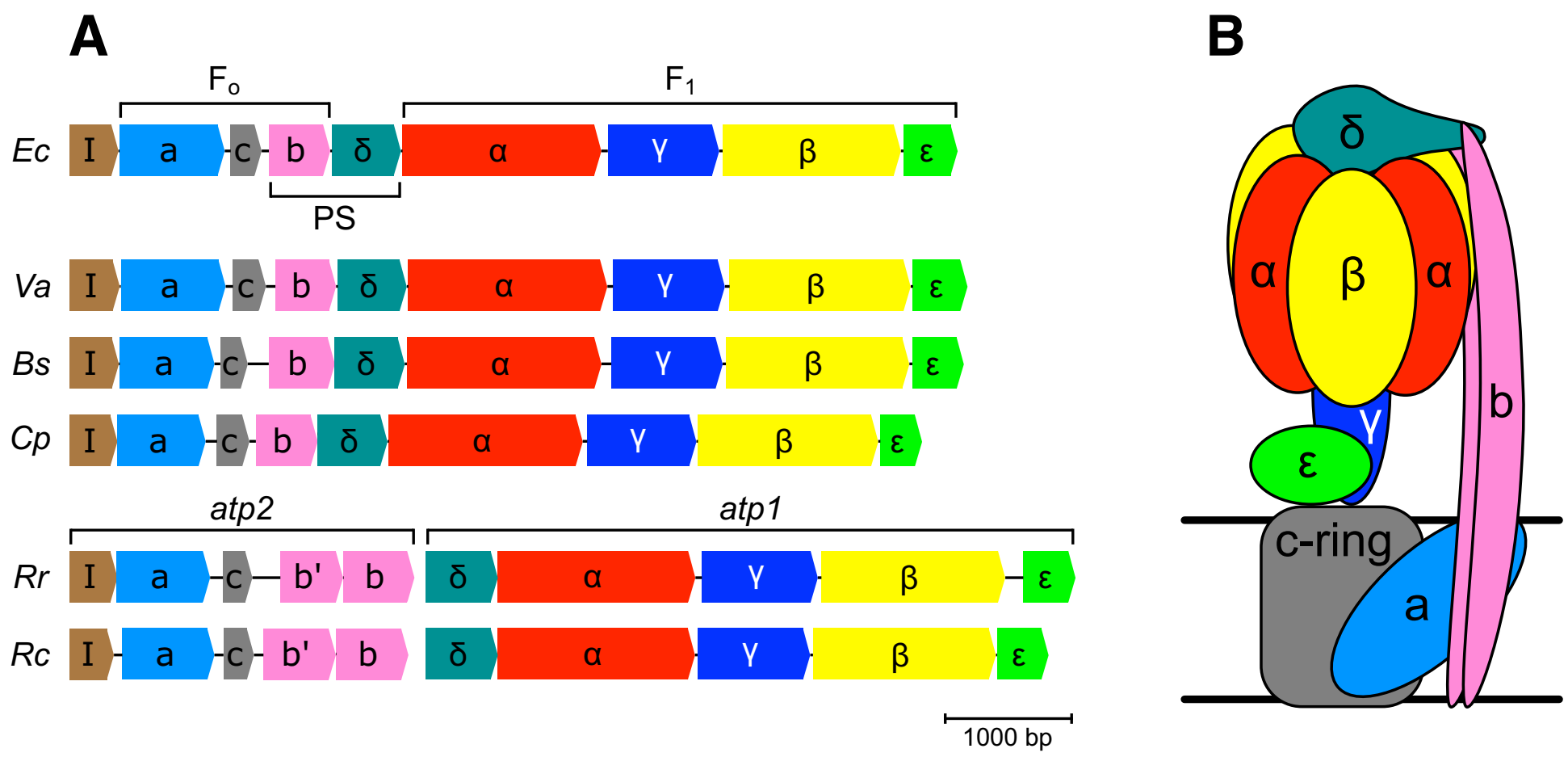

Figure EV10. Organization of genes for subunits of various bacterial ATP synthases.

Their subunit compositions are simpler than those of mitochondrial enzymes, but essential core features of the rotor and stator are retained. (**A**) Examples of *atp* operons from two Gram-negative (*Ec* and *Va*) (Walker et al, 1984; Krumholz et al, 1989), two Gram-positive (*Bs* and *Cp*) (Santana et al, 1994; Das and Ljungdahl, 2003) and two Gram-negative purple non-sulfur (*Rr* and *Rc*) (Falk et al, 1985; Falk and Walker, 1988; Borghese et al, 1998a, 1998b) bacteria. *Ec*, *Escherichia coli*. *Va*, *Vibrio alginolyticus*. *Bs*, *Bacillus subtilis*. *Cp*, *Clostridium pasteurianum*. *Rr*, *Rhodospirillum rubrum*. *Rc*, *Rhodobacter capsulatus*. The *Ec* operon is an example of a single operon archetypal arrangement. The genes are clustered into those encoding subunits of the F_o, peripheral stalk (PS), and F_1-modules. The promoter proximal genes (*uncI* or *atpI*) encode an integral membrane protein assembly factor for the c-ring module (Suzuki et al, 2007). The sizes of c-rings vary between species. In purple non-sulfur bacteria, the genes are organized into two separate operons, *atp1* encoding the F_1-domain, and *atp2* encoding the F_o-domain, with the PS genes straddling both operons. (**B**) Organization of the subunits of eubacterial ATP synthases. The δ- and ε-subunits are the equivalent subunits of human OSCP and δ, respectively (Walker et al, 1982). The bacterial PS consists of either two identical b-subunits or single copies of related b′- and b-subunits. Each has a single membrane spanning α-helix at its N-terminus.

 © The Author(s)
