# Supplementary material for: Assembly of the catalytic module and the rotor of human ATP synthase
Source: EMBO J. 2026 Jun 26;45(15):5482–513. doi: 10.1038/s44318-026-00842-9 (PMC13434801; doi:10.1038/s44318-026-00842-9)
Supplement: Supplementary file 2 [file 44318_2026_842_MOESM1_ESM.pdf]

APPENDIX for:

Assembly of the catalytic module and the rotor of human ATP synthase

Jiuya He, Joe Carroll, Shujing Ding, Jiao Li, Ian M. Fearnley and John E. Walker

Table of Contents **Page**

Appendix Figure S1 ……………………………………………………………………….. 2
Appendix Figure S2 ……………………………………………………………………….. 3
Appendix Figure S3 ……………………………………………………………………….. 4
Appendix Figure S4 ……………………………………………………………………….. 5
Appendix Figure S5 ……………………………………………………………………….. 6
Appendix Figure S6 ……………………………………………………………………….. 7
Appendix Figure S7 ……………………………………………………………………….. 8
Appendix Figure S8 ……………………………………………………………………….. 9
Appendix Figure S9 ………………………………………………………………………… 10
Appendix Figure S10 ……………………………………………………………………... 11
Appendix Figure S11 ……………………………………………………………………... 12
Appendix Figure S12 ……………………………………………………………………... 13
Appendix Figure S13 ……………………………………………………………………... 14
Appendix Figure S14 ……………………………………………………………………... 15
Appendix Figure S15 ……………………………………………………………………... 16
Appendix Figure S16 ……………………………………………………………………... 17
Appendix Figure S17 ……………………………………………………………………... 18
Appendix Figure S18 ……………………………………………………………………... 19
Appendix Figure S19 …………………………………………………………………….. 20
Appendix Figure S20 …………………………………………………………………….. 21
Appendix Figure S21 …………………………………………………………………….. 22
Appendix Figure S22 …………………………………………………………………….. 23

Appendix Table S1 ………………………………………………………………………..... 24
Appendix Table S2 ………………………………………………………………………..... 26
Appendix Table S3 ………………………………………………………………………..... 27
Appendix Table S4 ………………………………………………………………………..... 28
Appendix Table S5 ………………………………………………………………………..... 29
Appendix Table S6 ………………………………………………………………………..... 30
Appendix Table S7 ………………………………………………………………………..... 31
Appendix Table S8 ………………………………………………………………………..... 32

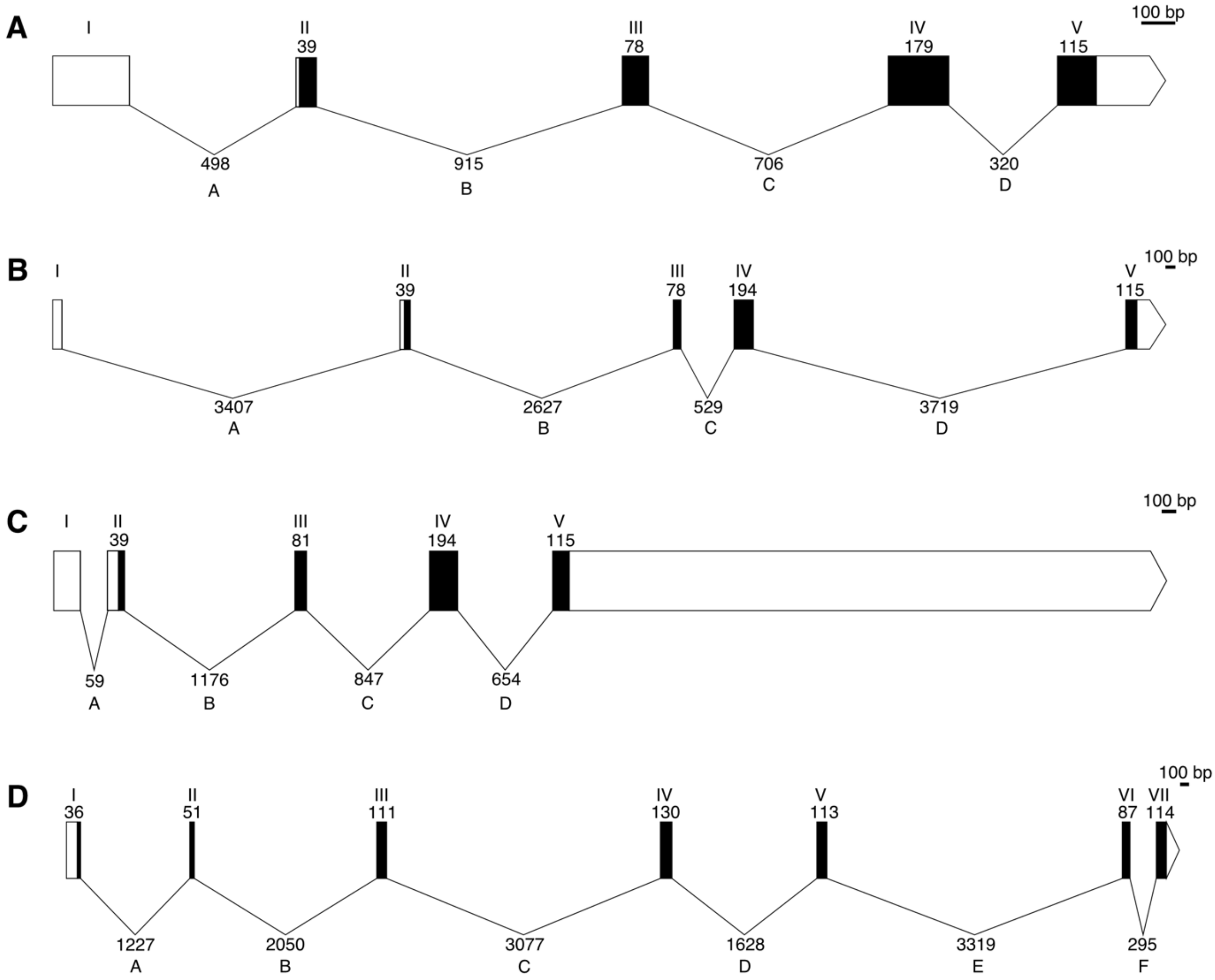

Appendix Fig. S1. Structures of the human genes for the ATP synthase c-subunit and the OSCP. (*A*), *ATP5MC1*, (*B*), *ATP5MC2*, (*C*), *ATP5MC3* and (*D*), *ATP5PO* encoding, respectively, three different precursors of the c-subunit and the OSCP. Removal of each of the three different N-terminal import sequences produces an identical mature c-subunit protein. Roman numerals denote exons; black and unfilled areas in exons represent, respectively, protein coding and non-coding regions; intervening continuous lines between exons represent introns, labelled with capital letters. The sizes of introns and coding regions of exons are given in base pairs (bp). On the right, the scale bars represent 100 bp. The exon-intron information was obtained from http://www.ensembl.org. The structures of the genes correspond to transcript ID references ENST00000393366.7, ENST00000394349.9, ENST0000028472.9 and ENST00000290299.7. Images were drawn with the Exon-Intron graphic maker (http://wormweb.org/exonintron).

```
A
                ^^^____________________
        WT CCTTCCTACAGCAACTTCCCACTCCAGGTGGCCAGACGGGAGTTCCAGACCAGTGTTGTC
        Δc CCTTCCTACA--------------------------------------------------

        WT TCCCGGGACATTGACACAGCAGCCAAGTTTATTGGTGCTGGGGCAGCCACAGTTGGTGTG
        Δc ------------------------------------------------------------
                                    ^^^____________________
        WT GCTGGTTCAGGGGCTGGCATTGGAACCGTGTTTGGCAGCTTGATCATTGGCTATGCCAGG
        Δc ------------------------------TTTGGCAGCTTGATCATTGGCTATGCCAGG

      WT 1 MQTAGALFISPALIRCCTRGLIRPVSASFLNSPVNSSKQPSYSNFPLQVARREFQTSVVS  60
      Δc 1 MQTAGALFISPALIRCCTRGLIRPVSASFLNSPVNSSKQPSYIWQLDHWLCQEPVSQAAA  60
                                                     ********** *******
           ┌→mature
     WT 61 RDIDTAAKFIGAGAATVGVAGSGAGIGTVFGSLIIGYARNPSLKQQLFSYAILGFALSEA 120
     Δc 61 LLLCHSWLCPV-------------------------------------------------  71
           ***********

    WT 121 MGLFCLMVAFLILFAM 136
    Δc  71 ----------------  71

B
               ^^^____________________
        WT CAAACCAGCGCCATTTCAAGGGACATCGACACAGCAGCCAAGTTCATTGGAGCTGGGGCT
        Δc CAAACCAG----------------------------------------------------
                                       ____________________^^^
        WT GCCACAGTTGGGGTGGCTGGTTCTGGGGCTGGGATTGGAACTGTGTTTGGGAGCCTCATC
        Δc ---------------------------------------------TTTGGGAGCCTCATC

      WT 1 MFACSKFVSTPSLVKSTSQLLSRPLSAVVLKRPEILTDESLSSLAVSCPLTSLVSSRSFQ  60
      Δc 1 MFACSKFVSTPSLVKSTSQLLSRPLSAVVLKRPEILTDESLSSLAVSCPLTSLVSSRSFQ  60
                 ┌→mature
     WT 61 TSAISRDIDTAAKFIGAGAATVGVAGSGAGIGTVFGSLIIGYARNPSLKQQLFSYAILGF 120
     Δc 61 TSLGASSLVMPGTLL---------------------------------------------  75
             *************

    WT 121 ALSEAMGLFCLMVAFLILFAM 141
    Δc  75 ---------------------  75

C
                          ^^^____________________
        WT TGTGTCTCAGCTAATCCAAAGGGAGTTTCAGACCAGTGCAATCAGCAGAGACATTGATAC
        Δc TGTGTCTCAGCTAATCCAAAG---------------------------------------
            ____________________^^^
        WT TGCTGCCAAATTTATTGGTGCAGGTGCTGCAACAGTAGGAGTGGCTGGTTCTGGTGCTGG
        Δc ----------------GGTGCAGGTGCTGCAACAGTAGGAGTGGCTGGTTCTGGTGCTGG

      WT 1 MFACAKLACTPSLIRAGSRVAYRPISASVLSRPEASRTGEGSTVFNGAQNGVSQLIQREF  60
      Δc 1 MFACAKLACTPSLIRAGSRVAYRPISASVLSRPEASRTGEGSTVFNGAQNGVSQLIQRVQ  60
                                                                     **
                  ┌→mature
     WT 61 QTSAISRDIDTAAKFIGAGAATVGVAGSGAGIGTVFGSLIIGYARNPSLKQQLFSYAILG 120
     Δc 61 VLQQ--------------------------------------------------------  64
           ****

    WT 121 FALSEAMGLFCLMVAFLILFAM 142
    Δc  64 ----------------------  64

D
               ^^^____________________
        WT CTTCCCAGAGAACCTAGCGGTTACGCCAACGCGCGCGTGCGCCCTTGCGCGTTTCTCTCT
     ΔOSCP CTTCCCA-----------------------------------------------------
                                                 ┌→
        WT TCCCACTCGGGTTTGACCTACAGCCGCCCGGGAGAAGATGGCTGCCCCAGCAGTGTCCGG
     ΔOSCP ------------------------------------------------------------

        WT GCTCTCCCGGCAGGTGAGAGAAAGGTGGTCCTGAGAGCCAGTGGGAGGATCCCTCCTGGG
     ΔOSCP ------------------------------------------------------------
                 ____________________^^^
        WT ATGACCGGTACCAAACCTCGAGCACCAGGGTGCGATGCTTCGGCTGGGGAAGTCTGGCAG
     ΔOSCP ------------------------CCAGGGTGCGATGCTTCGGCTGGGGAAGTCTGGCAG
```

Appendix Fig. S2. Sequences of the three genes encoding the c-subunit and of the gene encoding the OSCP subunit of h-ATP synthase compared with the disrupted genes in HEK293-Δ(c+OSCP) cells, and comparison of the wild-type and disrupted proteins. The DNA sequences are compared to the corresponding WT sequences for (*A*) *ATP5MC1*, (*B*) *ATP5MC2*, (*C*) *ATP5MC3*, and (*D*) *ATP5PO. ATP5MC1, ATP5MC2* and *ATP5MC3* encode different mitochondrial import sequences of the c-subunit, followed in each case by an identical sequence for the mature protein; *ATP5PO* encodes the OSCP subunit. Carets indicate the PAM (protospacer adjacent motif) sequences for each guide RNA, and solid lines the target sequences for guide RNAs. The DNA sequences show sections of WT intron sequence (grey box) and the targeted exon, aligned with the corresponding edited sequence. For gene editing, exon I was targeted in *ATP5PO*, and exon IV in *ATP5MC1, 2* and *3*. The horizontal arrows indicate the translational initiator methionine codons. In (*A-C*), the WT protein sequence of the precursor of the c-subunit (lower part) is aligned with the potential translated sequence from the disrupted gene, and the arrow indicates the N-terminal amino acid of the mature subunit. Deleted DNA and protein regions are denoted by dashed lines, and asterisks indicate non-matched amino acids. For gRNA sequences and PCR primer sequences of the genes, see Appendix Tables S6 and S7.

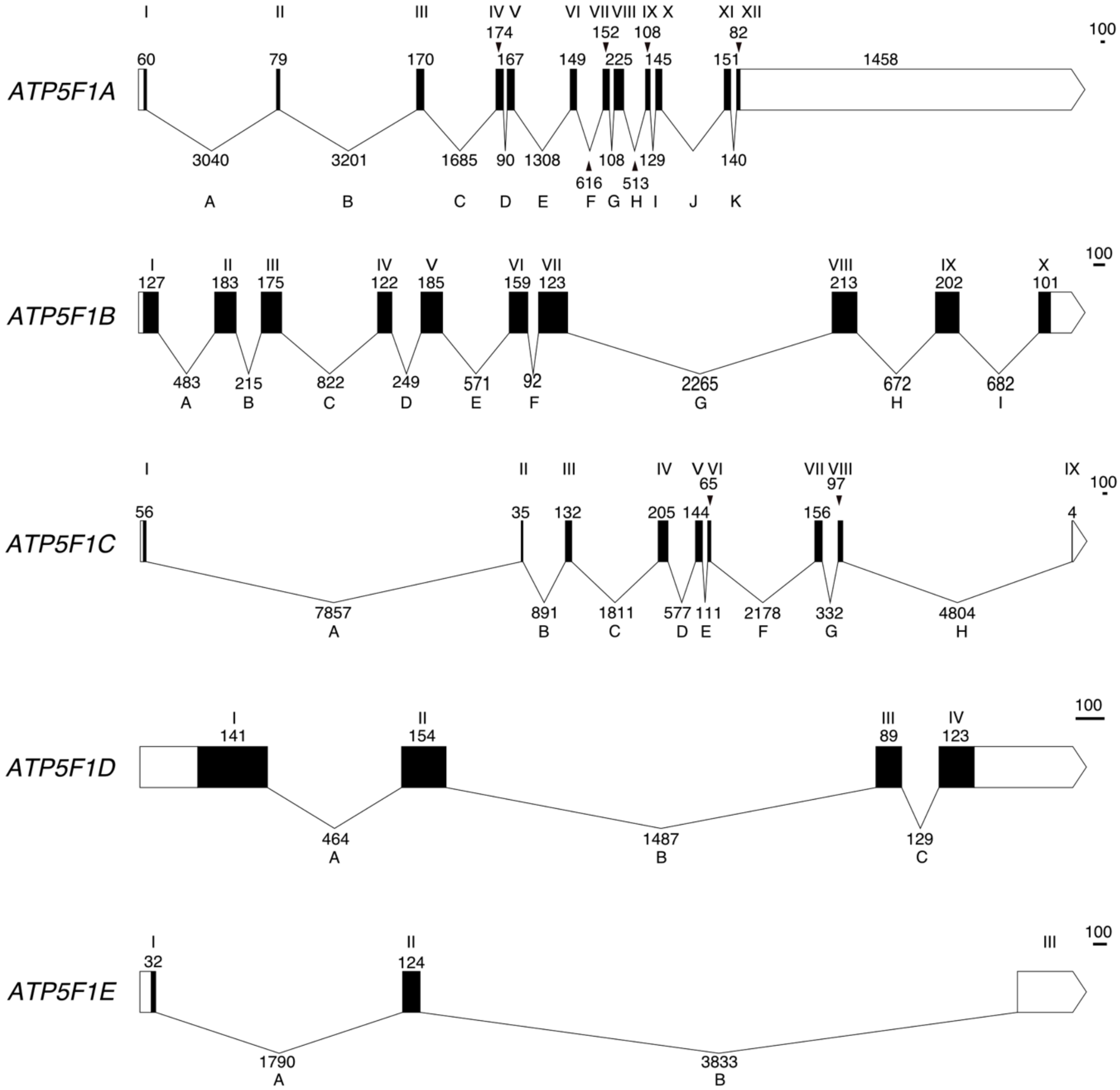

Appendix Fig. S3. Structures of the human genes *ATP5F1A, ATP5F1B, ATP5F1C ATP5F1D and ATP5F1E* encoding the α-, β-, γ-, δ- and ε-subunits, respectively, of the F_1-module of ATP synthase. Roman numerals denote exons; black and unfilled areas in exons represent, respectively, protein coding and non-coding regions; intervening continuous lines between exons represent introns, labelled with capital letters. The sizes of introns and coding regions of exons are given in base pairs (bp). On the right, the scale bars represent 100 bp. The exon-intron information was obtained from http://www.ensembl.org. The structures of the genes correspond to transcript ID references ENST00000398752.11, ENST00000262030.8, ENST00000335698.4, ENST00000215375.7 and ENST00000395663.1. Images were drawn with the Exon-Intron graphic maker (http://wormweb.org/exonintron).

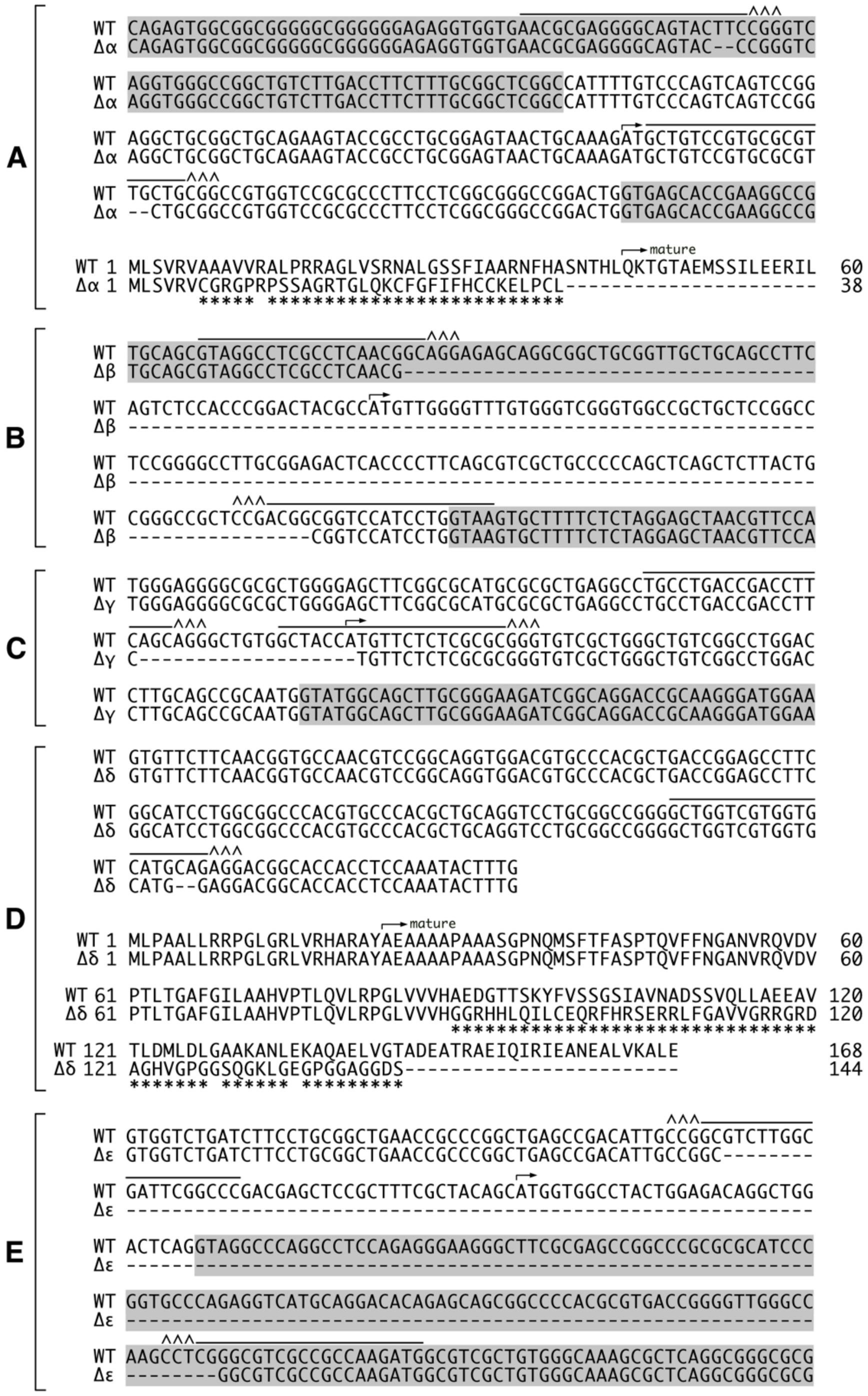

Appendix Fig. S4. Sequences of *ATP5F1A*, *ATP5F1B*, *ATP5F1C*, *ATP5F1D* and *ATP5F1E* encoding the five subunits of the F_1-module of h-ATP synthase compared with the disrupted genes, and a comparison of the wild-type and disrupted proteins. The DNA sequences are compared with the corresponding wild-type (WT) sequences for (*A*) *ATP5F1A*, (*B*) *ATP5F1B*, (*C*) *ATP5F1C*, (*D*) *ATP5F1D* and (*E*) *ATP5F1E*, encoding subunits α, β, γ, δ and ε, respectively. Carets indicate the PAM (protospacer adjacent motif) sequences for each guide RNA, and solid lines the target sequences for guide RNAs. The DNA sequences show sections of WT intron sequence (grey box) and the targeted exon, aligned with the corresponding edited sequence. Exon I was targeted, except for *ATP5F1D* where exon II was edited. The horizontal arrows indicate the translational initiator methionine codon of each gene. The WT protein sequence (lower part) for *ATP5F1A* (partial sequence) and *ATP5F1D* (full sequence), is aligned with the potential translated sequence from the disrupted gene, and the N-terminus of the mature subunit is indicated. Deleted DNA and protein regions are denoted by dashed lines, and asterisks indicate non-matching amino acids.

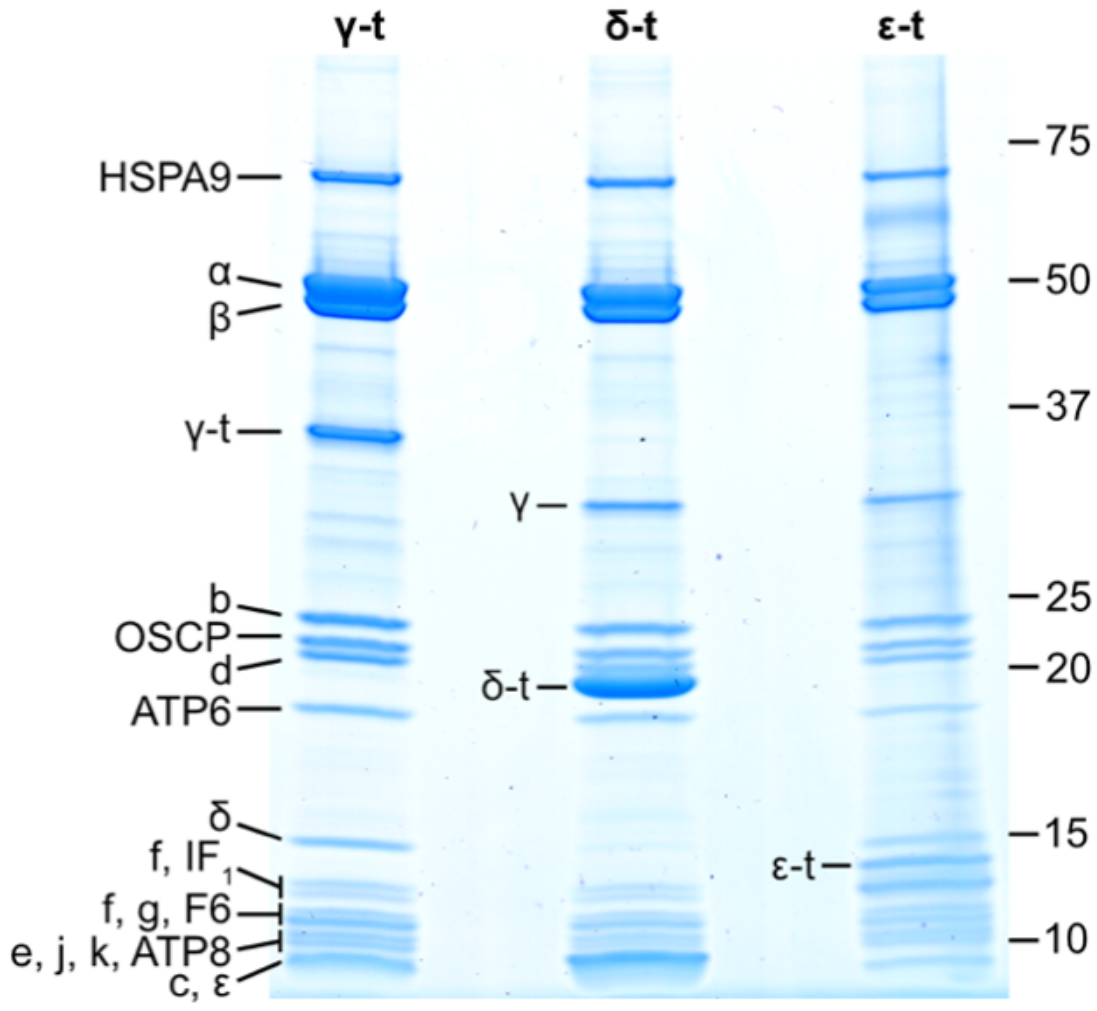

Appendix Fig. S5. Integration of tagged subunits γ-t, δ-t and ε-t into h-ATP synthase. Analysis by SDS-PAGE of ATP synthases captured with γ-t, δ-t and ε-t. Proteins were detected with Coomassie blue dye. The locations of subunits are indicated on the left, based on known subunit migration positions and, except for subunit c, tandem-MS of trypsin digests of stained gel bands in γ-t (see Appendix Table S2). HSPA9 is the mitochondrial 70 kDa heat shock protein (mtHsp70). Tagged subunits were expressed in HEK293-WT cells for 3 days, and digitonin extracts of mitochondria (detergent:protein, 10:1, g:g) were applied to a Strep II affinity column. Bound ATP synthase complexes were released with D-desthiobiotin.

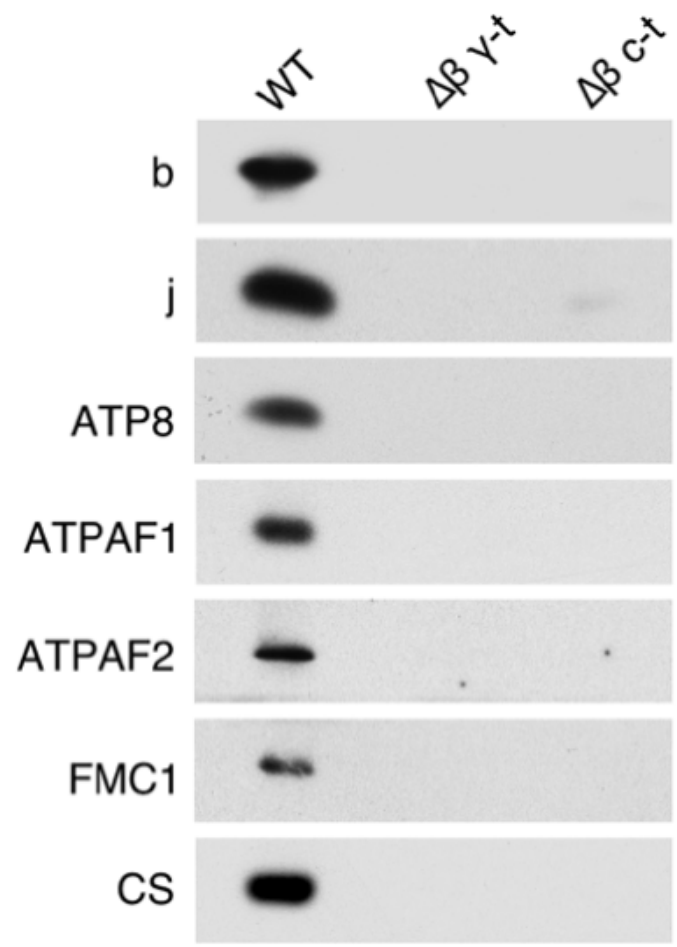

Appendix Fig S6. Failure of assembly factors ATPAF1, ATPAF2 and FMC1 to bind to tagged subunits γ-t and c-t of h-ATP synthase. γ-t and c-t were expressed in HEK293-Δβ cells, and mitochondria were extracted with DDM. Tagged subunits, together with any associated proteins, were purified from the extracts by Strep II affinity chromatography, fractionated by SDS-PAGE, and probed by western blotting with antibodies against the proteins indicated on the left. CS, citrate synthase loading control.

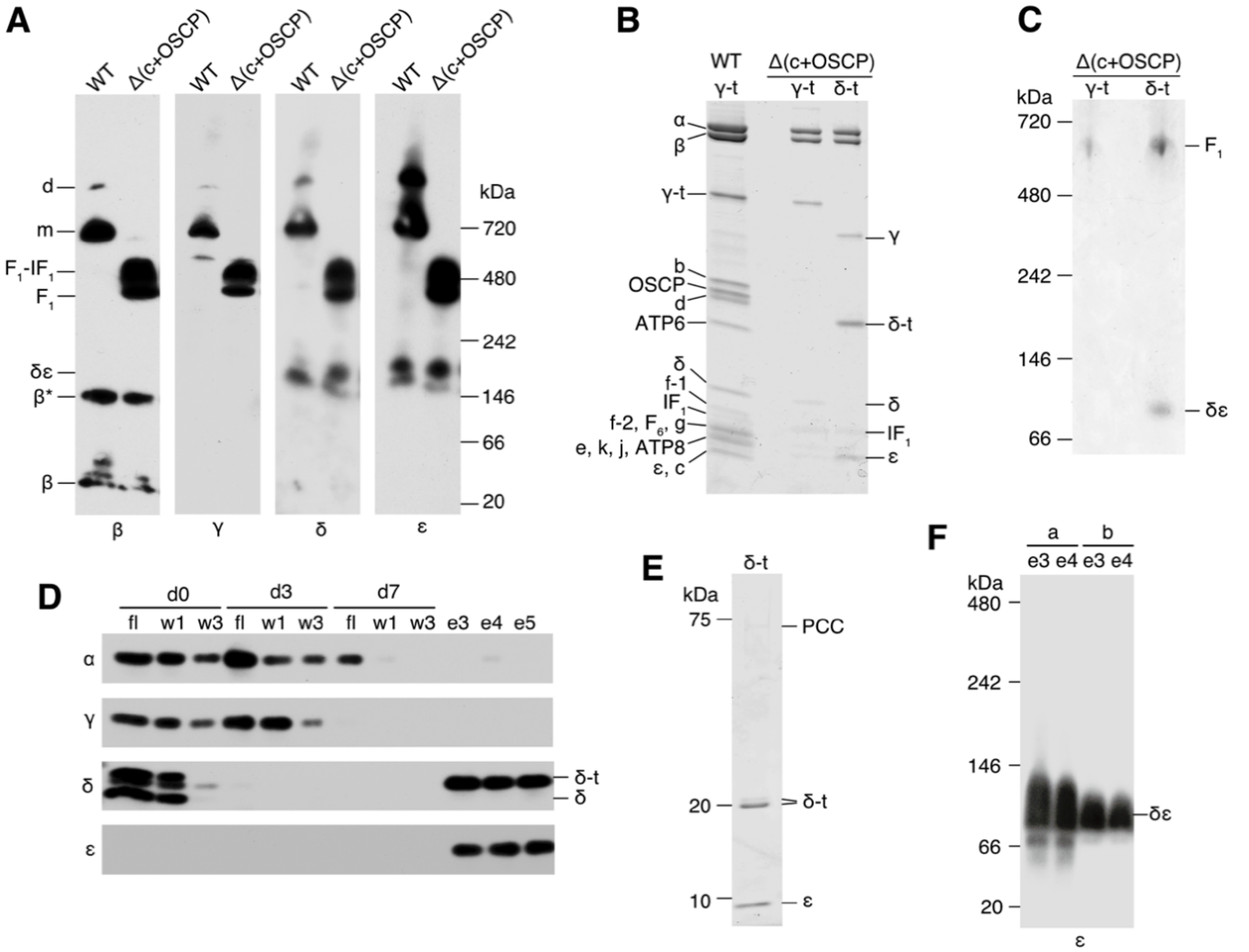

Appendix Fig. S7. ATP synthase and complexes in HEK293-WT and HEK293-Δ(c+OSCP) cells. (*A*) CN-PAGE analysis of digitonin extracts (10 g/g protein) of mitoplasts containing the same quantities of mitoplast proteins, and detection of proteins by western blotting with antibodies against subunits indicated beneath the panels; d and m, dimeric and monomeric ATP synthase, respectively; δε, complex of δ- and ε-subunits; β*, β-ATPAF1 complex; β, β-subunit. The positions of molecular weight markers are shown on the right; (*B*) SDS-PAGE analysis of affinity purified complexes containing γ-t or δ-t subunits expressed in HEK293-Δ(c+OSCP) cells. Mitochondria were lysed with DDM. Proteins were detected with Coomassie blue dye. The positions of ATP synthase subunits are indicated on the left; (*C*) CN-PAGE analysis of affinity purified complexes from HEK293-Δ(c+OSCP) cells in (*B*). The positions of protein molecular weight markers are indicated on the left; (*D*) cold stability of the δε-complex. The δ-t-ε complex from HEK293-Δ(c+OSCP) cells was kept bound to Strep II agarose beads, at 4°C for 7 days and then after 0, 3 and 7 days, the beads were washed and eluted with desthiobiotin. The presence of F_1-subunits was monitored by western blotting with antibodies against the subunits indicated on the left of the panels. The bands in the initial flow-through and w1 arise from endogenous and tagged δ-subunits. The cold-labile F_1-domain was disrupted during the incubation period and the α-, γ- and δ-subunits had dissociated by day 3, leaving the bound δε-complex, which was eluted at day 7. fl, flow through; w1 and w3, first and third washes; e3-e5, eluates 3 to 5; (*E*) SDS-PAGE analysis of eluted δε-complexes from (*D*) and staining with Coomassie blue dye. Proteins in stained bands were analyzed by tandem-MS of in-gel tryptic digests of bands, and are labelled on the right. PCC, propionyl-CoA carboxylase (contaminant binding to Strep-Tactin agarose) (see Appendix Table S3). The identities of proteins were confirmed by intact mass measurement by LC-MS (Appendix Table S4). The faint band above the δ-subunit had arisen by chemical modification (Appendix Table S4). (*F*) CN-PAGE analysis of δ-t-ε complexes in eluates e3 and e4 purified from HEK293-Δ(c+OSCP) cells in (*D*), and detection by western blotting with an ε-subunit antibody. The positions of protein molecular weight markers are shown on the left. a, δ-t purified complexes from (*D*), but stored at -20°C; b, freshly purified δε complexes from (*D*); e3 and e4, purification eluate samples 3 and 4.

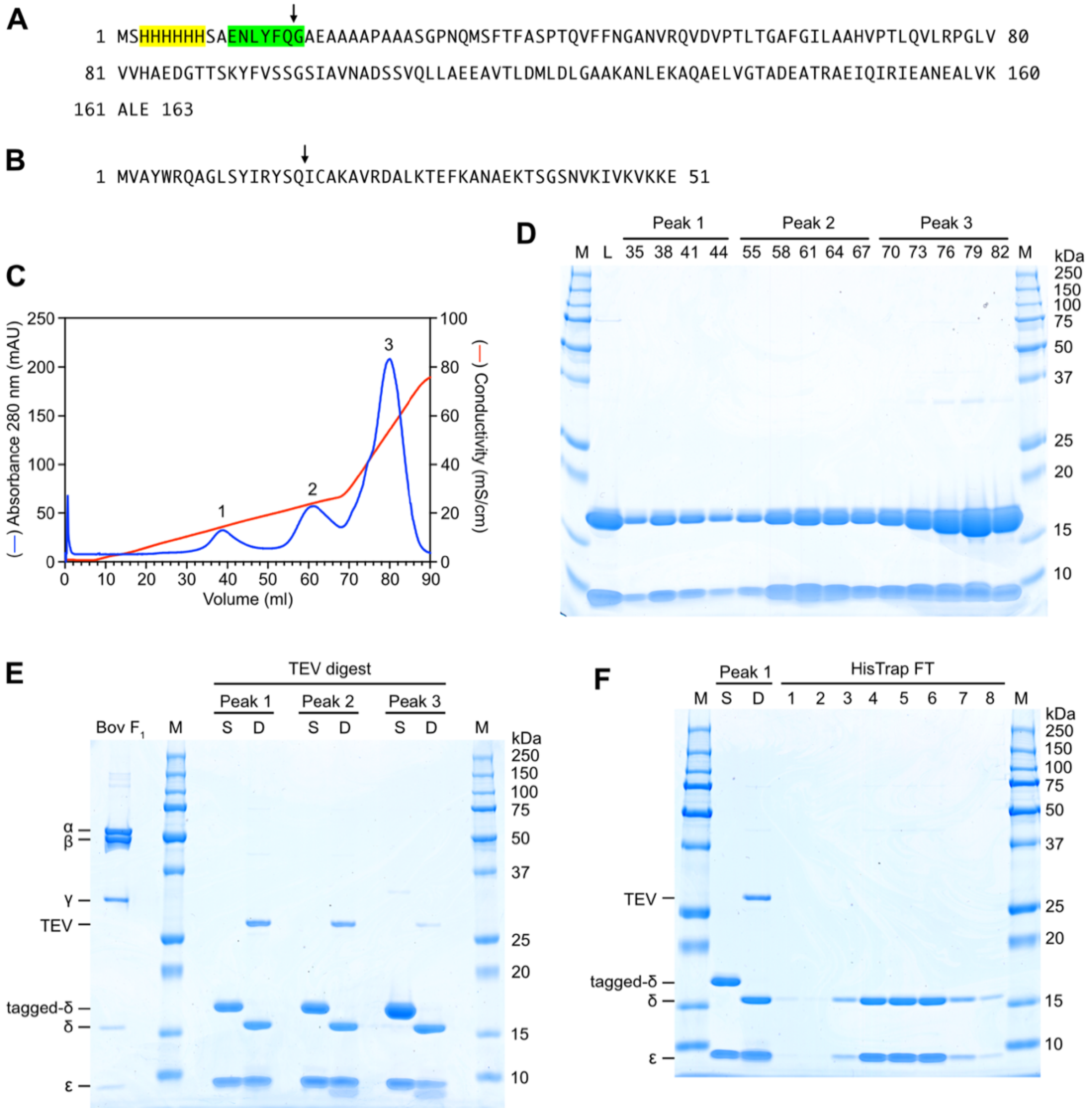

Appendix Fig. S8. Purification and characterization of recombinant human δε-complex. (*A*) and (*B*), amino acid sequences, respectively, of the δ-subunit with an N-terminal His-TEV tag, and of the ε-subunit. In (A), the 6xHis tag is yellow, and TEV protease recognition sequence is green. TEV-cleavage at the vertical arrow releases the mature δ-subunit with an additional N-terminal glycine residue. In (*B*), the vertical arrow indicates a potential intrinsic TEV protease cleavage site. (*C*), Fractionation of recombinant δε-complex on a HiTrap SP cation exchange column; fractions of 1 ml were collected; (*D*), SDS-PAGE analysis of fractions from (*C*). Proteins were stained with Coomassie blue dye. L, unfractionated load material. M, molecular weight markers (kDa). (*E*), SDS-PAGE analysis of the products of TEV-cleavage of the tagged δ-subunit in peaks 1-3 in (*C*), stained with Coomassie blue dye. S and D, respectively, material before and after digestion. Bov F_1, bovine F_1-ATPase. The positions of subunits and molecular weight standards (M; kDa) are indicated on the left and right, respectively. In peaks 2 and 3, the ε-subunit is partially degraded by digestion with TEV. (*F*) SDS-PAGE analysis of the δε-complex in peak 1. Before analysis, the material was digested with TEV protease, and passed through a HisTrap column to remove the His tagged TEV which binds to the column; S and D, undigested and digested material; HisTrap FT, flow-through fractions from the HisTrap column; M, molecular weight markers (kDa). The molecular masses of the purified δ and ε components were confirmed by intact mass measurement by LC-MS (Appendix Table S4).

```
α-subunit    1 QKTGTAEMSSILEERILGADTSVDLEETGRVLSIGDGIARVHGLRNVQAEEMVEFSSGLK  60
β-subunit    1 AQTS---------------PSPKAGAATGRIVAVIGA------------VVDVQFDEGLP  33
                :*.                :      ***:::: ..               *:*..**

α-subunit   61 GM-----------------SLNLEPDNVGVVVFGNDKLIKEGDIVKRTGAIVDVPVGEEL 103
β-subunit   34 PILNALEVQGRETRLVLEVAQHLGESTVRTIAMDGTEGLVRGQKVLDSGAPIKIPVGPET  93
                :                 : :*  ..* .:.:.. : : .*: *  :** :..:*** *

α-subunit  104 LGRVVDALGNAIDGKGPIGSKTRRRVGLKAPGIIPRISVREPMQTGIKAVDSLVPIGRGQ 163
β-subunit   94 LGRIMNVIGEPIDERGPIKTKQFAPIHAEAPEFMEMSVEQEILVTGIKVVDLLAPYAKGG 153
               ***:::.:*: ** :*** :*    :  :** ::     :* : ****.** *.* .:*

α-subunit  164 RELIIGDRQTGKTSIAIDTIINQKRFNDGSDEKKKLYCIYVAIGQKRSTVAQLVKRLTDA 223
β-subunit  154 KIGLFGGAGVGKTVLIMELINNVAKAH-------GGYSVFAGVGERTREGNDLYHEMIES 206
               :  ::*.  .*** : :: * *  : :         *.::..:*::     :* :.: ::

α-subunit  224 DAMKYT------IVVSATASDAAPLQYLAPYSGCSMGEYFRDNGKHALIIY-DDLSKQAV 276
β-subunit  207 GVINLKDATSKVALVYGQMNEPPGARARVALTGLTVAEYFRDQEGQDVLLFIDNIFRFTQ 266
               ..:: .       :* .  .:    :  .  :* ::.*****:  : :::: *:: : :

α-subunit  277 AYRQMSLLLRRPPGREAYPGDVFYLHSRLLERAAKMNDAFGGGSLTALPVIETQAGDVSA 336
β-subunit  267 AGSEVSALLGRIPSAVGYQPTLATDMGTMQERIT----TTKKGSITSVQAIYVPADDLTD 322
               *  ::* ** * *.  .*   :    . : ** :    :   **:*:: .* . *.*::

α-subunit  337 YIPTNVISITDGQIFLETELFYKGIRPAINVGLSVSRVGSAAQT-RAMKQVAGTMKLELA 395
β-subunit  323 PAPATTFAHLDATTVLSRAIAELGIYPAVDPLDSTSRIMDPNIVGSEHYDVARGVQKILQ 382
                 *:..::  *.  .*.  :   ** **::   *.**: .   .     :**  ::  *

α-subunit  396 QYREVAAF-AQFGSDL-DAATQQLLSRGVRLTELLKQGQYSPMAIEEQVAVIYAGVRGYL 453
β-subunit  383 DYKSLQDIIAILGMDELSEEDKLTVSRARKIQRFLSQ--------PFQVAEVFTGHMGKL 434
               :*:.:  : * :* *  .   :  :**. :: .:*.*          *** :::*  * *

α-subunit  454 DKLEPSKITKFENAF---LSHVVSQHQALLGTIRA----DGKISEQSDAKLKEIVTNFLA 506
β-subunit  435 VPLKE-TIKGFQQILAGEYDHLPEQAFYMVGPIEEAVAKADKLAEEHSS----------- 482
                 *:  .*. *:: :    .*: .*   ::* *.      .*::*: .:

α-subunit  507 GFEA  510
β-subunit  482 ----  482
```

Appendix Fig. S9. Alignment of the sequences of the α- and β-subunits of h-ATP synthase. The sequences (UniProt: P25705 and P06576) were aligned with Clustal Omega (1.2.4) (Sievers et al., 2011) and the regions of secondary structure were taken from the structures of the bovine orthologs (UniProt: P19483 and P00829; PDBs 1W0K, 6YY0 and 4Z1M). Residues with the symbols *, **:** and **.** are identical, strongly conserved, and weakly conserved, respectively. 19.9% of residues are identical and a further 20.6% substituted conservatively. Grey and cyan denote α-helices and β-strands, respectively.

```
A
     WT    1  MAAVVVAAAGGAGPAVLQVAGLYRGLCAVRSRALGLGLVSPAQLRVFPVRPGSGRPEGGA   60
ΔATPAF1    1  MAAVVVAAAGGAGPAVLQVAGLYRGLCAVRSRALGLGLVSPAQLRVFPVRPGSGRPEGGA   60

     WT   61  DSSGVGAEAELQANPFYDRYRDKIQLLRRSDPAAFESRLEKRSEFRKQPVGHSRQGDFIK  120
ΔATPAF1   61  DSSGVGAEAELQANPFYDRYRDKIQLLRRSDPAAFESRLEKRSEFRKQPVGHSRQGDFIK  120

     WT  121  CVEQKTDALGKQSVNRGFTKDKTLSSIFNIEMVKEKTAEEIKQIWQQYFAAKDTVYAVIP  180
ΔATPAF1  121  CVEQKTDALGKQSVNRGFTKDKTLSSIFNIEMVKEKTAEEIKQIWQQYFAAKDTVYAVIP  180

     WT  181  AEKFDLIWNRAQSCPTFLCALPRREGYEFFVGQWTGTELHFTALINIQTRGEAAASQLIL  240
ΔATPAF1  181  AEKFDLIKPGSVLSNISMCSAKKGRL----------------------------------  206
                     *******************
     WT  241  YHYPELKEEKGIVLMTAEMDSTFLNVAEAQCIANQVQLFYATDRKETYGLVETFNLRPNE  300
ΔATPAF1  206  ------------------------------------------------------------  206

     WT  301  FKYMSVIAELEQSGLGAELKCAQNQNKT 328
ΔATPAF1  206  ---------------------------- 206

B
     WT    1  MWRSCLRLRDGGRRLLNRPAGGPSASMSPGPTIPSPARAYAPPTERKRFYQNVSITQGEG   60
ΔATPAF2    1  MWRSCLRLRDGGRRLLNRPAGGPSASMSPGPTIPSPADRKEEVLSECQHHTG--------   52
                                                   ***************
     WT   61  GFEINLDHRKLKTPQAKLFTVPSEALAIAVATEWDSQQDTIKYYTMHLTTLCNTSLDNPT  120
ΔATPAF2   52  ------------------------------------------------------------   52

     WT  121  QRNKDQLIRAAVKFLDTDTICYRVEEPETLVELQRNEWDPIIEWAEKRYGVEISSSTSIM  180
ΔATPAF1   52  ------------------------------------------------------------   52

     WT  181  GPSIPAKTREVLVSHLASYNTWALQGIEFVAAQLKSMVLTLGLIDLRLTVEQAVLLSRLE  240
ΔATPAF2   52  ------------------------------------------------------------   52

     WT  241  EEYQIQKWGNIEWAHDYELQELRARTAAGTLFIHLCSESTTVKHKLLKE   289
ΔATPAF2   52  -------------------------------------------------    52

C
     WT    1  MAALGSPSHTFRGLLRELRYLSAATGRPYRDTAAYRYLVKAFRAHRVTSEKLCRAQHELH   60
  ΔFMC1    1  MAALGSPSHTFRGLLRELRYLSAATGRPYRDTAAYRYLVKAFRASGHQ------------   48
                                                          ****
     WT   61  FQAATYLCLLRSIRKHVALHQEFHGKGERSVEESAGLVGLKLPHQPGGKGWEP  113
  ΔFMC1   48  -----------------------------------------------------   48
```

Appendix Fig. S10. Altered protein sequences arising from the editing of *ATPAF1*, *ATPAF2* and *FMC1* in HAP1 cells. Comparison of the WT protein sequence with those of the three vestigial assembly factors of ATP synthase in (*A*) HAP1-ΔATPAF1, (*B*) HAP1-ΔATPAF2 and (*C*) HAP1-ΔFMC1. Asterisks indicate unmatched amino acids, and dashed lines indicate the deleted sequence. Arrows indicate the N-terminal amino acid of the mature subunits. The mature FMC1 lacks methionine-1 and residue alanine-2 is acetylated.

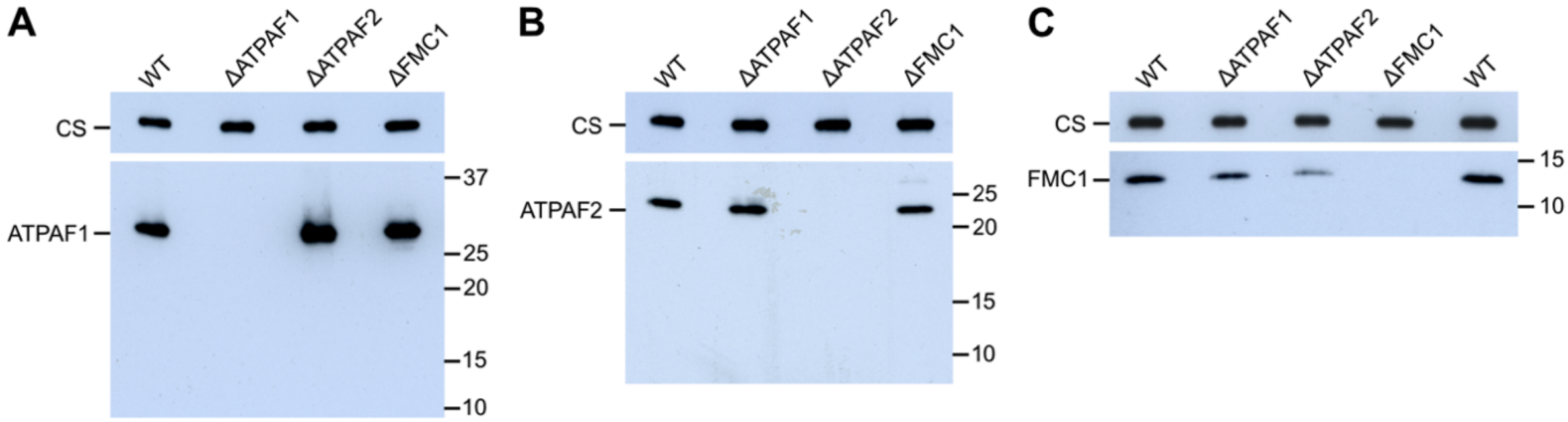

Appendix Fig. S11. Impact of individual deletion of assembly factors for the F_1-domain of h-ATP synthase from HAP1 cells on the other assembly factors. (*A*)-(*C*), detection by western blotting with antibodies against ATPAF1, ATPAF2 and FMC1, respectively, of SDS-PAGE analyses of dodecylmaltoside extracts of mitoplasts from HAP1-WT, -ΔATPAF1, -ΔATPAF2 and -ΔFMC1 cells; CS, citrate synthase loading controls. The positions of molecular weight markers (kDa) are indicated on the right.

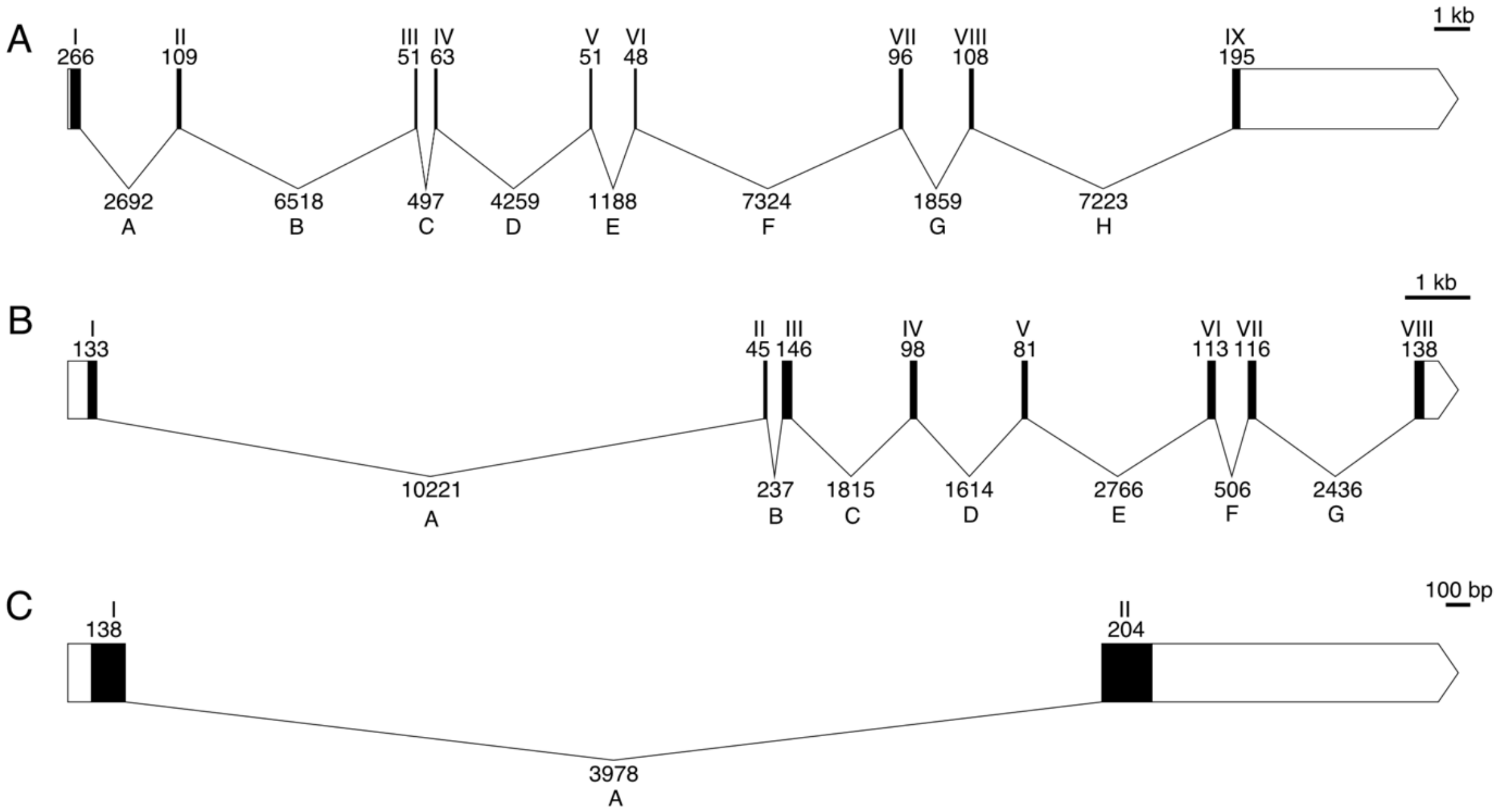

Appendix Fig. S12. Structures of the human genes encoding assembly factors for the F_1-module of ATP synthase. (*A*), *ATPAF1*, (*B*), *ATPAF2*, and (*C*) *FMC1*. Roman numerals denote exons; black and unfilled areas in exons represent, respectively, protein coding and non-coding regions; intervening continuous lines between exons represent introns, labelled with capital letters. The sizes of introns and coding regions of exons are given in base pairs (bp). On the right, the scale bars represent three DNA sizes. The exon-intron information was obtained from http://www.ensembl.org. The structures of the genes correspond to transcript ID references ENST00000574428.6, ENST00000474627.8 and ENST00000297534.7. Images were drawn with the Exon-Intron graphic maker (http://wormweb.org/exonintron).

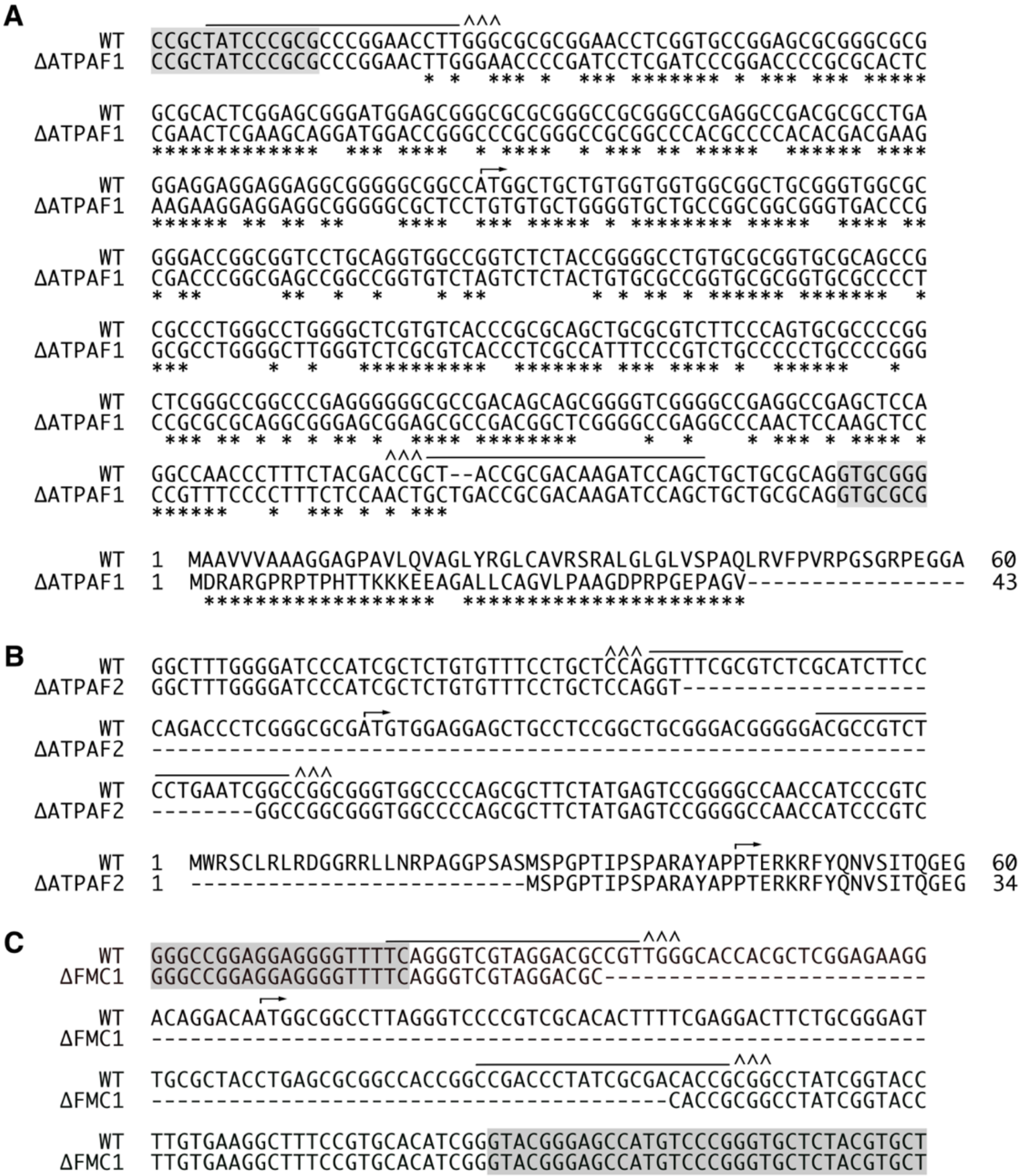

Appendix Fig. S13. Sequences of the genes encoding the assembly factors for the F_1-module of h-ATP synthase disrupted together in HEK293-WT cells. The DNA sequences are compared to the corresponding wild-type (WT) sequence for (*A*) *ATPAF1, (B) ATPAF2* and (*C*) *FMC1*, encoding the assembly factors ATPAF1, ATPAF2 and FMC1, respectively. Carets indicate the PAM (protospacer adjacent motif) sequences for each guide RNA, and solid lines the target sequences for guide RNAs. The DNA sequences show sections of WT intron sequence (grey box) and the targeted exon, aligned with the corresponding edited sequence, asterisks indicate the unmatched sequences to those in WT; and extra bases in the edited section of ATPAF1 are denoted by adding dash lines to the WT sequence. The target exons are all exon I, and the initiation codons are removed in the edited cells. In ΔATPAF1, a random DNA sequence has been inserted between the two double strand breaks. FMC1 is no longer produced in the edited cells. The horizontal arrows indicate the protein initiator methionine codon of each gene. In the lower part of (*A),* a possible protein sequence produced from an inserted ATG codon is aligned with amino acids 1-60 of the WT sequence. In ΔATPAF2, a second in-frame ATG codon remaining after the deletion in exon I, could lead to the production of a protein 26 amino acids shorter than the WT protein. This change could impede the import of the protein into mitochondria, but no protein was detected (Fig. EV6). The arrow indicates the N-terminus of the mature WT protein. In the edited sequences, deletions in the DNA and encoded proteins are denoted by dashed lines.

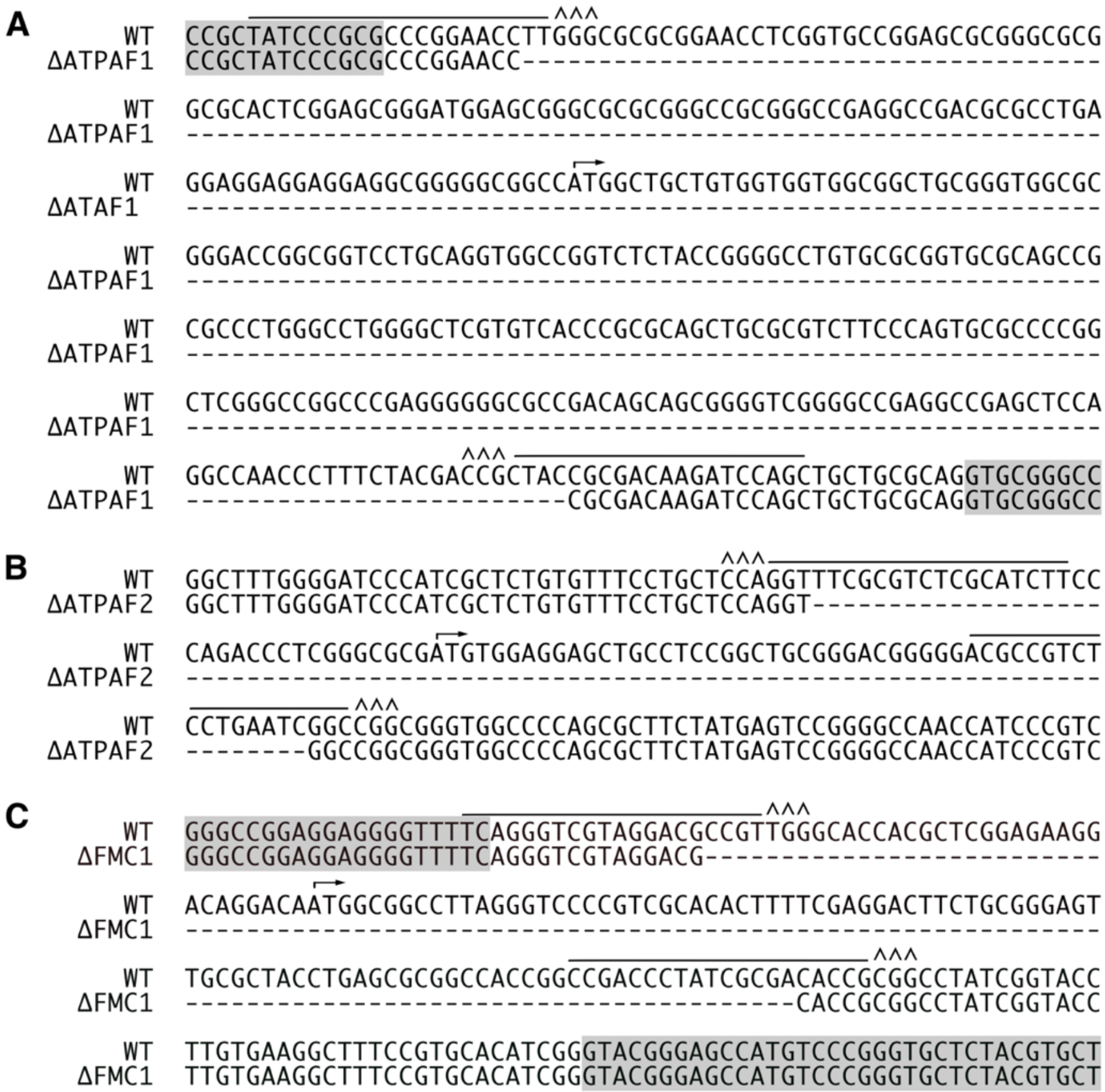

Appendix Fig. S14. Sequences of the genes encoding the three assembly factors for the F_1-module of h-ATP synthase in HEK293-Δ(c+OSCP) cells compared with the individually disrupted genes. The DNA sequences are compared to the corresponding wild-type (WT) sequence for (*A*) *ATPAF1*, (*B*) *ATPAF2* and *(C) FMC1*. Carets indicate the PAM sequences for each guide RNA, and solid lines the target sequences for guide RNAs. The DNA sequences show sections of WT intron sequence (grey box) and the targeted exon, aligned with the corresponding edited sequence. In all three cases, the deletion was targeted to exon I. Translational initiation codons, denoted by horizontal arrows, were removed in all three edited cell lines. A second in-frame initiation codon remains in ΔATPAF2 allowing the possibility of a translation product truncated at its N-terminal end by 26 amino acids (Appendix Fig. S13), but no such protein was detected (Fig. EV7; Appendix Fig. S17). Deleted DNA regions are denoted by dashed lines.

```
A
     WT   CCGCTATCCCGCGCCCGGAACCTTGGGCGCGCGGAACCTCGGTGCCGGAGCGCGGGCGCG
ΔATPAF1   CCGCTATCCCGCGCCCGGAACC--------------------------------------

     WT   GCGCACTCGGAGCGGGATGGAGCGGGCGCGCGGGCCGCGGGCCGAGGCCGACGCGCCTGA
ΔATPAF1   ------------------------------------------------------------

     WT   GGAGGAGGAGGAGGCGGGGGCGGCCATGGCTGCTGTGGTGGTGGCGGCTGCGGGTGGCGC
ΔATAF1    ------------------------------------------------------------

     WT   GGGACCGGCGGTCCTGCAGGTGGCCGGTCTCTACCGGGGCCTGTGCGCGGTGCGCAGCCG
ΔATPAF1   ------------------------------------------------------------

     WT   CGCCCTGGGCCTGGGGCTCGTGTCACCCGCGCAGCTGCGCGTCTTCCCAGTGCGCCCCGG
ΔATPAF1   ------------------------------------------------------------

     WT   CTCGGGCCGGCCCGAGGGGGGCGCCGACAGCAGCGGGGTCGGGGCCGAGGCCGAGCTCCA
ΔATPAF1   ------------------------------------------------------------

     WT   GGCCAACCCTTTCTACGACCGCTACCGCGACAAGATCCAGCTGCTGCGCAGGTGCGGCC
ΔATPAF1   -------------------------CGCGACAAGATCCAGCTGCTGCGCAGGTGCGGCC

B
     WT   GGCTTTGGGGATCCCATCGCTCTGTGTTTCCTGCTCCAGGTTTCGCGTCTCGCATCTTCC
ΔATPAF2   GGCTTTGGGGATCCCATCGCTCTGTGTTTCCTGCTCCAGGT-------------------

     WT   CAGACCCTCGGGCGCGATGTGGAGGAGCTGCCTCCGGCTGCGGGACGGGGGACGCCGTCT
ΔATPAF2   ------------------------------------------------------------

     WT   CCTGAATCGGCCGGCGGGTGGCCCCAGCGCTTCTATGAGTCCGGGGCCAACCATCCCGTC
ΔATPAF2   --------GGCCGGCGGGTGGCCCCAGCGCTTCTATGAGTCCGGGGCCAACCATCCCGTC
```

Appendix Fig. S15. Sequences of the genes encoding assembly factors ATPAF1 and ATPAF2 for the F_1-module of h-ATP synthase, disrupted together in HEK293-Δ(c+OSCP) cells. The DNA sequences are compared to the corresponding wild-type (WT) sequence for (*A*) *ATPAF1* and (*B*) *ATPAF2*, respectively. Carets indicate the PAM (protospacer adjacent motif) sequences for each guide RNA, and solid lines the target sequences for guide RNAs. The DNA sequences show sections of WT intron sequence (grey box) and the targeted exon, aligned with the corresponding edited sequence. The target exons are all exon I, and the initial codons are removed in the edited cells. The presence of a second in-frame ATG codon in ΔATPAF2 could lead to a protein truncated at its N-terminal end by 26 amino acids (see legend to Appendix Fig. S13). Both ATPAF1 and ATPAF2 are no longer produced in the edited cells (Fig. EV7; Appendix Fig. S17). The horizontal arrows indicate the protein initiator methionine codon of each gene. Deleted DNA and protein regions are denoted by dashed lines.

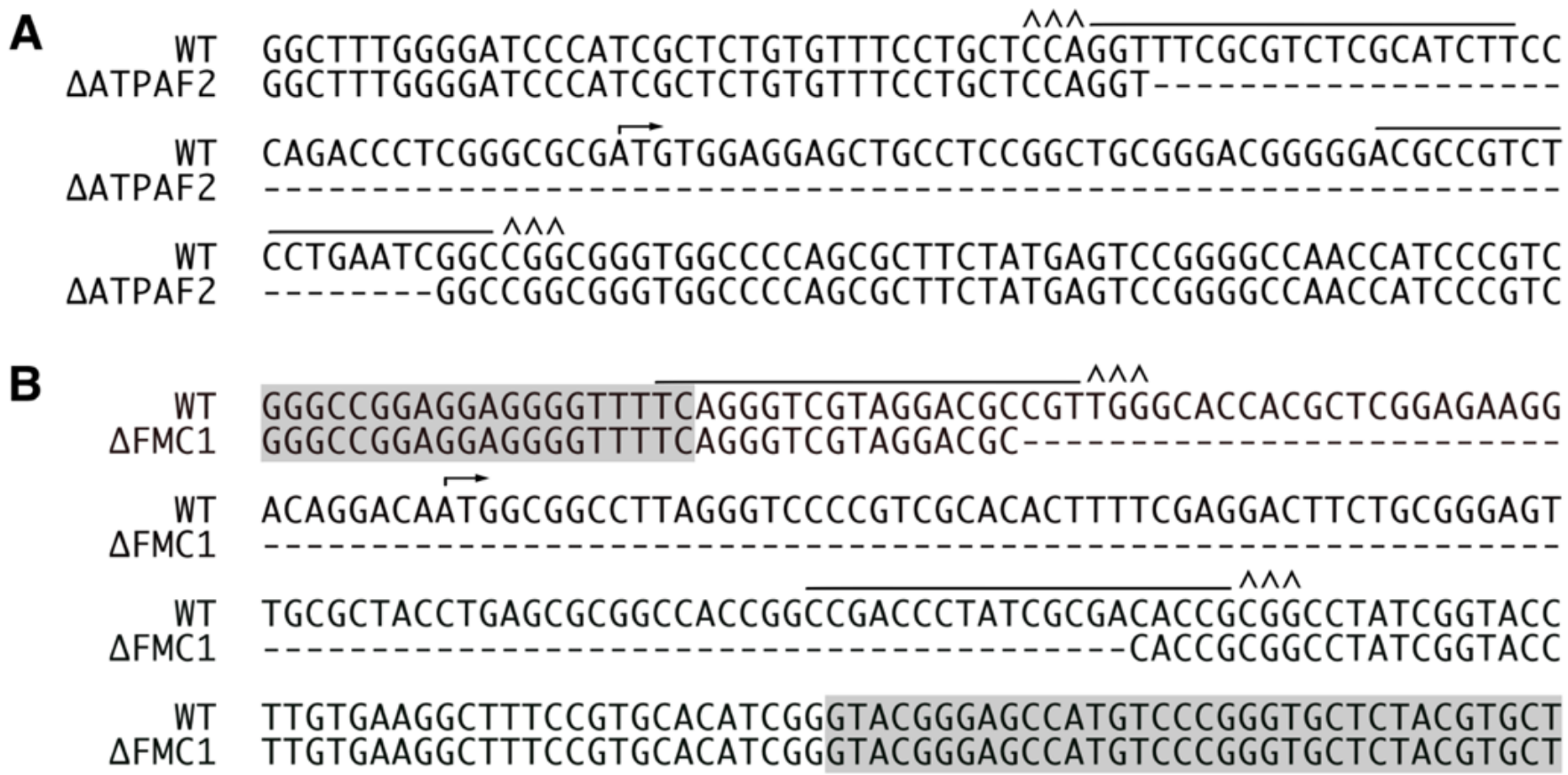

Appendix Fig. S16. Sequences of *ATPAF2* and *FMC1* each encoding an assembly factor for the F_1-module of h-ATP synthase disrupted together in HEK293-Δ(c+OSCP) cells. The DNA sequences are compared to the corresponding wild-type (WT) sequences for (*A*) *ATPAF2* and (*B*) *FMC1*, encoding the assembly factors ATPAF2 and FMC1, respectively. Carets indicate the PAM (protospacer adjacent motif) sequences for each guide RNA, and solid lines the target sequences for guide RNAs. The DNA sequences show sections of WT intron sequence (grey box) and the targeted exon, aligned with the corresponding edited sequence. The target exons are all exon I, and the initial codons are removed in the edited cells. A second in-frame ATG codon in ΔATPAF2 could lead to a protein truncated at its N-terminal end by 26 amino acids (see legend to Appendix, Fig. S13). Both ATPAF2 and FMC1 are no longer produced in the edited cells (Fig. EV7; Appendix Fig. S17). The horizontal arrows indicate the protein initiator methionine codon of each gene. Deleted DNA and protein regions are denoted by dashed lines.

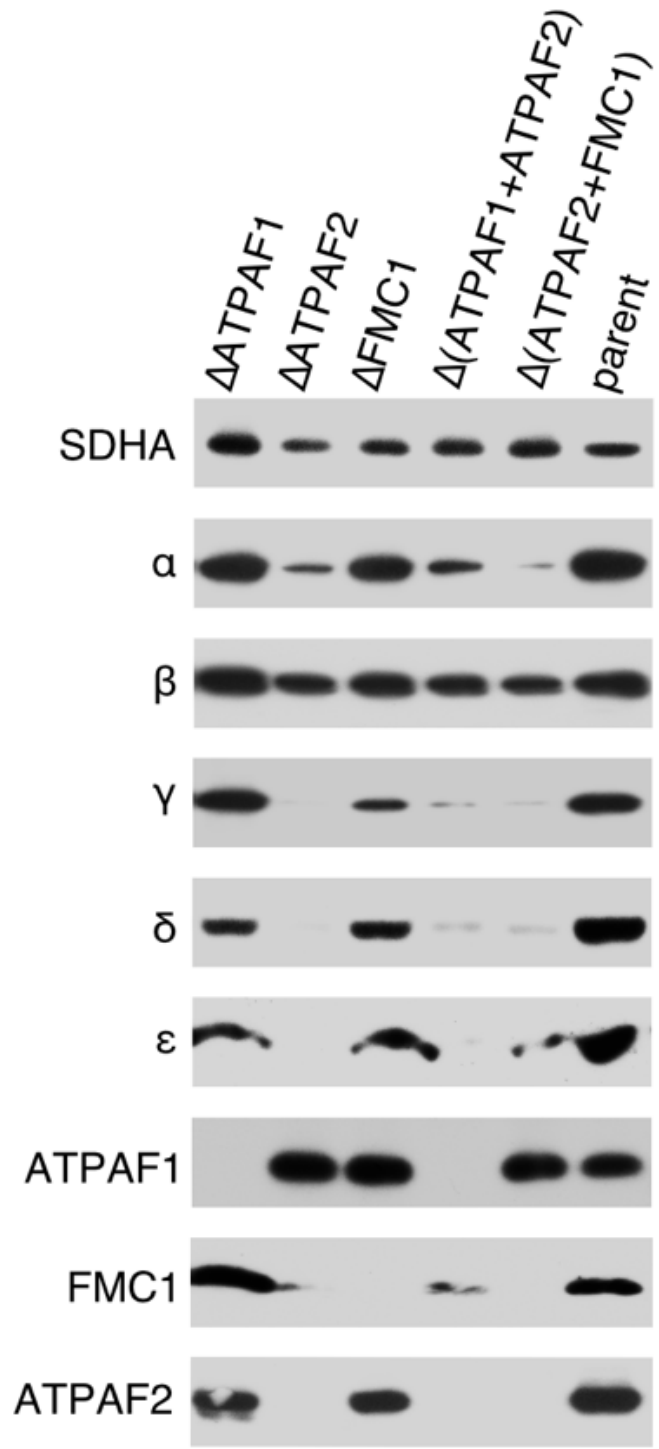

Appendix Fig. S17. Impact of the removal of individual or pairs of assembly factors for the F_1-module of h-ATP synthase from 'parent' HEK293-Δ(c+OSCP) cells. HEK293-Δ(c+OSCP)-ΔATPAF1, -ΔATPAF2, -ΔFMC1, -Δ(ATPAF1+ATPAF2) and -Δ(ATPAF2+FMC1) cells were generated. Shown are western blots employing the antibodies with the specificities indicated on the left of DDM extracts of mitoplasts from the cells, fractionated by SDS-PAGE. SDHA, flavoprotein subunit A from succinate dehydrogenase, was employed as a loading control.

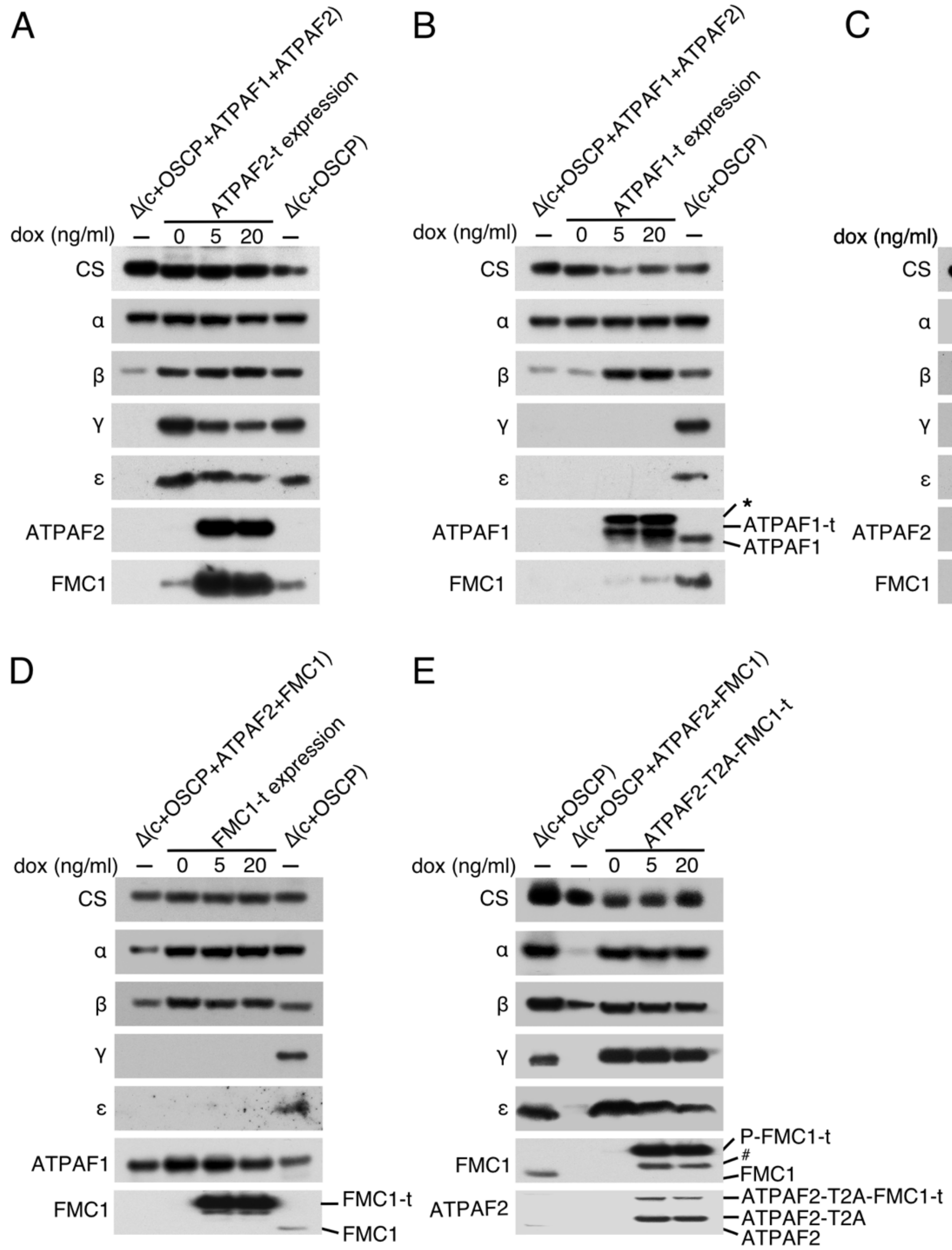

Appendix Fig. S18. Recovery of expression of subunits of the F_1-module of h-ATP synthase by re-introduction of assembly factors into assembly factor deficient cells. SDS-PAGE analyses and western blotting of DDM extracts of (*A*) and (*B*), HEK293-Δ(c+OSCP+ATPAF1+ATPAF2) cells expressing ATPAF2-t and ATPAF1-t, respectively, and (*C-E*), HEK293-Δ(c+OSCP+ATPAF2+FMC1) cells expressing ATPAF2-t, FMC1-t and ATPAF2-T2A-FMC1-t, respectively; the suffix "-t" denotes the presence of a C-terminal tandem FLAG-Strep II tag. Also, in (*A-E*) are shown analyses of a DDM extract of HEK293-Δ(c+OSCP) cells denoted as Δ(c+OSCP). The specificities of the antibodies are indicated on the left of each panel. CS, citrate synthase loading control. Expression of tagged proteins was induced for 3 days with doxycycline (dox; 0, 5 and 20 ng/ml). In (*B*), two forms of ATPAF1-t were detected, the authentic protein and a precursor form, denoted by an asterisk. In (*E*), the expected products ATPAF2-T2A and P-FMC1-t predominate (Appendix Fig. S19). #, denotes a probable proteolytic degradation product of P-FMC1-t, and ATPAF2-T2A-FMC1-t arises from ribosome skipping (Appendix Fig. S19). The δ-subunit was not detected for technical reasons.

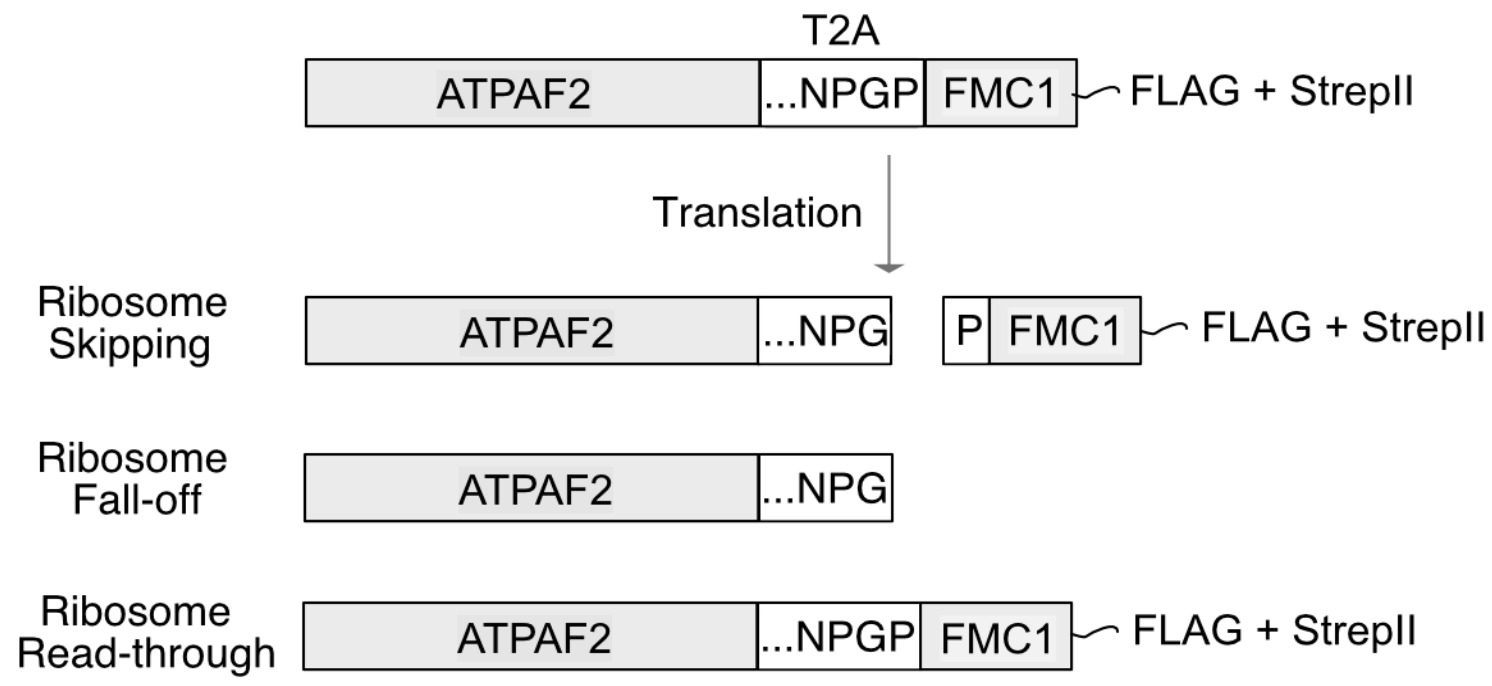

Appendix Fig. S19. The fusion construct for expression of ATPAF2 and FMC1-t. The coding sequences for ATPAF2 and FMC1-t (grey) were cloned with an intervening sequence encoding a 5'-T2A self-cleaving peptide (Liu et al., 2017) with an N-terminal GSG linker (GSGEGRGSLLTCGDVEENPGP) denoted as T2A. When translated, three possible products can arise, as shown. Ribosome skipping produces ATPAF2 with an additional C-terminal peptide NPG, plus FMC1 with a proline residue replacing the N-terminal methionine and with C-terminal FLAG and Strep II tags. Ribosome fall-off leads to ATPAF2 with an additional C-terminal peptide NPG only. Ribosome read-through generates a fusion protein consisting of ATPAF2 linked to tagged FMC by the 21 residue T2A peptide sequence shown above in parentheses. In practice, ribosome skipping predominated, and only low amounts of intact ATPAF2-T2A-FMC1-t were detected (Appendix Fig. S18E).

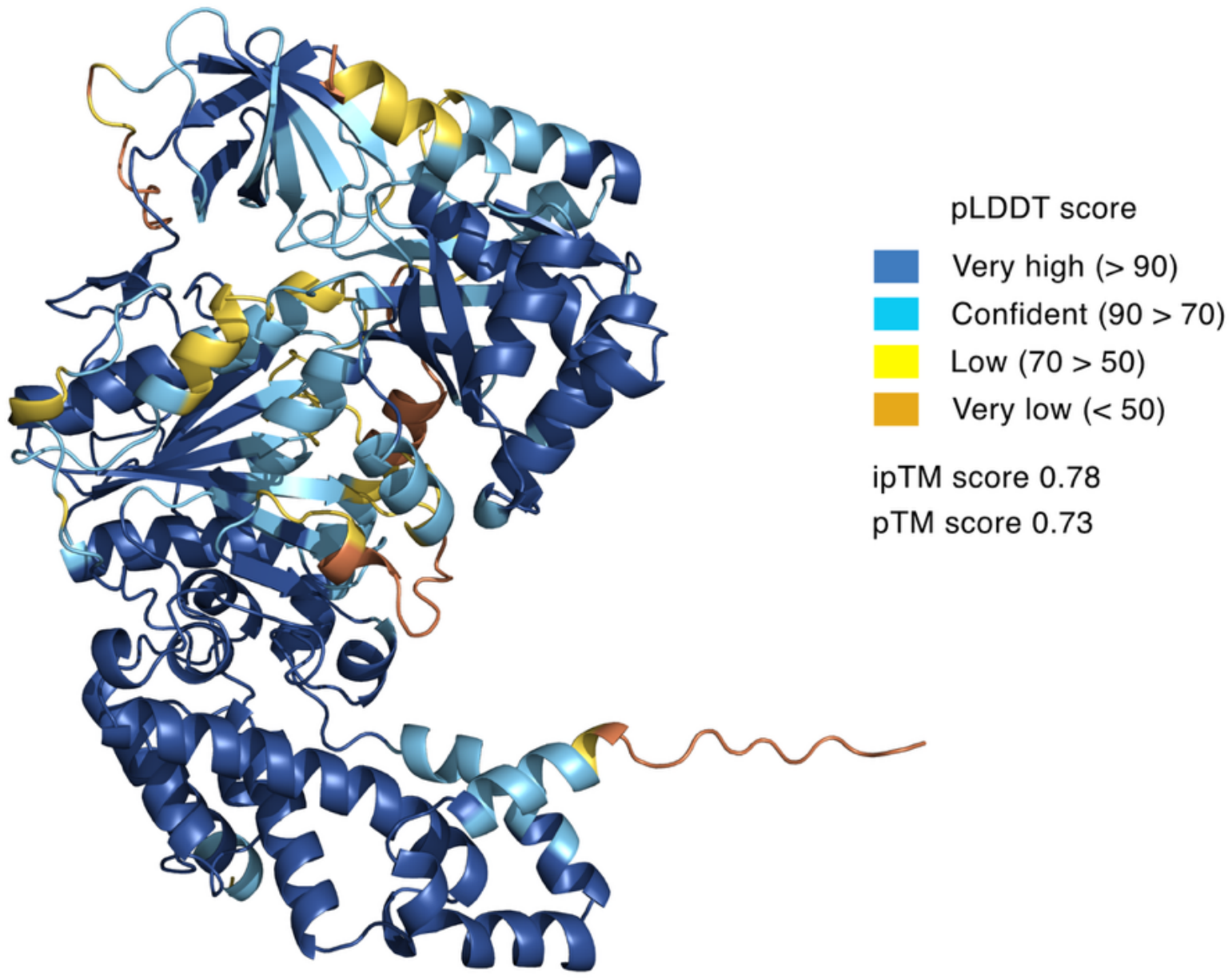

Human β-subunit

```
  1 AAQTSPSPKA GAATGRIVAV IGAVVDVQFD EGLPPILNAL EVQGRETRLV LEVAQHLGES  60
 61 TVRTIAMDGT EGLVRGQKVL DSGAPIKIPV GPETLGRIMN VIGEPIDERG PIKTKQFAPI 120
121 HAEAPEFMEM SVEQEILVTG IKVVDLLAPY AKGGKIGLFG GAGVGKTVLI MELINNVAKA 180
181 HGGYSVFAGV GERTREGNDL YHEMIESGVI NLKDATSKVA LVYGQMNEPP GARARVALTG 240
241 LTVAEYFRDQ EGQDVLLFID NIFRFTQAGS EVSALLGRIP SAVGYQPTLA TDMGTMQERI 300
301 TTTKKGSITS VQAIYVPADD LTDPAPATTF AHLDATTVLS RAIAELGIYP AVDPLDSTSR 360
361 IMDPNIVGSE HYDVARGVQK ILQDYKSLQD IIAILGMDEL SEEDKLTVSR ARKIQRFLSQ 420
421 PFQVAEVFTG HMGKLVPLKE TIKGFQQILA GEYDHLPEQA FYMVGPIEEA VAKADKLAEE 480
481 HSS
```

Human ATPAF1

```
  1 GSGRPEGGAD SSGVGAEAEL QANPFYDRYR DKIQLLRRSD PAAFESRLEK RSEFRKQPVG  60
 61 HSRQGDFIKC VEQKTDALGK QSVNRGFTKD KTLSSIFNIE MVKEKTAEEI KQIWQQYFAA 120
121 KDTVYAVIPA EKFDLIWNRA QSCPTFLCAL PRREGYEFFV GQWTGTELHF TALINIQTRG 180
181 EAAASQLILY HYPELKEEKG IVLMTAEMDS TFLNVAEAQC IANQVQLFYA TDRKETYGLV 240
241 ETFNLRPNEF KYMSVIAELE QSGLGAELKC AQNQNKT
```

Appendix Fig. S20. The confidence of prediction of the elements of the structure of the β-ATPAF1 complex. The structure was predicted by AlphaFold 3 from the mature protein sequences **(**β-subunit P06576, residues 47-529; ATPAF1 Q5TC12, residues 52-328). The colors correspond to the pLDDT confidence scores, and were generated in PyMol with the AlphaFold_coloring.py script (https://github.com/ailienamaggiolo/alphafold_coloring). Beneath the structure, the sequences of the β-subunit and ATPAF1 are colored according to the structure above. For reference, the complex depicted here is the same as shown in Figs. EV8A and EV9A, where the individual proteins are differentially colored.

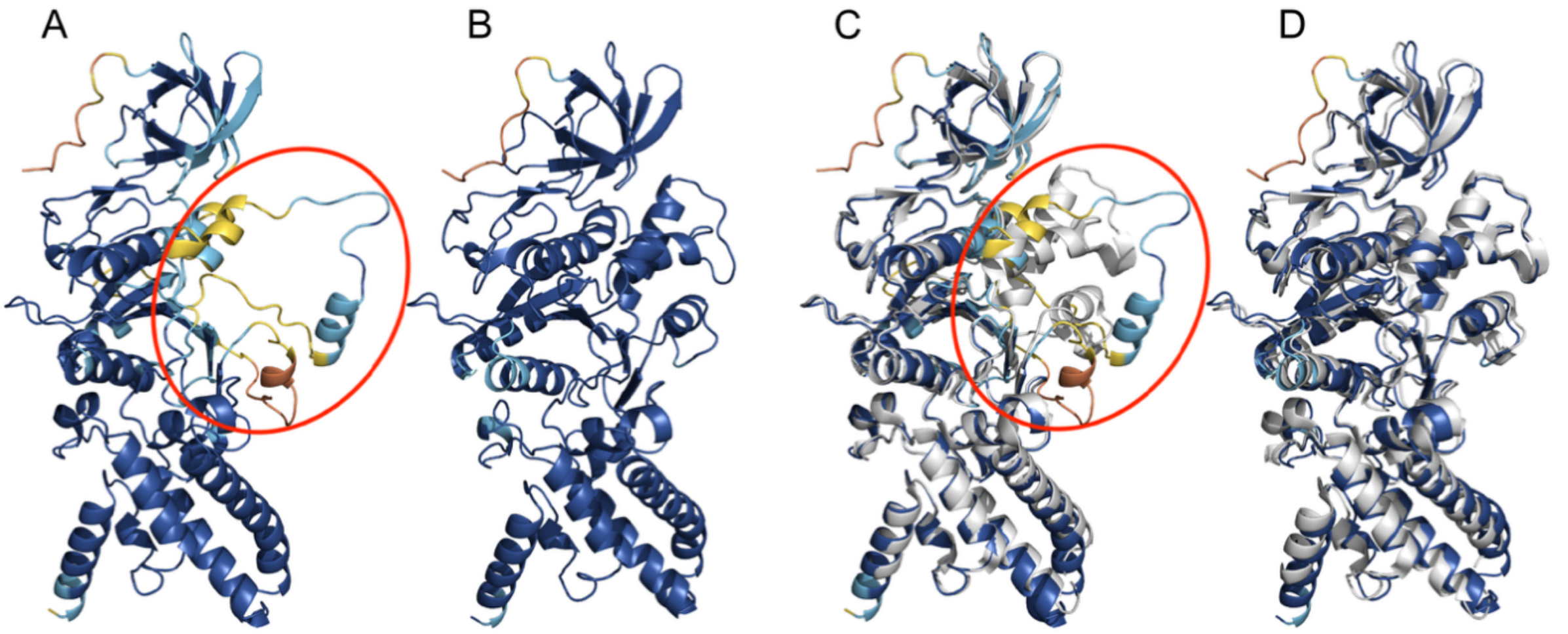

Appendix Fig. S21. Comparison of human β-subunit structures. (*A*), Structure produced for the β-subunit in an AlphaFold 3 prediction of the ATPAF1-β complex (Fig. EV8A). (*B*), AlphaFold 3 prediction for the β-subunit alone. The coloring of (A) and (B) reflects the pLDDT confidence score generated by AlphaFold 3 (Appendix Fig. S20). (*C* and *D*), Alignment of predicted structures (*A*) and (*B*), respectively, with the cryo-EM solved human β-empty subunit (*grey,* PDB code 8H9S chain G/E). The red ellipse highlights the area of lower confidence in the β-subunit prediction within the AlphaFold 3 produced ATPAF1-β complex.

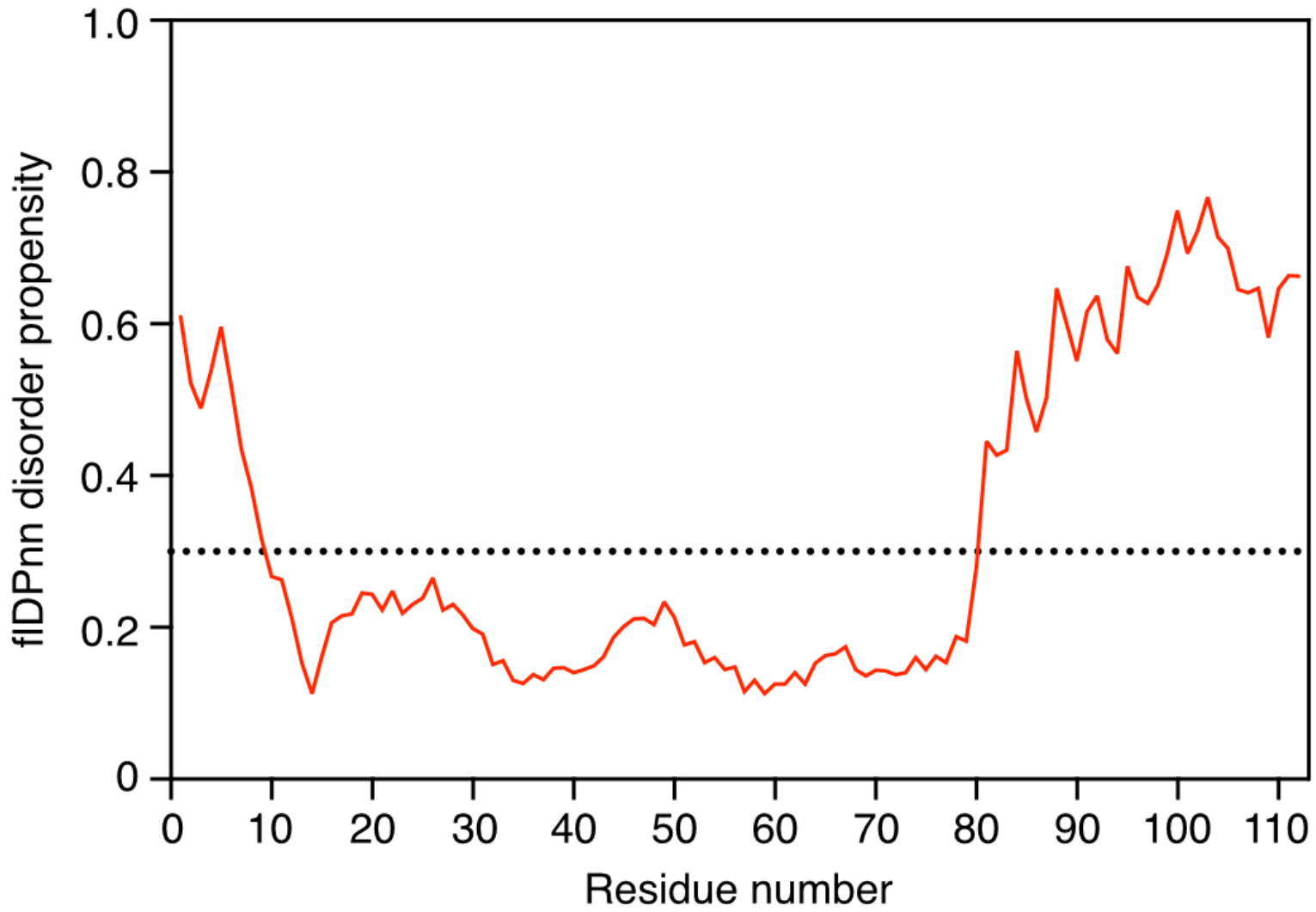

Appendix Fig. S22. Plot of the calculated disorder propensity for FMC1. A fast and local Disorder Prediction using neural networks (flDPnn; Hu et al., 2021) visualization of protein intrinsic disorder in FMC1. Values above the dotted line are predicted as disordered.

Appendix Table S1. Identification of ATP synthase subunits and assembly factors in F_1-ATPase module purified from HEK-Δ(c+OSCP) cells

Protein	Tandem MS data[1]					
(UniProt)	m/z	Mass (M)		Δmass	Sequence	Score[2]
		Observed	Calculated	(ppm)		
α	408.2347	814.4549	814.4548	0.02	ELIIGDR	54
(P25705)	423.2587	844.5028	844.5018	1.22	STVAQLVK	47
	430.7545	859.4944	859.4949	0.59	QMSLLLR	66
	446.7480	891.4814	891.4814	0.05	LELAQYR	54
	452.2336	902.4527	902.4531	-0.52	EPMQTGIK	48
	500.7930	999.5715	999.5713	0.22	VLSIGDGIAR	76
	501.3089	1000.6032	1000.6029	0.33	RSTVAQLVK	36
	510.7611	1019.5076	1019.5070	0.33	RSTVAQLVK	40
	513.8013	1025.588	1025.5869	1.02	AVDSLVPIGR	47
	575.3109	1148.6073	1148.6077	-0.4	GYLDKLEPSK	54
	586.3198	1170.6250	1170.6245	0.44	VVDALGNAIDGK	86
	644.3508	1286.6871	1286.687	0.06	HALIIYDDLSK	78
	658.8747	1315.7348	1315.7347	0.09	TDIAIDTIINQK	73
	712.3403	1422.6661	1422.6660	0.05	TGTAEMSSILEER	114
	480.288	1437.8421	1437.8416	0.33	GIRPAINVGLSVSR	39
	777.373	1552.7314	1552.7310	0.25	EAYPGDVFYLHSR	57
	788.3964	1574.7782	1574.7788	-0.39	ILGADTSVDLEETGR	132
	812.9489	1623.8833	1623.8832	-0.05	TGAIVDVPVGEELLGR	73
	567.9649	1700.8729	1700.8733	-0.26	DNGKHALIIYDDLSK	32
	1052.5223	2103.0301	2103.0307	-0.26	GMSLNLEPDNVGVVVFGNDK	75
β	450.2625	898.5104	898.5124	-2.2	VLDSGAPIK	61
(P06576)	488.2849	974.5551	974.5549	0.22	IGLFGGAGVGK	56
	519.8009	1037.5872	1037.5869	0.29	IPVGPETLGR	66
	544.8216	1087.6287	1087.6277	0.94	VVDLLAPYAK	57
	631.8238	1261.6330	1261.6336	-0.48	TIAMGDTEGLVR	80
	693.3592	1384.7039	1384.7020	-0.8	IMNVIGEPIDER	66
	703.8435	1405.6725	1405.6739	-1.00	AHGGYSVFAGVGER	84
	718.3810	1434.7475	1434.7467	0.58	FTQAGSEVSALLGR	112
	720.3983	1438.7821	1438.7820	0.05	VALTGLTVAEYFR	108
	729.4233	1456.832	1456.8323	-0.2	TVLIMELINNVAK	77
	801.4083	1600.8021	1600.8031	-0.66	VALVYGQMNEPPGAR	90
	825.9625	1649.9104	1649.9101	0.18	LVLEVAQHLGESTVR	93
	908.4373	1814.8601	1814.8621	-1.12	IMDPNIVGSEHYDVAR	102
	960.0525	1918.0904	1918.0888	0.87	VLDSGAPIKIPVGPETLGR	62
	961.4868	1920.959	1920.9581	0.43	DQEGQDVLLFIDNIFR	42
	994.5206	1987.0267	1987.0262	0.26	AIAELGIYPAVDPLDSTSR	78
	675.0081	2022.0025	2022.0033	-0.4	FLSQPFQVVAEVFTGHMGK	58

	1031.0035	2059.9925	2059.9884	1.99	EGNDLYHEMIESGVINLK	108
	1060.0277	2118.0409	2118.0402	0.33	SLQDIIAILGMDELSEEDK	68
	756.0326	2265.0761	2265.0770	-0.37	IPSAVGYQPTLATDMGTMQER	85
γ	432.7503	863.4860	863.4865	-0.59	LTLTFNR	40
(P36542)	454.2132	906.4118	906.4117	0.13	NASEMIDK	45
	524.2883	1046.562	1046.5608	1.16	SEVATLTAAGK	76
	530.789	1059.5634	1059.5634	0.02	EVMLVGIGDK	51
	548.8098	1095.6051	1095.6037	1.29	HLLIGVSSDR	71
	408.9068	1223.6987	1223.6986	0.04	KHLLIGVSSDR	32
	646.8353	1291.6565	1291.6561	-0.08	THSDQFLVAFK	55
	657.8606	1313.7066	1313.7078	0.88	ELIEIISGAAALD	29
	663.869	1325.7234	1325.7231	0.23	IYGLGSLALYEK	66
ATPAF1	443.2796	884.5446	884.5443	0.36	DKIQLLR	30
(Q5TC12)	482.2559	962.4982	962.4972	-0.17	FDLIWNR	36
	490.2279	978.4413	978.4407	0.68	SDPAAFESR	45
	568.2784	1134.5422	1134.5418	0.39	RSDPAAFESR	51
	603.3242	1204.6339	1204.6339	0.05	DTVYAVIPAEK	54
	641.8331	1281.6516	1281.6506	0.78	QIWQQYFAAK	27
	699.3710	1396.7274	1396.7272	0.14	TLSSIFNIEMVK	34
δ	493.7798	985.545	985.5444	0.64	IEANEALVK	52
(P30049)	716.3577	1430.7008	1430.7001	0.47	AQAELVGTADEATR	78
	714.7095	2141.1068	2141.1076	-0.4	AQAELVGTADEATRAEIQIR	30
FMC1	594.8272	1187.6399	1187.6398	0.10	SVEESAGLVGLK	62
(Q96HJ9)	627.8341	1253.6535	1253.6516	1.52	YLSAATGRPYR	28
	688.368	2002.0821	2002.0847	-1.31	SVEESAGLVGLKLPHQPGGK	4
IF_1	450.7792	899.5438	899.5440	-0.21	EQLAALKK	39
(P56381)	432.2146	1293.6219	1293.6214	0.36	HHEEEIVHHK	53
	655.7837	1309.5528	1309.5535	-0.5	GFGSDQSENVDR	82
	678.8129	1355.6109	1355.6106	0.47	EQAEEERYFR	37
	711.865	1421.7154	1421.7164	-0.66	HHEEEIVHHKK	39
	481.6035	1441.7886	1441.7888	-0.15	EIERLQKEIER	22
	517.6111	1549.8114	1549.8113	0.06	KHHEEEIVHHKK	32
	588.6154	1762.8244	1762.8234	0.59	EAGGAFGKREQAEEER	47
ε	421.2045	840.3945	840.3952	-0.86	MVAYWR	32
(P56381)	454.2521	906.4896	906.4923	-3.0	QAGLSIYR	51
	476.2611	1463.7632	1462.7620	0.47	DALKTEFK	37
	488.9283	1463.7632	1463.7620	0.84	DALKTEFKANAEK	49

[1] Tandem MS data from multiply charged peptide ions acquired in a Q-Exactive instrument were compared with MASCOT against a UniProt protein sequence database (v. August 2020) containing 20,453 human protein sequences (human proteins, trypsin constraints, variable modifications were Met oxidation and Cys propionamide, a maximum of two missed cleavages, peptide mass tolerance ± 5 ppm, fragment mass tolerance ± 0.01 Da).
[2]Individual MASCOT ions scores >17 indicate peptides with identity or extensive homology ($p<0.05$). Underlined methionine residues are modified by oxidation.

Appendix Table S2. Data concerning the MS identification of proteins associated with a tagged γ-subunit after SDS-PAGE separation.

Label[1]	1	2	3	4	5
HSPA9	HSPA9 (2; 14247)[2]	α (1; 16951)	β (4; 3720)		
α	α (1; 44685)	β (2; 14035)	γ (6; 545)		
β	β (1; 44952)	α (2; 9893)	γ (6; 733)		
γ	γ (1; 9503)	α (3; 827)	β (5; 770)	b (6; 755)	
b	b (1; 10849)	OSCP (2; 1620)	α (12; 298)		
OSCP	OSCP (1; 7099)	b (2; 842)	d (3; 704)		
d	d (1; 2757)	OSCP (2; 1315)	α (3; 793)		
ATP6	ATP6 (2; 614)	α (4; 416)	δ (5; 369)	γ (7;349)	
δ	δ (1; 3970)	α (3, 556)	g (7; 272)		
f, IF_1	g (1; 1179)	f (3; 845)	IF_1 (5; 546)	α (6; 5450	e (8; 485)
f, g, F_6	g (; 5098)	F_6 (2, 3170)	e (3; 2613)	k (4; 1660)	f (6; 975)
e, k, j, ATP8	e (1;5368)	k (3; 703)	ATP8 (4; 508)	g (5; 415)	j (6, 274)
ε, c	e (2; 865)	ε (3; 503)	α (5; 273)	ATP8 (6;235)	j (7; 164)
γ	γ (1; 5132)	β (2; 1338)	δ (3; 1229)	α (7; 522)	
δ-t	δ (1; 5901)	α (3; 595)	d (4; 583)	β (5; 351)	OSCP (6; 219)
ε-t	ε (7; 550)	α (1; 1047)	OSCP (9; 417)		

[1] The major proteins (excluding keratins and trypsin) identified in each protein band (Appendix Fig. S5) are listed.

[2] The values in brackets denote the rank of each protein in the identified protein list, and its MASCOT Mudpit score. Tandem MS data from a Q-Exactive instrument were compared with MASCOT to a UniProt protein sequence database (all entries, version August 2020).

Appendix Table S3. Identification of proteins associated with a tagged ATP synthase δ-subunit by tandem MS analysis of tryptic peptides

**Protein**[1] (UniProt)	**Tandem MS data**[2]					
	m/z	Mass (M)		Δmass (ppm)	Sequence	Score[3]
		Observed	Calculated			
Propionyl Co-A carboxylase (P05165)	492.7374	983.4602	983.4600	0.20	VYAEDPYK	38
	525.2887	1048.5628	1048.5627	0.16	EIGYPVMIK	14
	547.3270	1092.6395	1092.6404	-0.79	GVTHNIALLR	27
	553.2861	1105.5620	1105.5615	0.42	VTEDTSSVLR	78
	558.7958	1115.5771	1115.5757	1.28	AMGEQAVALAR	45
	567.7566	1133.4986	1133.4989	-0.24	IAWDDEETR	44
	648.8159	1295.6173	1295.6180	-0.52	MADALDNYVIR	49
	735.3291	1468.6436	1468.6430	0.44	LSSQEAASSFGDDR	57
	781.8631	1561.7116	1561.7116	0.02	MADEAVCVGPAPTSK	32
δ subunit (P30049)	493.7796	985.5446	985.5444	0.28	IEANEALVK	63
	716.3574	1430.7002	1430.7001	-0.22	AQAELVGTADEATR	71
ε subunit (Q5VTU8)	454.2539	906.4932	906.4923	1.01	QAGLSYIR	23

[1] δ-t and ε are from the purification of the δε complex in HEK293-Δ(c+OSCP) cells (Appendix Fig. S7E).

[2] Tandem MS data from multiply charged peptide ions in a Q-Exactive instrument were compared with MASCOT against a UniProt protein sequence database (v. August 2020) containing 20,453 human protein sequences (human proteins, trypsin constraints, variable modifications were Met oxidation and Cys propionamide, maximum of two missed cleavages, peptide mass tolerance ± 5 ppm, fragment mass tolerance ± 0.01 Da).

[3] Individual MASCOT ions scores >17 indicate identity or extensive homology ($p<0.05$). Underlined methionine or cysteine residues are modified by oxidation or propionamide respectively.

Appendix Table S4. Molecular masses of δ- and ε-subunits in δε-complexes determined by LC-MS

Subunit[1]	Mass (Da) Calculated[2]	Observed	Difference	Modification[3]
δ-t	19807.8	19839.0	+31.2	+ 2[O]
		20022.4	+214.6	+2[O] and AEBSF
ε	5648.5	5648.2	-0.3	none
δ-r	15077.0	15077.0	0.0	none
ε-r	5779.8	5779.5	-0.3	none
		5648.5	-131.3	-Met1
		5549.1	-230.7	-Met1Val2

[1] δ-t and ε are from the purification of the δε complex in HEK293-Δ(c+OSCP) cells (Appendix Fig. S7E). δ-r and ε-r are components of the purified recombinant δε complex (Appendix Fig. S8F).
[2] The calculated masses for δ-t and ε correspond to the mature mitochondrial protein after removing the N-terminal mitochondrial targeting peptide (δ-t) or Met1 (ε). For the recombinant proteins, the δ-r mass is for the mature protein with an additional N-terminal Gly, and ε-r is for the full-length sequence (Appendix Figs. S8A and S8B).
[3] Oxidation, probably of methionine residues, occurred during ionization. AEBSF [4-(2-aminoethyl)-benzenesulfonyl fluoride hydrochloride] component of the protease inhibitor mixture in the purification buffer can react with specific serine residues. The sites of modification were not investigated.

Appendix Table S5. Interface areas in predicted assembly complexes involving the α- and β-subunits of h-ATP synthase. See Fig. EV8.

Interacting components	Interface area (Å^2)[a]
β-ATPAF1	4253.8
α-ATPAF2	2092.2
ATPAF2-FMC1	1506.6
FMC1-NDUFAB1	836.5
α-FMC1	741.4
α-ATP	356.5
α-Mg	41.1
ATPAF2-ATP	53.6
ATPAF2-Mg	2.6

[a], calculated with PDBePISA v1.52 (Protein interfaces, surfaces and assemblies' service PISA at the European Bioinformatics Institute; https://www.ebi.ac.uk/pdbe/prot_int/pistart.html).

Appendix Table S6. Target sites for gRNAs employed in the disruption of human genes encoding subunits of ATP synthase and assembly factors for the F_1-module

Protein	gRNA	Sequence
α-subunit	ATP5F1A-1	AACGCGAGGGGCAGTACTTC
	ATP5F1A-2	GCTGTCCGTGCGCGTTGCTG
β-subunit	ATP5F1B-1	GTAGGCCTCGCCTCAACGGC
	ATP5F1B-2	ACGGCGGTCCATCCTGGTAA[1]
γ-subunit	ATP5F1C-1	GCTTCGGCGCATGCGCGCTG
	ATP5F1C-2	TGCCTGACCGACCTTCAGCA
δ-subunit	ATP5F1D	GCTGGTCGTGGTGCATGCAG
ε-subunit	ATP5F1E-1	GCGTCTTGGCGATTCGGCCC[1]
	ATPAF1E-2	CGGGCGTCGCCGCCAAGATG[1]
assembly factor ATPAF1	ATPAF1-1	TATCCCGCGCCCGGAACCTT
	ATPAF1-2	GCTGGATCTTGTCGCGGTAG[1]
assembly factor ATPAF2	ATPAF2-1	AAGATGCGAGACGCGAAACC[1]
	ATPAF2-2	ACGCCGTCTCCTGAATCGGC
assembly factor FMC1	FMC1-1	TCAGGGTCGTAGGACGCCGT
	FMC1-2	CCGACCCTATCGCGACACCG
OSCP-subunit	ATP5PO-1	GAGAACCTAGCGGTTACGCC*
	ATP5PO-2	CCAGGGTGCGATGCTTCGGC
precursor of isoform c1 of c-subunit	ATP5MC1-1	ACAGCAACTTCCCACTCCAG
	ATP5MC1-2	TGTTTGGCAGCTTGATCATT
precursor of isoform c2 of c-subunit	ATP5MC2-1	GCGCCATTTCAAGGGACATC
	ATP5MC2-2	CTGGGATTGGAACTGTGTTT
precursor of isoform c3 of c-subunit	ATP5MC3-1	AAGGGAGTTTCAGACCAGTG
	ATP5MC3-2	GCTGCCAAATTTATTGGTGC

[1]The antisense sequence was employed

Appendix Table S7. Primers employed in the amplification by PCR of regions targeted by gRNAs in human genes encoding subunits of ATP synthase and assembly factors for the F_1-module

Protein	Primer	Sequence
α-subunit	ATP5F1A-FW	GCAACCACAAGGTCGACTCA
	ATP5F1A-REV	GGGACGCGACCCTAAGAATC
β-subunit	ATP5F1B-FW	ACGCCCTCTAACCTCACACT
	ATP5F1B-REV	ATTCAACCGGAAGGTCAACG
γ-subunit	ATP5F1C-FW	TCACTTTCTGGACCCCAGTACTCA
	ATP5F1C-REV	CGCACACCTTGCCCGAC
δ-subunit	ATP5F1D-FW	AGTGCCTCACATCAGCGCCAG
	ATP5F1D-REV	AGCAGGGTCCCCTCTGGTTCGC
ε-subunit	ATP5F1E-FW	CGGGGTAATACGGAAGGTCG
	ATP5F1E-REV	TGTGTCCTGCATGACCTCTG
assembly factor ATPAF1	ATPAF1-FW	ATACACAGGGCGGCGAGAG
	ATPAF1-REV	ACAAACGTGGAACGAGCCAG
assembly factor ATPAF2	ATPAF2-FW	GACTTGTAGTCTCCTCGCCG
	ATPAF2-REW	ACTGTATCGTGCGTGACTCG
assembly factor FMC1	FMC1-FW	AGTCTGTCCGACCGTACCAT
	FMC1-REV	TACCCGATGTGCACGGAAAG
OSCP-subunit	ATP5PO-FW	TACAACTCCCAGCCCGAGG
	ATP5PO-REV	ACGCCAAGGTTACGGCA
precursor of isoform c1 of c-subunit	ATP5MC1-FW	GCAGTTTGCCAACAGTTTCAGAG
	ATP5MC1-REV	GATGAAATCAACCTGAAGGCTCCTG
precursor of isoform c2 of c-subunit	ATP5MC2-FW	AGGCAAATGCTTCTGAGGCT
	ATP5MC2-REV	GTGCCAGTTTTCCCAGGAGT
precursor of isoform c3 of c-subunit	ATP5MC3-FW	ATTTGTCTCTTTACACTCAGCTACA
	ATP5MC3-REV	GCTAAGTTTCCAACTACTGCAAGC

Appendix Table S8. Oligonucleotide primers employed in the amplification by PCR of DNA sequences encoding the γ-, δ- and ε-subunits of human ATP synthase, and assembly factors ATPAF1, ATPAF2 and FMC1.

Protein	Primer	Sequence
γ	ATP5F1C-FW	CGCAAGCTTGGAATGTTCTCTCGCGCGGGTG
	ATP5F1C-REV	CGGCTCGAGATCCAGAGCTGCAGCACCAG
δ	ATP5F1D-FW	CGCAAGCTTGGAATGCTGCCCGCCGCGCTG
	ATP5F1D-REV	CGGCTCGAGCTCCAGGGCCTTCACCAG
ε	ATP5F1E-FW	CGCAAGCTTGGAATGGTGGCCTACTGGAGACAG
	ATP5F1E-REV	CGGCTCGAGTTCCTTCTTTACTTTCACAATTTTTAC
ATPAF1	ATPAF1-FW	CGCAAGCTTGGAATGGCTGCTGTGGTGGTGG
	ATPAF1-REV	CGGCTCGAGAGTCTTATTTTGGTTCTGGGCAC
ATPAF2	ATPAF2-FW	CGCAAGCTTGGAATGTGGAGGAGCTGCCTCC
	ATPAF2-REV	CGGCTCGAGCTCCTTCAGGAGCTTGTGCTTG
FMC1	FMC1-FW	CGCAAGCTTGGAATGGCGGCCTTAGGGTCC
	FMC1-REV	CGGCTCGAGTGGCTCCCAGCCCTTCCCTC
