# Supplementary material for: Assembly of the catalytic module and the rotor of human ATP synthase
Source: EMBO J. 2026 Jun 26;45(15):5482–513. doi: 10.1038/s44318-026-00842-9 (PMC13434801; doi:10.1038/s44318-026-00842-9)
Supplement: Supplementary file 3 [file 44318_2026_842_MOESM2_ESM.pdf]

THE EMBO JOURNAL

Assembly of the catalytic module and the rotor of human ATP synthase

Jiuya He, Joe Carroll, Shujing Ding, Jiao Li, Ian Fearnley, and John Walker

Corresponding author(s): John Walker (jew30@cam.ac.uk)

Review Timeline:

Submission Date:	11th Dec 25
Editorial Decision:	13th Feb 26
Revision Received:	22nd Apr 26
Editorial Decision:	18th May 26
Revision Received:	10th Jun 26
Accepted:	12th Jun 26

Editor: Hartmut Vodermaier

Transaction Report:

(Note: With the exception of the correction of typographical or spelling errors that could be a source of ambiguity, letters and reports are not edited. Depending on transfer agreements, referee reports obtained elsewhere may or may not be included in this compilation. Referee reports are anonymous unless the Referee chooses to sign their reports.)

Prof. John E. Walker
Medical Research Council
Mitochondrial Biology Unit
Wellcome Trust/MRC Building
Hills Road
Cambridge CB2 0XY
United Kingdom

13th Feb 2026

Re: EMBOJ-2025-123282
Assembly of the catalytic domain and the rotor of human ATP synthase

Dear Prof. Walker,

Thank you again for submitting your study on assembly of human ATPase constituents, as well as for your patience during its evaluation. I am very sorry for the unusual delays, caused by limited referee availability at this time of the year. We have now received the reports of three expert referees, and since they came back with somewhat divided opinions, we also discussed the work with an Editorial Board Advisor of our journal.

As you will see from the comments below, all referees appreciate the extensive and rigorous experimental work, as well as the potential importance of its results. However, they at the same time bring up two types of major concerns that preclude publication of the work in its present form:
- From the experimental side, referees 1 and 3 both raise a number of specific queries, which would need to be adequately responded to prior to publication.
- From the presentational side, all referees as well as our expert advisor felt that the study would need to be fundamentally reorganized in order to make it a good candidate for an EMBO Journal article. In particular, this would require a much clearer statement and outlining of the question(s) (and their significance) that the study aims to address; and a stronger emphasis of the key novel findings and their implications.

Given that a majority of referees and our advisor did nevertheless consider the essence of this study of broad interest, we would be open to considering a revised version further for EMBO Journal publication, pending adequate revisions of the key points mentioned. In particular, in the interest of our broad non-specialist readership, it will be essential to make the paper more easily accessible for general readers. This should include a restructured abstract, opening with only 1-2 sentences of background and key open question, followed by description of experimental strategy and key results, and closing with 1-2 sentences mentioning conclusions/implications/significance of the study and its findings. Similarly, the introduction may benefit from describing less of the general background and instead more concretely outlining remaining open questions and their significance. Finally, I would strongly suggest attempting to reorganize figures and data presentation, taking advantage of the figure hierarchy offered by EMBO Press: Main Figures (8-10) - Expanded View Figures (up to ~10; still typeset and directly available in online HTML views) - Appendix Figures (collected in a single Appendix PDF that should also incorporate the Appendix Table S1-S8). Regarding the large number of Expanded View Datasets predominantly containing proteomics datasets, it would probably be preferable if they could be collectively deposited to a public repository, or else at least combined in fewer files. If needed, we would be happy to provide further guidance on these issues.

Please be reminded that it is our policy to consider only a single round of major revision, making it important to adequately answer all comments at the time of resubmission; therefore, please do not hesitate to get back to me in case you would like to clarify/discuss any of the referees' points, or options for reorganization of the manuscript.

Detailed further information on preparing, formatting and uploading a revised EMBO Journal manuscript can be found below and in our Guide to Authors, and adhering to them as closely as possible shall greatly facilitate editorial processing upon resubmission. Thank you again for the opportunity to consider this work for The EMBO Journal, and I look forward to your revision.

Yours sincerely,

Hartmut Vodermaier

Hartmut Vodermaier, PhD
Senior Editor, The EMBO Journal
h.vodermaier@embojournal.org

*** PLEASE NOTE: All revised manuscript are subject to initial checks for completeness and adherence to our formatting guidelines. Revisions may be returned to the authors and delayed in their editorial re-evaluation if they fail to comply to the following requirements. As a first step please read our guidelines for revised submissions: https://link.springer.com/journal/44318/submission-guidelines#cms-Revised-submissions

1) Every manuscript requires a Data Availability section (even if only stating that no deposited datasets are included). Primary datasets or computer code produced in the current study have to be deposited in appropriate public repositories prior to resubmission, and reviewer access details provided in case that public access is not yet allowed.

2) Each figure legend must specify
- size of the scale bars that are mandatory for all micrograph panels
- the statistical test used to generate error bars and P-values
- the type error bars (e.g., S.E.M., S.D.)
- the number (n) and nature (biological or technical replicate) of independent experiments underlying each data point
- Figures may not include error bars for experiments with n<3; scatter plots showing individual data points should be used instead.

3) Revised manuscript text (including main tables, and figure legends for main and EV figures) has to be submitted as editable text file (e.g., .docx format). We encourage highlighting of changes (e.g., via text color) for the referees' reference.

4) Each main and each Expanded View (EV) figure should be uploaded as individual production-quality files (preferably in .eps, .tif, .jpg formats). For suggestions on figure preparation/layout, please refer to our Figure Preparation Guidelines: https://media.springernature.com/original/springer-cms/rest/v1/content/27825798/data/v1

5) Point-by-point response letters should include the original referee comments in full together with your detailed responses to them (and to specific editor requests if applicable), and also be uploaded as editable (e.g., .docx) text files.

6) Please complete our Author Checklist, and make sure that information entered into the checklist is also reflected in the manuscript; the checklist will be available to readers as part of the Review Process File.

7) All authors listed as (co-)corresponding need to deposit, in their respective author profiles in our submission system, a unique ORCiD identifier linked to their name. Please see our Guide to Authors for detailed instructions.

8) Please note that supplementary information at EMBO Press has been superseded by the 'Expanded View' for inclusion of additional figures, tables, movies or datasets; with up to five EV Figures being typeset and directly accessible in the HTML version of the article.

9) To facilitate reproducibilty and cross-laboratory adoption of methodologies, please structure the Materials & Methods section as outlined in our guide to authors, including a completed Reagents and Tools Table.

10) Digital image enhancement is acceptable practice, as long as it accurately represents the original data and conforms to community standards. If a figure has been subjected to significant electronic manipulation, this must be clearly noted in the figure legend and/or the 'Materials and Methods' section. The editors reserve the right to request original versions of figures and the original images that were used to assemble the figure. Finally, we generally encourage uploading of numerical as well as gel/blot image source data.

At EMBO Press we ask authors to provide source data for the main manuscript figures. You will receive a separate email with instructions for providing source data with your revised manuscript, including how to upload and organize the files.

In the interest of ensuring the conceptual advance provided by the work, we recommend submitting a revision within 3 months (14th May 2026). Please discuss the revision progress ahead of this time with the editor if you require more time to complete the revisions. Use the link below to submit your revision:

Link Not Available

Referee #1:

In their manuscript, He and colleagues present a large body of data in order to investigate which order the subunits of the

human mitochondrial ATP synthase assemble to form the complete monomeric complex. In particular, they looked into the question of the assembly of the F1-c8-ring subcomplex, the F1 domain (α3β3γδε), the central stalk (γδε), the rotor (γδεc8-ring) and the c8-ring. This study relies mainly on experimental evidence from immuno staining of denaturing and native gel electrophoresis gels of solubilized mitoplasts or tag-purified subunits, in tandem with mass spectrometry, to identify assembly intermediates. To a lesser extent the study also draws on insights obtained from respiratory activity measurements and structure prediction using AlphaFold3. I laud their considerable efforts into the of the assembly pathway of the F1-c8-ring subcomplex of human ATP synthase. This work connects well to the authors' previous reports on the assembly of mitochondrial ATP synthase.

Before publication, however, it is advisable to improve the manuscript in a range of aspects. A key problem with the manuscript in its current form is the presentation of the results in both text and figures. It is too hard for the general reader to understand what open questions motivated this study, which means were used to answer these questions, how definite the conclusions made are and where the limitations of the methods employed in this study are. Given the large amount of experimental evidence reported here, this shortcoming should be addressed.

For instance, in its current form, the abstract is, except for the last sentence, an introduction to human ATP synthase function and structural organization. However, neither the open questions addressed in this study, nor the methods of investigation are mentioned. In addition, the main conclusion of this study, which I believe is presented in Figure 9, is also not included in the abstract.

Throughout the manuscript, the numerous names of human ATP synthase subunits, their corresponding gene names, together with terms for structural modules, domains, subcomplexes, rotor and stator are used and quite often arbitrarily mixed. For example, the structural module central stalk (CS) is introduced with Fig. 1B, however, the panel for Fig. 1B shows the CS attached to the c8-ring, which constitutes the rotor and not just the CS. These lapses in consistency and clarity could easily be avoided by adding a table listing all the subunit names, gene names, structural modules, domains, and their respective functionality. Together with an improved Fig. 1 this should be very helpful to the reader who wants to follow the line of thought presented in the main text.

Regarding Figure 1A: why is IF1 not shown though PDB 6ZQM includes IF1?

An earlier study on the human ATP synthase assembly pathway using a similar experimental strategy (Fujikawa et al., FEBS Letters, 2017) is not referred to at all. Why is that? Do the authors' findings disagree with this earlier study? Some comment is warrented.

On page 6, Line 1 the presence of uncharacterized precursors of the F1 domain is postulated. I can not follow the logic of this conclusion nor which experiment on which the evidence rests. Please clarify.

Also, on page 6 a reduction of 74% in the level of subunit k is termed to be not substantial. This raises the question of how much reduction is substantial?

On page 7, the relative enrichment of subunit c at a level lower than that of γδε subunits is taken as evidence for the presence of an assembled CS complex plus the presence of fully assembled c8-rings. Does "plus" here mean assembled as a rotor or does it mean separately assembled? And how is the relative abundance of subunits strong evidence for the presence of specific assembly intermediates?

Even though the section on assembly factors on page 9 is still part of the results section overall conclusions are offered before experimental results are described. The presentation of experimental insights and conclusion drawn for the roles of ATPAF1, ATPAF1 and FMC1 in this section of the manuscript should be put in proper order and clarified.

The most valuable insights of this study are summarized in Figure 9 on the apparent co-existence of two assembly pathways for the formation of the F1-c8-ring subcomplex. This summary figure is followed up by two figures on the insight gained from AlphaFold3 predictions, for which there is no hard experimental verification available. Perhaps, moving them to the supplementary appendix makes more sense. In addition, with the Walker lab being a pioneer in the structural analysis of ATP synthase by experimental methods, i.e., X-ray crystallography and cryo-EM, a few critical words on the potential pitfalls of using such computational methods for structural analysis are warranted. For example, even though the ground state structure of the α3β3γ assembly as determined by X-ray crystallography by the Walker lab several decades ago clearly showed an asymmetric arrangement, AlphaFold3 predictions of the α3β3γ assembly turn out with all β-subunits in the same conformation. A clear indication of important limitations in using AlphaFold3. In the current manuscript I dearly miss that kind of critical discussion.

Related, the absence of any cryo-EM data on the by affinity tag purified assembly intermediates needs to be discussed. Since the Walker lab has pioneered cryo-EM of mitochondrial ATP synthase and produced outstanding results, at least mentioning why the structural analysis of affinity tag purified complexes was not done (yet?) is of interest. A very recent study on the assembly of human cytochrome c oxidase yielded very interesting results using similar experimental approaches by incorporating single particle cryo-EM in their study:

See Nguyen et al., Nat. Comm., 2026
https://www.nature.com/articles/s41467-025-68274-3

I don't think cryo-EM needs to be incorporated in the present study, however, informing the reader why it was not done is of interest. Is perhaps the yield of the tag purified complexes too low?

Page 14, Line 2: What is the connection between Figure 4 and ATPAF1? At least there is no explicit mentioning of ATPAF1 in Figure 4. Am I missing something? Please check and/or clarify.

Please provide line numbering next time.

Referee #2:

He and coworkers study assembly intermediates and pathways for human mitochondrial FoF1 ATP synthase. In a rigorous tour-de-force, the authors generate HEK293 single and multiple subunit knock-out lines for nuclear encoded F1 subunits alpha, beta, gamma, delta, epsilon, Fo subunit c variants and OSCP, as well as F1 assembly factors AF1, AF2, and FMC1. For each KO line, he authors then employ native PAGE and/or SILAC mass spectrometry as well as mass spectrometry of pull-downs using tagged gamma subunit to investigate presence or absence of sub-complexes and quantify expression levels. In addition, they measure oligomycin sensitive oxygen consumption and expression levels of respiratory complex subunits as a function of F-ATPase subunit knockouts. The main conclusions from the study are that a variety of F-ATPase subcomplexes can assemble independently, including a complete F1, the central rotor (gamma:delta:epsilon), with or without the c8 ring of the Fo, and a delta:epsilon heterodimer. The authors also used Alphafold3 to predict structural models and complexes of assembly factors with F1 subunits (AF1 with beta; AF2:FMC1:NDUFAB1:alpha).

Mitochondrial F-ATP synthase is an essential enzyme complex, and learning more about its structure and mechanism of assembly is certainly important. Overall, while the work appears technically sound and of high quality, considerable knowledge about assembly factors and subcomplexes is already available (as acknowledged by t he authors at several places in the manuscript), and it therefore not entirely clear what fundamental new insight is obtained by the large amount of data. Moreover, the work appears somewhat descriptive as the authors don't really highlight what the motivation for the work was, and what the significance of the findings is. As it stands, the manuscript may be more suitable for a more specialized journal.

Referee #3:

In their manuscript, He et al analyze subcomplexes of the human mitochondrial ATP synthase upon deletion of subunits or assembly factors and draw conclusions about possible assembly pathways. While a previous study of theirs focused on assembly of the membrane-integral components, now they complement those insights with a study on assembly of the F1 part of the ATP synthase. They report that the F1 part can assemble independently, or alternatively, the rotor and the central stalk can assemble. They discover a complex consisting of the central stalk subunits delta and epsilon although its significance for the assembly process remains unclear. The authors also investigate the contribution of the F1 assembly factors ATPAF1, ATPAF2 and FMC1. ATPAF1 forms a complex with the beta subunit, as expected, but is not strictly required for beta assembly. The contribution of the most important factor ATPAF2 remains unclear, but the authors propose a quaternary complex of ATPAF2, alpha subunit, FMC1 and NDUFAB1.
The study reports important insights into the assembly pathway of the mitochondrial ATP synthase using a large number of experiments and broad range of methods. However, the presentation and validity of conclusions are sometimes opaque due to an overwhelming amount of figures that are not easily accessible. In some cases, necessary information is not provided and it remains unclear whether the conclusions can be supported. Overall, the manuscript would benefit from comprehensive streamlining with a view to clarity, both in the presentation of figures and in the logic / major conclusions.

Major points:
1. On p. 7 the authors conclude that tagged gamma-subunit does not interact with alpha or beta in the absence of delta or epsilon subunits, or with delta in the epsilon deletion and vice versa. However, as the authors show in Figs. 3, 5, S7 and S8, there is no gamma subunit present in the absence of delta or epsilon, nor is there delta subunit in the epsilon deletion and vice versa. Also the alpha subunit is strongly reduced in these strains. If expression of the tagged gamma subunit changes these behaviours, the authors have not shown it. Therefore these conclusions are not meaningful.
2. The delta-epsilon complex in gamma deletion mitochondria (Figs. 5, 6) contradicts the negligible expression levels of the delta and epsilon subunits in that background (Figs. 3, S7). How do the authors explain this discrepancy?
3. In several instances the authors analyzed "immunopurified" ATP synthase (sub)complexes without stating even once against which protein(s) the antibodies for purification were directed. Without this information, the significance of the results cannot be assessed. For example, what species of ATP synthase were purified from mitochondria lacking ATPAF2 where almost no mature ATP synthase is produced, and how are the results comparable to isolations from ΔATPAF1 or ΔFMC1 mitochondria where some ATP synthase can assemble (Fig. 8, Fig. S18)?

4. In Fig. S14, the a-delta and a-epsilon blots display a non-stoichiometric / non-correlative behaviour of the two subunits in the 200 kDa complex in different assembly factor deletion strains, in contrast to the proposed equimolar delta-epsilon complex. Additionally, the a-epsilon blot shows a complex in the last lane migrating like F1 that was not detected with the alpha or beta antbodies even though their sensitivity appears similar. How do the authors explain these findings?
5. Regarding oxygen consumption of deletion strains (Fig. S30), the authors state on p. 11 that there is a small amount of oligomycin-sensitive oxygen consumption in strains lacking gamma, delta or epsilon subunits. However, such an effect is only visible in the data for the gamma and epsilon single deletions. The authors further conclude from these results that there is "a minor uncoupled partially assembled ATP synthase complex containing the c-subunit, but lacking an intact transmembrane proton pathway". If there is some coupling between ATP synthase activity and oxygen consumption, doesn't this rather indicate that the activity of the subcomplex is coupled to the respiratory chain and that it does have a transmembrane proton pathway? The authors should immunopurify ATP synthase in these strains to test which subunits are present.

Minor points:
1. The handling of figures is rather inflationary and there are very few panels per figure, with some relevant results buried in the supplement. For example, Fig. S4 is very informative (the first sentence of the discussion references the results from this figure) and should become a panel of Fig. 2; Fig. S7 should be included in Fig. 3.
2. On p. 6 the authors argue based on Fig. 3 that because the subunits of the rotor maintain intermediate levels in deletions of the alpha or beta subunits, this indicates that a central stalk complex and possibly the rotor can be assembled. This conclusion only becomes plausible once the complex isolations in Fig. 4 are shown and discussed. The authors should combine the two figures and draw their conclusion when it's appropriate.
3. The antibody treatments of blots should be labeled in the figure so readers don't have to search for this crucial information in the legend. E.g. in Fig. 5, rather than labeling each panel A-F they should be labeled by the antibodies used. Similarly, for SILAC-MS analysis of different strains (e.g. in Fig. S8), the strains should be named in the figure panels.
4. The authors state about Fig. S29 that reexpression of ATPAF2 led to a recovery of alpha, beta, gamma and epsilon subunits. However, the data show a recovery of subunits excluding alpha already with 0 mg/ml dox, and actual ATPAF2 induction leads to a reduction of gamma and epsilon. Only the ATPAF1 reexpression is doxycyclin-dependent. The authors need to discuss the issues here (leaky expression etc).
5. The tag(s) used should be spelled out at least once in the main text or the figure legend, instead of just labeling the proteins -tag or -t.
6. Fig. S15 does not display any grey areas (to indicate a-helices). In Fig. S32 it is not possible to distinguish the two proteins.
7. Can the authors reference any experimental evidence for the complexes proposed in Fig. 10B+C? Since the study does not offer insight into the precise functions of ATPAF2 and FMC1, the authors should also reference relevant literature on their roles to support the statement that they, together with ATPAF1, fashion the alpha3beta3-sphere.

Responses to Referees of Manuscript EMBOJ-2025-123282

We thank the three Referees for their constructive and helpful comments on our manuscript, and the Editor for his guidance.

The Editor wrote: *"all referees appreciate the extensive and rigorous experimental work, as well as the potential importance of its results."* However, *"all referees as well as our expert advisor felt that the study would need to be fundamentally reorganized in order to make it a good candidate for an EMBO Journal article. In particular, this would require a much clearer statement and outlining of the question(s) (and their significance) that the study aims to address; and a stronger emphasis of the key novel findings and their implications."*

In response to the Editor's guidance and the Referees' comments, in the revised manuscript the following changes have been made:

1. General Information

1.1. In response to a request from Referee 1, the lines in the revised manuscript are numbered. We have used this numbering in our responses below.

1.2. In response to Referee 1, for clarity of description of the various parts of the ATP synthase and their functions, in the new Table EV1 we have defined the terms "module", "domain", F_1, F_o and "peripheral stalk" and provided their compositions and functions. These terms are employed strictly throughout the revised manuscript. This has necessitated the replacement of "domain" by "module" in the main title of the paper.

1.3. For brevity and clarity, we have introduced and defined abbreviations such as "h-ATP synthase" for "human ATP synthase", "PS" for "peripheral stalk, "AF" for "assembly factor" and so forth, and once introduced, used them throughout the revised manuscript.

1.4. The revised manuscript has been re-organised into the format required by the EMBO Journal.

1.4.1. The revised manuscript now has an EV section containing 10 Figures and one new Table.

1.4.2. A supporting Appendix section has been constructed as a separate document. It contains 22 Figures (reduced from 33 in the original submission) and the 8 original Tables.

2. Detailed Information.

The manuscript has been extensively revised and reorganised as follows.

2.1. The Abstract has been completely re-written. Within the confines of the 175 word maximum, the revised Abstract, is now structured with an introduction to the topic (lines 16-20), followed by what our objective are (lines 20-21), what we have done experimentally including the main methodology (lines 21-23), followed by our main conclusions (lines 23-24), what further opportunities arise (lines 24-25), and what other conclusions can be drawn (in relation to the evolution of ATP synthase) (lines 25-28).

These changes respond to Referee 1 who said that: "*The abstract is, except for the last sentence, an introduction to human ATP synthase function and structural organization. However, neither the open questions addressed in this study, nor the methods of investigation are mentioned. In addition, the main conclusion of this study, which I believe is presented in Figure 9, is also not included in the abstract*".

2.2. The Introduction has also been re-written and re-organised. It now roughly follows the same structure as the Summary, but with greater detail. Thus, first we provide the requisite structural background (lines 33-79). Second, we give the background of previous studies on the topic of the assembly of the h-ATP synthase (lines 80-101). Third, we state what the problem is, what we have achieved in this paper, and the relevance of our findings to both how the h-ATP synthase is assembled and how it probably has evolved (lines 102-106). Finally, we mention the experimental approaches used (lines 106-109).

2.3. Results

The Results have been edited extensively to remove any unnecessary text, and the Figures have been changed in response to the suggestions of Referees.

2.3.1. The first section (lines 111-121) entitled 'Independent assembly of the h-F_1-module' now includes a revised Fig. 2 as suggested by Referee 3 (minor points 1, see below). Specific responses to Referees 1 and 3 are also given (see responses to Referees below).

2.3.2. Sections 2 and 3 of the original document, "*Formation of the central stalk in the absence of α- and β-subunits*" and "*The rotor complex*", have been combined into one section now entitled "The CS and the rotor complex assemble in the absence of α- and β-subunits" (lines 122-156). Changes to Figs. 3 and 4 in response to Referee 3 minor points 1 and 2 (see responses to Referees below) have been incorporated.

2.3.3. The section entitled "The δε complex" (lines 157-173) now includes a new Fig. 4, again in response to Referee 3 (see Referees' responses below).

2.3.4. The section "Incorporation of the α- and β-subunits" (lines 174-205) now contains a more complete description of the results presented in Fig. 5 (originally Fig. 7).

2.3.5. The section "Roles of assembly factors ATPAF1, ATPAF2 and FMC1" (lines 206-240) has been re-organized in response to Referee 1, point 9 (see responses to Referees below).

2.3.6. The final section of Results in the original document entitled "*Disruption of assembly of the F_1-domain of ATP synthase and respiration*", and associated Figures, have been deleted in response to Referee 3, major point 5. These experiments illuminate the effects of the deletion of subunits on cellular physiology, and as such they are not directly in the main theme of the paper, namely, the elucidation of the pathway(s) of assembly of the h-ATP synthase. We intend to publish them in an independent article.

2.4. Discussion

This section has also been re-written extensively.

As before, it has eight paragraphs, now arranged in a logical order with the following contents:

2.4.1. Para 1 (lines 242-246) summarizes the first finding in the Results section, namely that the F_1-module can be assembled independently of all the other subunits.

2.4.2. Para 2 (lines 247-261) summarizes the second such finding, namely that the central stalk (CS) and of c_8-ring also be assembled independently, and that subsequently they join together to form the rotor.

2.4.3. Para 3 (lines 262-271) outlines three potential assembly pathways for forming the F_1-module and concludes with two possible routes for the most probable of the three pathways.

2.4.4. Para 4 (lines 272-286) discusses the roles of the three AFs (ATPAF1, ATPAF2 and FMC1) in forming the F_1-module. It emphasises that the structurally related α- and β-subunits are not introduced in the same way. The introduction of β-subunits involves the formation of a stable dimeric intermediate with ATPAF1, and the elongated α-helical part of the γ-subunit may displace ATPAF1 during the assembly process of β-subunits. The introduction of α-subunits appears to proceed with the aid of a trimeric complex made of AFs ATPAF2 and FMC1, and additionally NDUFAB1.

The structures of these proposed intermediate complexes modelled with AlphaFold 3 are presented in Figs. EV8 and EV9 and Appendix Fig. S20 and 21, and are discussed. Comments about AlphaFold are in Para 7 below.

Referee 1 also states that "*I don't think cryo-EM needs to be incorporated in the present study, however, informing the reader why it was not done is of interest*." We agree that cryo-EM analysis of the intermediates that we have characterized is not warranted here, given the already extensive number of experiments and their conclusions that are described. However, it is a distinct future possibility that we are investigating via a collaboration, as the activities of this research group ceased on April 1st, 2026. The amounts of at least some of the intermediates that can be purified should be adequate to support such investigations.

2.4.5 Para 5 (lines 287-305) continues the discussion of the roles of AFs.

2.4.6 Para 6 (lines 306-328) draws attention to two important general conclusions that can be made about the assembly of human ATP synthase. First, AFs are required for constructing oligomeric rings, and not for introducing single copy subunits. Second, the assembly process avoids generating intermediates that could either hydrolyze ATP or uncouple mitochondria.

2.6.7 Para 7 (lines 329-353). Here, we summarize a number of unsolved issues relating to the assembly of the h-ATP synthase, and we discuss the limitations of AlphaFold 3, in response to Referee 1 who wrote: "*few critical words on the potential pitfalls of using such computational methods for structural analysis are warranted. For example, even though the ground state structure of the α3β3γ assembly as determined by X-ray crystallography by the Walker lab several decades ago clearly showed an asymmetric arrangement, AlphaFold3 predictions of the α3β3γ assembly turn out with all β-subunits in the same conformation. A clear indication of important limitations in using AlphaFold3*".

In the revised manuscript, we have mentioned improvements of AlphaFold3 over earlier versions, especially its ability to predict structures of protein complexes. The referee's statement that "*α3β3γ assembly turn out with all β-subunits in the same conformation*" is incorrect: an asymmetric structure of $\alpha_3\beta_3\gamma$ with five bound nucleotides (ie with one potential nucleotide binding site empty) is predicted (although we do not present it here, as it is not required). This asymmetric prediction is a direct consequence of these AlphaFold3 predictions being based on known (and asymmetric) structures of F_1-ATPase and ATP synthase deposited in structural data-bases.

2.6.8 Para 8 (lines 354-363). In this final paragraph of the Discussion, we outline how some of the observed assembly intermediates are consistent with the notion that the ATP synthase arose by modular evolution. As this is a subsidiary and secondary topic, the discussion here is brief. However, we have now introduced a new Fig. EV10 to help illustrate the concept.

2.5. Figures

Fig. 1: Now shows the "modules" and "domains" to link with Table EV1. The original cartoons of the previously characterized assembly intermediates originally in Fig. 1 are now Fig. EV1.

Fig. 2: now includes original Fig. S4, as requested by Referee 3, minor point 1.

Fig. 3: now is a combination of original Figs. 3 and 4 and S7, in response to Referee 3, minor points 1 and 2.

Fig. 4: is now a combination of original Figs. 5 and 6, as part of the response to the comment by Referee 3 (minor point 1) that '*The handling of figures is rather inflationary and there are very few panels per figure*'.

Figs. 5-7 are the same as the original Figs. 7-9.

As suggested by Referee 1, the main figures now end with Fig. 7, which summarizes the findings in the current paper. The original Alphafold Figs. 10 and 11 are now Figs. EV8 and EV9.

3. Detailed responses to Referees

3.1. Referee 1

Point 1. A key problem with the manuscript in its current form is the presentation of the results in both text and figures. It is too hard for the general reader to understand what open questions motivated this study, which means were used to answer these questions, how definite the conclusions made are and where the limitations of the methods employed in this study are. Given the large amount of experimental evidence reported here, this shortcoming should be addressed.

As described above, we have responded to this helpful advice, and made appropriate and extensive changes.

Point 2. Throughout the manuscript, the numerous names of human ATP synthase subunits, their corresponding gene names, together with terms for structural modules, domains,

subcomplexes, rotor and stator are used and quite often arbitrarily mixed. …… These lapses in consistency and clarity could easily be avoided by adding a table listing all the subunit names, gene names, structural modules, domains, and their respective functionality. Together with an improved Fig. 1 this should be very helpful to the reader who wants to follow the line of thought presented in the main text.

As described above, we have systematised references to modules, domains and so forth in the text and provided a new Table EV1 and a new Figure 1.

Point 3. Regarding Figure 1A: why is IF1 not shown though PDB 6ZQM includes IF1?

IF_1 is not a constituent subunit of the active ATP synthase complex, nor is it part of the structural organisation of the modules and domains that we are discussing in the paper. Thus, it is not required, and for those reasons it has been omitted from the Figure. We now say in the legend of the new Fig. 1 that "IF_1 present in PDB 6ZQM has been removed."

Point 4. An earlier study on the human ATP synthase assembly pathway using a similar experimental strategy (Fujikawa et al., FEBS Letters, 2017) is not referred to at all. Why is that? Do the authors findings disagree with this earlier study? Some comment is warrented.

We thank the referee for pointing out this omission. In the revised manuscript, we have referred to the publication by Fujikawa et al (2015), where the F_1-c_8 complex was identified following deletion of the d-subunit (line 87). However, our findings differ, as in the absence of subunit d we established the presence of a larger complex F_1-c_8-OSCP-F_6-b-e-f-g (He et al., 2020). This difference is referenced (lines 84-87). Fujikawa et al (2015) also identified, as now noted (lines 90-92), an independent assembly of a PS precursor complex of subunits b, e and g (probably also containing subunit f, as shown in He et al., 2020).

Point 5. On page 6, Line 1 the presence of uncharacterized precursors of the F1 domain is postulated. I can not follow the logic of this conclusion nor which experiment on which the evidence rests. Please clarify.

In Figs. 2A and B of the revised manuscript it is shown that in samples captured from mitoplasts of Δ(c+OSCP) cells with an ATP synthase specific immunocapture reagent, in addition to the components of F_1-c_8, assembly factors (AFs) ATPAF1, ATPAF2 and FMC1 were also present and enriched relative to the F_1 subunits. Because these AFs are not part of the catalytic $\alpha_3\beta_3$ domain of ATP synthase, we postulated that their presence indicates the existence of partially assembled catalytic domains containing them. We have clarified the text now to say that: "Also, AF proteins for F_1, namely ATPAF1, ATPAF2 and FMC1 (Wang et al., 2001; Li et al ., 2017) were detected at low levels (Fig. 2A) and were enriched relative to the captured F_1-module components (Fig. 2B), suggesting that the extract might contain partial assemblies of the F_1 catalytic module with bound AFs" (lines 118-121).

Point 6. Also, on page 6 a reduction of 74% in the level of subunit k is termed to be not substantial. This raises the question of how much reduction is substantial?

We thank the referee for pointing this out. In the revised manuscript, we have changed the original sentence to now read: "The levels of most other subunits of the enzyme were reduced to an average of 2-13% of WT levels, except for subunit k, which was reduced to 26% of the WT level" (lines 129-131)

Point 7. On page 7, the relative enrichment of subunit c at a level lower than that of γδε subunits is taken as evidence for the presence of an assembled CS complex plus the presence of fully assembled c8-rings. Does "plus" here mean assembled as a rotor or does it mean separately assembled?

We are referring to the presence of a mixture of CS (γδε subunits) and complete rotor (γδε+c_8-ring). The text now reads: "Here, the relative enrichment of subunit c, although significant, was lower than those of the γ-, δ- and ε-subunits, consistent with the presence of a CS complex and a complete CS-c_8 rotor." (lines 146-148)

Point 8. And how is the relative abundance of subunits strong evidence for the presence of specific assembly intermediates?

The presence of specific assembly intermediates is predicated on the co-purification of subunits, and hence similar enrichment levels, using the ATP synthase immunocapture kit or by capture with an expressed tagged protein. Based on the known structure of the assembled ATP synthase and the defined subunit interactions, combined with similar relative abundances versus a non-binding control (CSKMT-t), we can make an educated judgment on the intermediate assembly complexes found in the captured sample. For examples the histogram in Fig. 2B shows a similar abundance for subunits of the F_1 domain, relative to the control. Native-PAGE can also aid the interpretation.

Point 9. Even though the section on assembly factors on page 9 is still part of the results section overall conclusions are offered before experimental results are described. The presentation of experimental insights and conclusion drawn for the roles of ATPAF1, ATPAF1 and FMC1 in this section of the manuscript should be put in proper order and clarified.

This section has now been re-organised as requested by the referee.

Point 10. The most valuable insights of this study are summarized in Figure 9 on the apparent co-existence of two assembly pathways for the formation of the F1-c8-ring

subcomplex. This summary figure is followed up by two figures on the insight gained from AlphaFold3 predictions, for which there is no hard experimental verification available. Perhaps, moving them to the supplementary appendix makes more sense.

We have moved the two AlphaFold figures into the EV section as suggested by the referee. They are now Figures EV8 and EV9. The former Fig. 9 (now Fig. 7), summarising the assembly pathways is now the final Figure in the manuscript.

Point 11. Re. AlphaFold "***a few critical words on the potential pitfalls of using such computational methods for structural analysis are warranted"*** and ***Related, the absence of any cryo-EM data on the by affinity tag purified assembly intermediates needs to be discussed.***

In the Discussion (lines 334-338) we have introduced comments about the improvements that Alphafold3 brings over previous versions, for example in the prediction of the structures of protein complexes, and we have also discussed (lines 338-342) its limitations e.g. in relation to intrinsically disordered regions of proteins.

Point 12. Page 14, Line 2: What is the connection between Figure 4 and ATPAF1? At least there is no explicit mentioning of ATPAF1 in Figure 4. Am I missing something? Please check and/or clarify.

The referee is correct. We should have referred to Fig. 5, not Fig. 4. In the new version with modified figures this is now Figs. 4A and 5A (lines 275-276).

Point 13. Please provide line numbering next time

Please see Section 1.1 above.

3.2. Referee 2

It is ***"not entirely clear what fundamental new insight is obtained by the large amount of data. Moreover, the work appears somewhat descriptive as the authors don't really highlight what the motivation for the work was, and what the significance of the findings is. As it stands, the manuscript may be more suitable for a more specialized journal."***

The motivation for our work is to understand how 27 proteins emanating from two different genomes (nucleus and mitochondrial DNA) can be assembled (some in multiple copies) in the inner membranes of mitochondria, into the two constituent sub-structures of the rotor and the stator to form the complex structure of the ATP synthase, and without generating intermediate complexes that could either hydrolyze ATP or uncouple mitochondria. These points are now emphasized in the revised manuscript, and our motivation should now be clear. The elucidation of the pathways of the assembly of the rotor and of the catalytic F_1-module of the stator are fundamental advances in understanding how Nature constructs such a complicated machine. They also indicate how it might have evolved. As such, they are foundational contributions to understanding the central problem in biology of how the ATP to sustain life is generated.

3.3. Referee 3

General comment. ***the presentation and validity of conclusions are sometimes opaque due to an overwhelming amount of figures that are not easily accessible. In some cases, necessary information is not provided and it remains unclear whether the conclusions can be supported. Overall, the manuscript would benefit from comprehensive streamlining with a view to clarity, both in the presentation of figures and in the logic / major conclusions.***

We thank the Referee for this helpful comment. As explained above, based on other helpful guidance from the Editor and Referees, the various sections of the manuscript and key Figures and Tables have been extensively revised, stream-lined and reorganised. In doing so, we have clarified the conclusions and limitations of our work.

Major Points:

Point 1. On p. 7 the authors conclude that tagged gamma-subunit does not interact with alpha or beta in the absence of delta or epsilon subunits, or with delta in the epsilon deletion and vice versa. However, as the authors show in Figs. 3, 5, S7 and S8, there is no gamma subunit present in the absence of delta or epsilon, nor is there delta subunit in the epsilon deletion and vice versa. Also the alpha subunit is strongly reduced in these strains. If expression of the tagged gamma subunit changes these behaviours, the authors have not shown it. Therefore these conclusions are not meaningful.

We would like to emphasise that for quantifying the levels of subunits, relative abundance measurements obtained by quantitative MS are far superior to western blotting, which is at best a semi-quantitative method. Figs. 3G and H of the revised manuscript show that in HEK293-Δβ cells we can demonstrate that tagged-γ interacts with δ and ε, where the residual levels of δ and ε are 26-30% (Fig. 3C). Also, there is additional competition for binding to the δ- and ε-subunits from a 22% residual endogenous level of the γ-subunit. Quantitative MS (Fig. 3D) shows that there is a residual 10-22% level of the α and β subunits in HEK293-Δγ cells. Even in the absence of any increase in α and β levels due to an expression effect of tagged-γ, the sensitivity of the MS analysis should be sufficient to pick up an association and enrichment of these subunits with the bait protein, relative to the non-binding control. Indeed, α and β were detected, but not enriched (Fig. EV4). This supports our conclusion that the tagged γ-subunit does not interact with α or β in the absence of δ or ε subunits. However, we agree with the

referee that in the absence of a protective effect of expressing γ-t on the levels of δ in the absence of ε, and *vice versa*, it is unlikely that we would observe any interaction. We have therefore removed the sentence "Nor did γ-t form a binary complex with the δ-subunit expressed in the absence of the ε-subunit, and *vice versa*".

Also, we have deleted from the first paragraph of the section "Incorporation of the α- and β-subunits" the statement that: "as described above, in the absence of the ε-subunit, γ-t did not form a stable complex with the δ-subunit. Likewise in the absence of the δ-subunit, no complex was formed between the ε-subunit and γ-t."

Point 2. The delta-epsilon complex in gamma deletion mitochondria (Figs. 5, 6) contradicts the negligible expression levels of the delta and epsilon subunits in that background (Figs. 3, S7). How do the authors explain this discrepancy?

In the revised manuscript, this comment now refers to Figures 3 and 4. In Fig. 3D, the residual level of subunits δ and ε in Δγ cells was 10% (Fig. 3D), which is 2-4 fold higher than the measured levels of either subunit in the absence of the other (Figs. 3E and F). This suggests some mutual protective effect, making it possible to detect the δε complex observed in Fig. 4. This has been added as a sentence (lines 136-138). The level of the complex is lower than in Δβ cells, but maybe enhanced by the expression of either, even in the absence of subunit-γ (Fig. 4B). On Fig. 3A, the Western blot shows a very weak reaction for δ and ε in the Δγ track, which is referred to in the legend.

Point 3. In several instances the authors analyzed "immunopurified" ATP synthase (sub)complexes without stating even once against which protein(s) the antibodies for purification were directed. Without this information, the significance of the results cannot

be assessed. For example, what species of ATP synthase were purified from mitochondria lacking ATPAF2 where almost no mature ATP synthase is produced, and how are the results comparable to isolations from ∆ATPAF1 or ∆FMC1 mitochondria where some ATP synthase can assemble (Fig. 8, Fig. S18)?

In all instances, "immunopurified" samples were prepared with the commercially available ATP synthase immunocapture resin (Abcam Cat#ab109715), as noted in Methods (line 379). Whilst the specific epitope recognised by this reagent is not defined (it probably interacts with either the α- or the β-subunit), we have used it repeatedly and very successfully previously to purify assembly intermediate complexes (see refs: He et al., 2017a, 2017b, 2018, 2020; Carroll et al., 2021). In the revised manuscript, we specifically refer to this reagent in the Results section (line 115). For the individual knockout cells in Figs 8 and S18 (now Figs 6 and EV5), the relative abundance measurements of ATP synthase subunits are similar for all of the intact enzyme component proteins, with the possible exception of subunit c, which is a problematic protein as enzymic digestion of this subunit with trypsin or chymotrypsin generates very few (at most two or three) peptides that are suitable for MS-quantification. Taking the average levels of all the subunits, this limits the assembly of an intact enzyme to 4%, 30% and 40% in HAP1-∆ATPAF2, -∆ATPAF1 and -∆FMC1 cells, respectively, and 14% in HEK-∆(ATPAF1+ATPAF2+FMC1) cells, relative to the WT cell. We have now included these numbers in the Results section (lines 212-218).

Point 4. In Fig. S14, the a-delta and a-epsilon blots display a non-stoichiometric / non-correlative behaviour of the two subunits in the 200 kDa complex in different assembly factor deletion strains, in contrast to the proposed equimolar delta-epsilon complex. Additionally, the a-epsilon blot shows a complex in the last lane migrating like F1 that was not detected

with the alpha or beta antbodies even though their sensitivity appears similar. How do the authors explain these findings?

In the revised manuscript, the original Figs. S14 and S27 have been combined into new Fig. EV7. The main point is that western blotting is not an accurate or indeed appropriate method for quantitative analysis. At best, it is a semi-quantitative. However, we acknowledge that in the 200 kDa δε complex detected by the δ and ε antibodies there is an apparent discrepancy in signal intensity across the samples tracks for a 1:1 stoichiometry, with the possible exception of track ΔFMC1. Some of the δε-complex signal may arise from experimental disruption of larger complexes on native-gels, in this case the F_1-module. The MS quantification (Fig. EV7) suggests that the residual F_1 levels are 30-40% for HEK293-Δ(c+OSCP+ATPAF1) and HEK293-Δ(c+OSCP+FMC1) cells, and they fall to 7-10% for the three remaining cell lines. Therefore, the presence of an F_1 band in the last lane of the epsilon blot is not unexpected, and there is also a faint band in the adjacent track, the ΔATPAF2 track on the anti-δ panel and last two tracks of the anti-α panel.

Point 5. Regarding oxygen consumption of deletion strains (Fig. S30), the authors state on p. 11 that there is a small amount of oligomycin-sensitive oxygen consumption in strains lacking gamma, delta or epsilon subunits. However, such an effect is only visible in the data for the gamma and epsilon single deletions. The authors further conclude from these results that there is "a minor uncoupled partially assembled ATP synthase complex containing the c-subunit, but lacking an intact transmembrane proton pathway". If there is some coupling between ATP synthase activity and oxygen consumption, doesn't this rather indicate that the activity of the subcomplex is coupled to the respiratory chain and that it does have a transmembrane proton pathway? The authors should immunopurify ATP synthase in these strains to test which subunits are present.

This section has been deleted and is not in the revised manuscript. See comments above in Section 2.3.6. Reference to these data has also been removed from the Discussion section.

Minor points:

1. The handling of figures is rather inflationary and there are very few panels per figure, with some relevant results buried in the supplement. For example, Fig. S4 is very informative (the first sentence of the discussion references the results from this figure) and should become a panel of Fig. 2; Fig. S7 should be included in Fig. 3.

In the revised manuscript, multi-panel figures presenting all relevant information in a single figure have been provided in Figs. 2, 3 and 4.

2. On p. 6 the authors argue based on Fig. 3 that because the subunits of the rotor maintain intermediate levels in deletions of the alpha or beta subunits, this indicates that a central stalk complex and possibly the rotor can be assembled. This conclusion only becomes plausible once the complex isolations in Fig. 4 are shown and discussed. The authors should combine the two figures and draw their conclusion when it's appropriate.

We have changed the figures as suggested and combined the two sections *Formation of the central stalk in the absence of α- and β-subunits* and *The rotor complex,* into the one section now entitled "*The CS and the rotor complex assemble in the absence of α- and β-subunits.* The conclusions, including the interim statement that "*These observations indicate (but do not prove) that a CS-domain, and possibly an intact rotor (Fig. 1C), can be assembled in the absence of either the α- or the β-subunit"* are now placed appropriately.

3. The antibody treatments of blots should be labeled in the figure so readers don't have to search for this crucial information in the legend. E.g. in Fig. 5, rather than labeling each

panel A-F they should be labeled by the antibodies used. Similarly, for SILAC-MS analysis of different strains (e.g. in Fig. S8), the strains should be named in the figure panels.

We have added the labels as suggested (see new Figs. 4 and EV3), and, where appropriate, we have added labels to other Figures.

4. The authors state about Fig. S29 that reexpression of ATPAF2 led to a recovery of alpha, beta, gamma and epsilon subunits. However, the data show a recovery of subunits excluding alpha already with 0 mg/ml dox, and actual ATPAF2 induction leads to a reduction of gamma and epsilon. Only the ATPAF1 reexpression is doxycyclin-dependent. The authors need to discuss the issues here (leaky expression etc).

The Referee is correct to point out the impact of 'leaky' expression of ATPAF2 on the recovery of F_1-subunit levels. This is especially notable for ATPAF2 due to its dominant impact on assembly. We have added the sentence "*Recovery of subunit levels was observed even without the induction of expression of ATPAF2, probably because of 'leaky' low level expression of ATPAF2, but at levels sufficient to promote significant assembly.*" (lines 230-232).

5. The tag(s) used should be spelled out at least once in the main text or the figure legend, instead of just labeling the proteins -tag or -t.

We have added the tag description to the main text at the first mention of each tagged protein (lines 140-141; 162-163; 187-188).

6. Fig. S15 does not display any grey areas (to indicate a-helices). In Fig. S32 it is not possible to distinguish the two proteins.

We thank the referee for pointing this out. Fig. S9 (formerly S15) has been corrected to include the grey areas. Fig. S20 (formally S32) is a reproduction of the coloured confidence

representation of the Alphafold3 β:ATPAF1 binary complex, and so cannot be readily adjusted. To help the reader, we have included the following sentence at the end of the legend. "For reference, the complex depicted here is the same as shown in Figs. EV8A and EV9A, where the individual proteins are differentially colored."

7. Can the authors reference any experimental evidence for the complexes proposed in Fig. 10B+C? Since the study does not offer insight into the precise functions of ATPAF2 and FMC1, the authors should also reference relevant literature on their roles to support the statement that they, together with ATPAF1, fashion the alpha3beta3-sphere.

These figures are now Figs. EV8 and EV9, as suggested by referee 1. To address minor point 7, we have combined and edited two paragraphs in the original Discussion section describing the roles of ATPAF2 and FMC1, with relevant citations (lines 287-305).

Prof. John E. Walker
Medical Research Council
Mitochondrial Biology Unit
Wellcome Trust/MRC Building
Hills Road
Cambridge CB2 0XY
United Kingdom

18th May 2026

Re: EMBOJ-2025-123282R
Assembly of the catalytic module and the rotor of human ATP synthase

Dear Prof. Walker,

Thank you for submitting your revised manuscript on human ATPase assembly for our consideration. I appreciate your major re-organization of the study, which I feel has resulted in a much clearer presentation of the rationale behind the study and of the advances conveyed by the results. I am happy to say that this was also the opinion of the two original referees that have in the meantime reviewed the revised version. Referee 1 still notes a few specific presentational issues, which I would invite you to incorporate during a final round of minor revision - the most important one of which I would consider the conversion of Table EV1 into a 'regular' Table 1 that would appear as part of the typ-set main manuscript and thus be always accessible as reference for the readers.

In addition, there are a few other editorial issues that I would kindly ask you to attend to before resubmitting:

- Importantly, we noticed that the bar diagrams in several figures (2B; 3B-F; 6A-D; EV6E; EV7G) are based on data from only two biological replicates. Since N<3 does not allow for meaningful statistical calculations of error bars, please change the presentation to instead plotting the two respective datapoints for each bar.

- Since we cannot include colored text or symbols in tables or legends, please revise the figure legend explanation of symbols used in scatter plots and line diagrams (Figs. 3, 4, 5, EV2-EV7): either add a colored symbol explanation into the graphic itself, or change the legend itself towards textual explanation (such as, 'green squares', blue circles', etc.) instead of colored symbols as part of the legend text.

- Please double-check that each Figure panel has been referenced in the main text at least once (currently, references to Fig. 2C, Fig. 4C, Appendix Fig. S17 appear to be missing).

- In the Data Availability section, please include a hyperlink to the PRIDE database as well as accession number(s) under which the deposited mass spectrometry data can now be accessed.

- Finally, please provide suggestions for a short 'blurb' text prefacing and summing up the conceptual aspect of the study in two sentences (max. 250 characters), followed by 3-5 one-sentence 'bullet points' with brief factual statements of key results of the paper; they will form the basis of an editor-written 'Synopsis' accompanying the online version of the article (for examples, please see recent articles posted on our journal website). As image for this Synopsis, we would simply adapt the schematic from Figure 7, but if you prefer to provide a bespoke image, please make sure that it stays within the modest dimensions of (exactly) 550 x 300 pixels (width x height).

I am returning the manuscript to you for a final round of minor revision, solely to allow you to make these modifications and upload any revised files. Once we will have received them, we should be ready to swiftly proceed with formal acceptance and production of the manuscript.

Yours sincerely,

Hartmut Vodermaier

Hartmut Vodermaier, PhD
Senior Editor, The EMBO Journal
h.vodermaier@embojournal.org

*** PLEASE NOTE: All revised manuscript are subject to initial checks for completeness and adherence to our formatting guidelines. Revisions may be returned to the authors and delayed in their editorial re-evaluation if they fail to comply to the following requirements. As a first step please read our guidelines for revised submissions: https://link.springer.com/journal/44318/submission-guidelines#cms-Revised-submissions

1) Every manuscript requires a Data Availability section (even if only stating that no deposited datasets are included). Primary datasets or computer code produced in the current study have to be deposited in appropriate public repositories prior to resubmission, and reviewer access details provided in case that public access is not yet allowed.

2) Each figure legend must specify
- size of the scale bars that are mandatory for all micrograph panels
- the statistical test used to generate error bars and P-values
- the type error bars (e.g., S.E.M., S.D.)
- the number (n) and nature (biological or technical replicate) of independent experiments underlying each data point
- Figures may not include error bars for experiments with n<3; scatter plots showing individual data points should be used instead.

3) Revised manuscript text (including main tables, and figure legends for main and EV figures) has to be submitted as editable text file (e.g., .docx format). We encourage highlighting of changes (e.g., via text color) for the referees' reference.

4) Each main and each Expanded View (EV) figure should be uploaded as individual production-quality files (preferably in .eps, .tif, .jpg formats). For suggestions on figure preparation/layout, please refer to our Figure Preparation Guidelines: https://media.springernature.com/original/springer-cms/rest/v1/content/27825798/data/v1

5) Point-by-point response letters should include the original referee comments in full together with your detailed responses to them (and to specific editor requests if applicable), and also be uploaded as editable (e.g., .docx) text files.

6) Please complete our Author Checklist, and make sure that information entered into the checklist is also reflected in the manuscript; the checklist will be available to readers as part of the Review Process File.

7) All authors listed as (co-)corresponding need to deposit, in their respective author profiles in our submission system, a unique ORCiD identifier linked to their name. Please see our Guide to Authors for detailed instructions.

8) Please note that supplementary information at EMBO Press has been superseded by the 'Expanded View' for inclusion of additional figures, tables, movies or datasets; with up to five EV Figures being typeset and directly accessible in the HTML version of the article.

9) To facilitate reproducibilty and cross-laboratory adoption of methodologies, please structure the Materials & Methods section as outlined in our guide to authors, including a completed Reagents and Tools Table.

10) Digital image enhancement is acceptable practice, as long as it accurately represents the original data and conforms to community standards. If a figure has been subjected to significant electronic manipulation, this must be clearly noted in the figure legend and/or the 'Materials and Methods' section. The editors reserve the right to request original versions of figures and the original images that were used to assemble the figure. Finally, we generally encourage uploading of numerical as well as gel/blot image source data.

At EMBO Press we ask authors to provide source data for the main manuscript figures. You will receive a separate email with instructions for providing source data with your revised manuscript, including how to upload and organize the files.

In the interest of ensuring the conceptual advance provided by the work, we recommend submitting a revision within 3 months (16th Aug 2026). Please discuss the revision progress ahead of this time with the editor if you require more time to complete the revisions. Use the link below to submit your revision:

Link Not Available

Referee #1:

I thank the authors for having made the effort to substantially improve their manuscript especially in regard to clearly describing motivation, experimental approach and main findings. The abstract is now clearly written and suitable for this manuscript.

Please take the following minor comments into account for polishing your revised manuscript:

Figure 1: Please label F1 module, CS, etc. directly in the visual.

Table EV1: I think this table is well done and very helpful for the reader to navigate around the different terms used for subunits, gene names, modules and domains. Please also include the common alternative names for subunits ATP6, ATP8, j and k, that is subunit a, subunit A6L, subunit 6.8PL and subunit DAPIT. This could be done for instance in the table caption.

As this reviewer thinks that the table is rather important for making this work accessible to the wider readership of EMBO Journal, please include it in the main body of the manuscript and not as a supplementary table.

Line 97: In the presence of water and lipids there is no void. Please use a more appropriate term such as gap.

Line 107: (single or together) is a bit hard to understand. Perhaps multiple is better? "together" certainly doesn't work.

Line 155: Please delete "s".

Line 214: Suddenly using enzyme for the ATP synthase complex seems to be odd. Why not just stick to ATP synthase?

Line 342: Does "residual issue" here mean remaining issue?

Line 347: Structural lipids might show up in density maps, but I doubt that they are permanently bound. I suspect the situation might be more like structural waters that do bind in the same position, however nevertheless, can be exchanged with waters from bulk solution.

Line 171: This sentence is garbled, please re-write.

Referee #3:

The revised version of the manuscript is significantly improved both scientifically and regarding the clarity of its presentation. All my comments have been addressed adequately. This thorough study is now ready for publication.

Editorial and Referee responses for EMBOJ-2025-123282R

Responses to the editorial issues.

1. The bar diagrams in Figures 2B, 3B-F, 6A-D, EV6E and EV7G now have the two individual datapoints shown, without the adjoining lines. The accompanying Figure legends have been altered to reflect these changes.

2. Coloured symbols in the Figure legends have been removed and replaced with text.

3. All Figure panels are referenced in the text.

4. Hyperlinks to the PRIDE database repository are now added in the Data Availability section.

Responses to Referee 1.

1. Figure 1. The modules are now labelled on Fig.1, as requested.

2. Table 1 has been moved to the main paper. The alternative names for subunits are given as a footnote to the Table.

3. Line 97. According to the Oxford Dictionary of the English language, “gap” is a synonym for “void”. The meaning is perfectly clear and therefore, “void” has been retained; “protein” as in “protein void” has been added for absolute clarity.

Line 107. “Singly or together” is perfectly clear and understandable, and has been retained.

Line 155. Deleted the “s”.

Line 214. ATP synthase is an enzyme. Therefore, “enzyme” is entirely appropriate in this context and has been retained.

Line 342. “Residual” is appropriate and the meaning is clear, and therefore it has been retained.

Line 347. The referee is wrong. In the structure of the bovine enzyme (Spikes et al), lipids are found inside the “wedge” and are permanently and irreversibly bound, as described in great detail in the publication. How and when they are inserted during assembly of the enzyme remains to be established.

Line 171. There was an error in this sentence that we have corrected. It should now be understandable.

EMBO Press Author Checklist

Corresponding Author Name: John E. Walker	
Journal Submitted to: The EMBO Journal	
Manuscript Number: EMBOJ-2025-123282	

USEFUL LINKS FOR COMPLETING THIS FORM
The EMBO Journal - Author Guidelines
EMBO Reports - Author Guidelines
Molecular Systems Biology - Author Guidelines
EMBO Molecular Medicine - Author Guidelines

Reporting Checklist for Life Science Articles (updated January
This checklist is adapted from Materials Design Analysis Reporting (MDAR) Checklist for Authors. MDAR establishes a minimum set of requirements in transparent reporting in the life sciences (see Statement of Task: 10.31222/osf.io/9sm4x). Please follow the journal's guidelines in preparing your manuscript.
Please note that a copy of this checklist will be published alongside your article.

Abridged guidelines for figures
1. Data
The data shown in figures should satisfy the following conditions:
- the data were obtained and processed according to the field's best practice and are presented to reflect the results of the experiments in an accurate and unbiased manner.
- ideally, figure panels should include only measurements that are directly comparable to each other and obtained with the same assay.
- plots include clearly labeled error bars for independent experiments and sample sizes. Unless justified, error bars should not be shown for technical
- if n<5, the individual data points from each experiment should be plotted. Any statistical test employed should be justified.
- Source Data should be included to report the data underlying figures according to the guidelines set out in the authorship guidelines on Data

2. Captions
Each figure caption should contain the following information, for each panel where they are relevant:
- a specification of the experimental system investigated (eg cell line, species name).
- the assay(s) and method(s) used to carry out the reported observations and measurements.
- an explicit mention of the biological and chemical entity(ies) that are being measured.
- an explicit mention of the biological and chemical entity(ies) that are altered/varied/perturbed in a controlled manner.
- the exact sample size (n) for each experimental group/condition, given as a number, not a range;
- a description of the sample collection allowing the reader to understand whether the samples represent technical or biological replicates (including how many animals, litters, cultures, etc.).
- a statement of how many times the experiment shown was independently replicated in the laboratory.
- definitions of statistical methods and measures:
  - common tests, such as t-test (please specify whether paired vs. unpaired), simple χ2 tests, Wilcoxon and Mann-Whitney tests, can be unambiguously identified by name only, but more complex techniques should be described in the methods section;
  - are tests one-sided or two-sided?
  - are there adjustments for multiple comparisons?
  - exact statistical test results, e.g., P values = x but not P values < x;
  - definition of 'center values' as median or average;
  - definition of error bars as s.d. or s.e.m.

Please complete ALL of the questions below.
Select "Not Applicable" only when the requested information is not relevant for your study.

Materials

Newly Created Materials	Information included in the manuscript?	In which section is the information available? (Reagents and Tools Table, Materials and Methods, Figures, Data Availability Section)
New materials and reagents need to be available; do any restrictions apply?	Not Applicable	

Antibodies	Information included in the manuscript?	In which section is the information available? (Reagents and Tools Table, Materials and Methods, Figures, Data Availability Section)
For **antibodies** provide the following information:<br>- Commercial antibodies: RRID (if possible) or supplier name, catalogue number and or/clone number<br>- Non-commercial: RRID or citation	Yes	Reagents and Tools Table

DNA and RNA sequences	Information included in the manuscript?	In which section is the information available? (Reagents and Tools Table, Materials and Methods, Figures, Data Availability Section)
**Short novel DNA or RNA including primers, probes**: provide the sequences.	Yes	Appendix Tables S6, S7 and S8

Cell materials	Information included in the manuscript?	In which section is the information available? (Reagents and Tools Table, Materials and Methods, Figures, Data Availability Section)
**Cell lines:** Provide species information, strain. Provide accession number in repository **OR** supplier name, catalog number, clone number, and/**OR** RRID.	Yes	Reagents and Tools Table
**Primary cultures:** Provide species, strain, sex of origin, genetic modification status.	Not Applicable	
Report if the cell lines were recently **authenticated** (e.g., by STR profiling) and tested for mycoplasma contamination.	Yes	Materials and Methods

Experimental animals	Information included in the manuscript?	In which section is the information available? (Reagents and Tools Table, Materials and Methods, Figures, Data Availability Section)
**Laboratory animals or Model organisms:** Provide species, strain, sex, age, genetic modification status. Provide accession number in repository **OR** supplier name, catalog number, clone number, **OR** RRID.	Not Applicable	
**Animal observed in or captured from the field:** Provide species, sex, and age where possible.	Not Applicable	
Please detail **housing and husbandry conditions**.	Not Applicable	

Plants and microbes	Information included in the manuscript?	In which section is the information available? (Reagents and Tools Table, Materials and Methods, Figures, Data Availability Section)
**Plants:** provide species and strain, ecotype and cultivar where relevant, unique accession number if available, and source (including location for collected wild specimens).	Not Applicable	
**Microbes:** provide species and strain, unique accession number if available, and source.	Yes	Reagents and Tools Table

Human research participants	Information included in the manuscript?	In which section is the information available? (Reagents and Tools Table, Materials and Methods, Figures, Data Availability Section)
If collected and within the bounds of privacy constraints report on age, sex and gender or ethnicity for all study participants.	Not Applicable	

Core facilities	Information included in the manuscript?	In which section is the information available? (Reagents and Tools Table, Materials and Methods, Figures, Data Availability Section)
If your work benefited from core facilities, was their service mentioned in the acknowledgments section?	Not Applicable	

Design

Study protocol	Information included in the manuscript?	In which section is the information available? (Reagents and Tools Table, Materials and Methods, Figures, Data Availability Section)
If study protocol has been **pre-registered, provide DOI in the manuscript**. For clinical trials, provide the trial registration number **OR** cite DOI.	Not Applicable	
Report the **clinical trial registration number** (at ClinicalTrials.gov or equivalent), where applicable.	Not Applicable	

Laboratory protocol	Information included in the manuscript?	In which section is the information available? (Reagents and Tools Table, Materials and Methods, Figures, Data Availability Section)
Provide DOI OR other citation details if **external detailed step-by-step protocols** are available.	Not Applicable	

Experimental study design and statistics	Information included in the manuscript?	In which section is the information available? (Reagents and Tools Table, Materials and Methods, Figures, Data Availability Section)
Include a statement about **sample size** estimate even if no statistical methods were used.	Not Applicable	
Were any steps taken to minimize the effects of subjective bias when allocating animals/samples to treatment (e.g. **randomization procedure**)? If yes, have they been described?	Not Applicable	
Include a statement about **blinding** even if no blinding was done.	Not Applicable	
Describe **inclusion/exclusion criteria** if samples or animals were excluded from the analysis. Were the criteria pre-established?<br><br>If sample or data points were omitted from analysis, report if this was due to attrition or intentional exclusion and provide justification.	Yes	Figure 3H has one data point excluded from the 'contaminant' top left quadrant, and this is noted in the figure legend. Figures EV3A and EV3C also have one data point each for 'non-binding' proteins excluded from the display, which is noted in the legend.
For every figure, are **statistical tests** justified as appropriate? Do the data meet the assumptions of the tests (e.g., normal distribution)? Describe any methods used to assess it. Is there an estimate of variation within each group of data? Is the variance similar between the groups that are being statistically compared?	Yes	Where statistical significance for protein abundance changes is referred to in the manuscript, this is Significance B, calculated with a Benjamini-Hochberg correction (p=0.05) as noted in the Protein quantitation method section. The lines on the histograms are only to show the range of the two relative abundance values determined in the SILAC-MS analyses. This is noted in all figure legends.

Sample definition and in-laboratory replication	Information included in the manuscript?	In which section is the information available? (Reagents and Tools Table, Materials and Methods, Figures, Data Availability Section)
In the figure legends: state number of times the experiment was **replicated** in laboratory.	Yes	Figure legends
In the figure legends: define whether data describe **technical or biological replicates**.	Yes	Biolgical replicates were performed for SILAC-MS analyses, as stated in the Figure legends.

Ethics

Ethics	Information included in the manuscript?	In which section is the information available? (Reagents and Tools Table, Materials and Methods, Figures, Data Availability Section)
Studies involving **human participants**: State details of **authority granting ethics approval** (IRB or equivalent committee(s), provide reference number for approval.	Not Applicable	
Studies involving **human participants**: Include a statement confirming that **informed consent** was obtained from all subjects and that the experiments conformed to the principles set out in the WMA Declaration of Helsinki and the Department of Health and Human Services Belmont Report.	Not Applicable	
Studies involving **human participants:** For publication of **patient photos**, include a statement confirming that consent to publish was obtained.	Not Applicable	
Studies involving experimental **animals**: State details of **authority granting ethics approval** (IRB or equivalent committee(s), provide reference number for approval. Include a statement of compliance with ethical regulations.	Not Applicable	
Studies involving **specimen and field samples:** State if relevant **permits** obtained, provide details of authority approving study; if none were required, explain why.	Not Applicable	

Dual Use Research of Concern (DURC)	Information included in the manuscript?	In which section is the information available? (Reagents and Tools Table, Materials and Methods, Figures, Data Availability Section)
Could your study fall under dual use research restrictions? Please check biosecurity documents and list of **select agents and toxins** (CDC): https://www.selectagents.gov/sat/list.htm	Not Applicable	
If you used a select agent, is the security level of the lab appropriate and reported in the manuscript?	Not Applicable	
If a study is subject to dual use research of concern regulations, is the name of the **authority granting approval and reference number** for the regulatory approval provided in the manuscript?	Not Applicable	

Reporting

The MDAR framework recommends adoption of discipline-specific guidelines, established and endorsed through community initiatives. Journals have their own policy about requiring specific guidelines and recommendations to complement MDAR.

Adherence to community standards	Information included in the manuscript?	In which section is the information available? (Reagents and Tools Table, Materials and Methods, Figures, Data Availability Section)
State if relevant guidelines or checklists (e.g., **ICMJE, MIBBI, ARRIVE, PRISMA**) have been followed or provided.	Not Applicable	
For **tumor marker prognostic studies,** we recommend that you follow the **REMARK** reporting guidelines (see link list at top right). See author guidelines, under 'Reporting Guidelines'. Please confirm you have followed these guidelines.	Not Applicable	
For **phase II and III randomized controlled trials**, please refer to the **CONSORT** flow diagram (see link list at top right) and submit the CONSORT checklist (see link list at top right) with your submission. See author guidelines, under 'Reporting Guidelines'. Please confirm you have submitted this list.	Not Applicable	

Data Availability

Data availability	Information included in the manuscript?	In which section is the information available? (Reagents and Tools Table, Materials and Methods, Figures, Data Availability Section)
Have **primary datasets** been deposited according to the journal's guidelines (see 'Data Deposition' section) and the respective accession numbers provided in the Data Availability Section?	Yes	Mass spectrometry raw data files and MaxQuant evidence files are available at the PRIDE data repository as noted in the Data availability section.
Were **human clinical and genomic datasets** deposited in a public access-controlled repository in accordance to ethical obligations to the patients and to the applicable consent agreement?	Not Applicable	
Are **computational models** that are central and integral to a study available without restrictions in a machine-readable form? Were the relevant accession numbers or links provided?	Not Applicable	
If publicly available data were reused, provide the respective **data citations in the reference list**.	Not Applicable	
