# Supplementary material for: Assembly of the catalytic module and the rotor of human ATP synthase
Source: EMBO J. 2026 Jun 26;45(15):5482–513. doi: 10.1038/s44318-026-00842-9 (PMC13434801; doi:10.1038/s44318-026-00842-9)
Supplement: Supplementary file 4 [file 44318_2026_842_MOESM3_ESM.zip › Figure 1/Note for Figure 1.rtf]

The PyMOL molecular visualisation tool was used with PDB file 6ZQM to generate figures 1A-E.
