# Supplementary material for: Assembly of the catalytic module and the rotor of human ATP synthase
Source: EMBO J. 2026 Jun 26;45(15):5482–513. doi: 10.1038/s44318-026-00842-9 (PMC13434801; doi:10.1038/s44318-026-00842-9)
Supplement: Supplementary file 10 [file 44318_2026_842_MOESM9_ESM.zip › Figure 7/Notes for Figure 7.rtf]

The Affinity Designer program was used to generate figures 7A and 7B.
